# Overcoming Artificial Structures in Resolution‐Enhanced Hi‐C Data by Signal Decomposition and Multi‐Scale Attention

**DOI:** 10.1002/advs.202524180

**Published:** 2026-09-08

**Authors:** Qinyao Li, Kelly Yichen Li, Chiara Nicoletti, Stephen Kwok‐Wing Tsui, Pier Lorenzo Puri, Qin Cao, Kevin Y. Yip

**Affiliations:** ^1^ Department of Computer Science and Engineering The Chinese University of Hong Kong New Territories Hong Kong SAR China; ^2^ Center for Data Science and Artificial Intelligence Sanford Burnham Prebys Medical Discovery Institute La Jolla California USA; ^3^ Center for Cardiovascular and Muscular Diseases Sanford Burnham Prebys Medical Discovery Institute La Jolla California USA; ^4^ School of Biomedical Sciences The Chinese University of Hong Kong New Territories Hong Kong SAR China; ^5^ Cancer Genome and Epigenetics Program, NCI‐Designated Cancer Center Sanford Burnham Prebys Medical Discovery Institute La Jolla California USA; ^6^ Shenzhen Research Institute The Chinese University of Hong Kong Shenzhen China; ^7^ Center for Neurologic Diseases Sanford Burnham Prebys Medical Discovery Institute La Jolla California USA

**Keywords:** artifacts, chromatin interactions, data resolution, Hi‐C, machine learning

## Abstract

Computational enhancement is an important strategy for inferring high‐resolution features from genome‐wide chromosome conformation capture (Hi‐C) data, which typically have limited resolution. Deep learning has been highly successful in this task but we show that it creates prevalent artificial structures in the enhanced data due to the need to divide the large contact matrix into small patches. In addition, previous deep learning methods largely focus on local patterns, which cannot fully capture the complexity of Hi‐C data. Here we propose Smooth, High‐resolution, and Accurate Reconstruction of Patterns (SHARP) for enhancing Hi‐C data. It uses the novel approach of decomposing the data into three types of signals, due to one‐dimensional proximity, contiguous domains, and other fine structures, respectively, and applies deep learning only to the third type of signals, such that enhancement of the first two is unaffected by the patches. For the deep learning part, SHARP uses both local and global attention mechanisms to capture multi‐scale contextual information. We compare SHARP with state‐of‐the‐art methods extensively, including application to data from new samples and another species, and show that SHARP has superior performance in terms of resolution enhancement accuracy, avoiding creation of artificial structures, identifying significant interactions, and enrichment in chromatin states.

## Graphical abstract

Deep‐learning‐based signal enhancement is an effective way to recover high‐resolution details from a low‐resolution chromatin contact map. However, due to computational challenges, existing methods commonly divide up the contact map into small patches and create artificial discontinuities at patch boundaries. SHARP is a new method that effectively recovers high‐resolution details with minimal artifacts at patch boundaries.

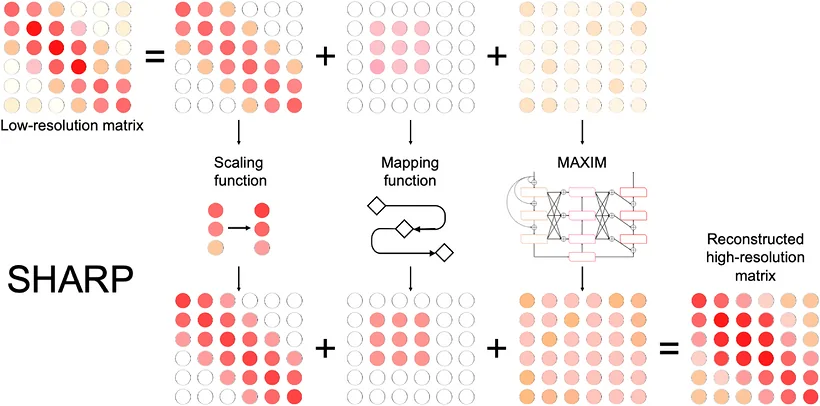

## 1 Introduction

In mammalian genomes, long DNA molecules fold in three‐dimensional (3D) space into compact structures such that they can be packed into the cell nucleus. Understanding such spatial structures can provide insights into complex relationships between genome architecture, gene activities, and functional states of the cell [1, 2, 3, 4, 5]. High‐throughput chromosome conformation capture (Hi‐C) [6] is the most commonly used method for probing the overall 3D genome architecture by detecting pair‐wise chromatin contacts across the entire genome. The resulting data is represented by a contact matrix, which records interaction frequencies between equally sized genomic bins. Typical bin size used in published studies ranges from 1 kb to 1 Mb. In order to have sufficiently reliable contact counts, it has been proposed that the resolution of a Hi‐C contact matrix is the smallest bin size such that ≥80% of the bins have ≥ 1000 contacts [7]. For the human and mouse genomes, Hi‐C data with a resolution of 40 kb or lower is usually considered low‐resolution [8, 9], whereas data with a resolution of 5 kb or higher is usually considered high‐resolution [9, 10].

Since a linear increase in the resolution requires a quadratic increase in the number of sequencing reads [11], few existing Hi‐C data sets have very high resolution, but it is required for detecting fine‐scaled local structures. High‐resolution data are also particularly important for identifying regulatory regions and their contacts with potential target genes in normal and disease states [12, 13, 14, 15] since there can be multiple regulatory elements and genes within the vicinity of a small genomic region.

To reconstruct high‐resolution genome structures from low‐resolution Hi‐C data, computational methods have been employed. The state‐of‐the‐art approach is to treat the Hi‐C contact matrix as an image and define the reconstruction problem as image resolution enhancement [8, 16, 17, 18, 19], using deep artificial neural network (ANN) models such as convolutional neural networks (CNNs) [20] or generative adversarial networks (GANs) [21].

Due to the large size of the contact matrix, all these methods have to divide it into small regular‐sized sub‐matrices (to be referred to as “patches” hereafter) and apply the model to each of them separately. As to be shown in Results, the boundaries of these patches frequently create discontinuities in the reconstructed high‐resolution contact matrix and consequently, artificial structures such as fake topologically associating domains (TADs) [22, 23, 24] that do not actually exist are wrongly called.

In this work, we propose Smooth, High‐resolution, and Accurate Reconstruction of Patterns (SHARP), a novel method for enhancing resolution of Hi‐C contact matrices that avoids creating artificial structures. We demonstrate that SHARP outperforms current state‐of‐the‐art methods in terms of both resolution enhancement and avoidance of artificial structures across various data sets and settings. As a result, downstream analyzes are also more accurate with the SHARP‐enhanced Hi‐C contact matrices, such as detection of significant interactions and their chromatin states.

## 2 Results

### 2.1 Overview of SHARP

**FIGURE 1:**
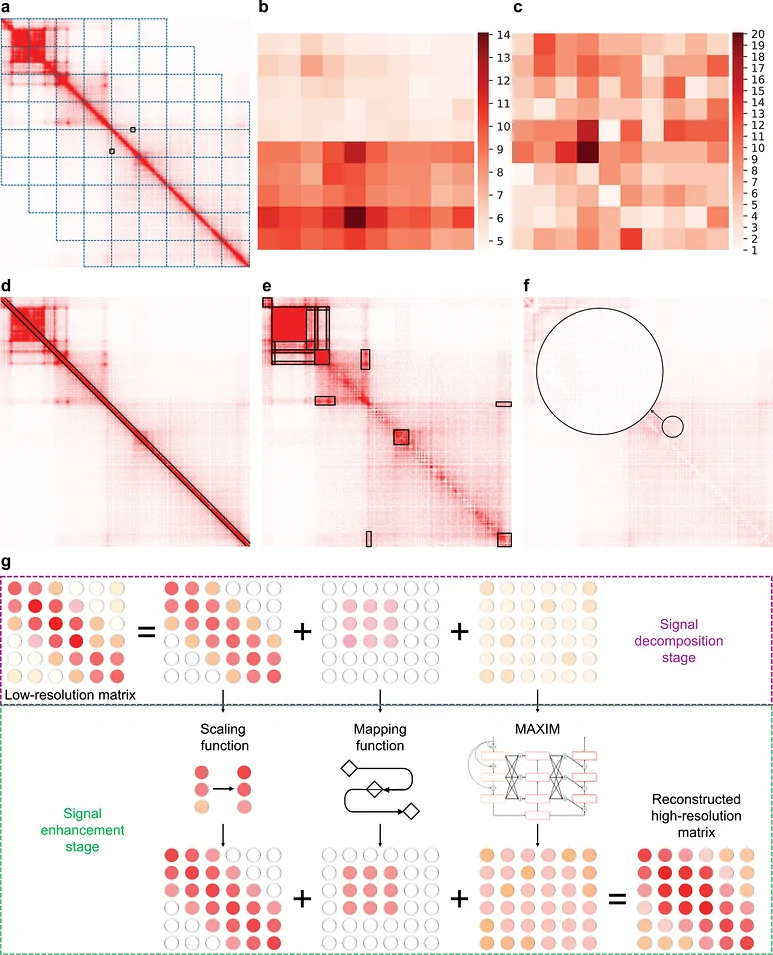
Overview of SHARP. (a), An example region (Chr20:53.4‐56.3 Mb) showing that boundaries of 320 kb × 320 kb patches (squares wrapped by blue dashed lines) do not align with real structures in the actual high‐resolution GM12878 contact matrix. (b), Resolution‐enhanced contact matrix produced by the published method HiCNN2‐3 [25] of an example area in the contact matrix (Chr20:54.935 Mb‐54.985 Mb × Chr20:54.695 Mb‐54.745 Mb, marked by the black boxes in Panel a). The horizontal line in the middle is the patch boundary. (c), The actual high‐resolution contact matrix of the same area depicted in Panel b. (d–f), Decomposition of the contact matrix into signals due to 1D proximity (diagonal band in Panel d), contiguous domains (rectangles in Panel e), and other fine structures (Panel f). The three panels show the original matrix, the matrix after subtracting out signals due to 1D proximity, and the matrix after further subtracting out signals due to contiguous domains, respectively. The large circle in Panel f provides a zoomed‐in view of the area indicated by the small circle. (g), The two stages of SHARP during model training.

The artificial structures created by existing Hi‐C resolution enhancement methods originate from the fact that the patches they define as input units do not align with the actual structures observed in the contact matrix (Figure 1a). As a result, in the reconstructed high‐resolution matrix, values on the two sides of a patch boundary can take on very different values since the two patches are enhanced by the model separately (Figure 1b), which is an artifact that does not happen in the actual high‐resolution contact matrix (Figure 1c). Some existing methods attempt to solve this problem by defining overlapping patches [18, 25] but that creates substantial computational overhead and adds extra complexity in merging the reconstruction results of overlapping patches. On the other hand, adding a post‐hoc smoothing step is also not a good solution since it can destroy real structures that happen to align with the patches.

SHARP tackles this problem by modeling the Hi‐C contact matrix as the superposition of signals caused by three types of chromatin contacts, which correspond to different organizational scales of genome architecture. Specifically: (1) Contacts due to one‐dimensional (1D) proximity create strong signals close to the main diagonal of the matrix [26] (Figure 1d). These signals reflect the distance‐dependent contact decay driven by chromatin polymer folding. (2) Contacts due to contiguous domains create elevated signals in on‐diagonal and off‐diagonal rectangular blocks (representing within‐domain and between‐domain contacts, respectively) compared to their background (Figure 1e). These signals capture domain‐level chromatin organization, such as TADs, where genomic regions exhibit preferential interactions. (3) Contacts due to other fine‐scale structures create highly localized and more subtle signals (Figure 1f). These focal interactions generally represent specific regulatory contacts, including individual chromatin loops and enhancer‐promoter interactions.

Specifically, the procedure of SHARP is divided into a signal decomposition stage and a signal enhancement stage (Figure 1g, Methods). In the signal decomposition stage, signals due to 1D proximity are modeled by an analytical function [27] while signals due to contiguous domains are detected by our novel block detection and modeling algorithms (Methods). We allow the blocks to be overlapping and nested due to complex arrangements of structures in the 3D genome. After subtracting out these two types of signals, the remaining contact matrix is expected to be more uniform and contains mostly subtle local structures (Figure 1f). Then in the signal enhancement stage, the three types of signals are enhanced separately and then added together to form the high‐resolution matrix: Signals due to 1D proximity are scaled to match the expected level in the high‐resolution matrix; signals due to contiguous domains are enhanced by a mapping function; the remaining signals are enhanced using an ANN (Methods). Since the first two types of signals are identified from the whole contact matrix directly without dividing it into patches, the final resolution‐enhanced matrix is much less affected by the patch boundaries.

For the ANN, we chose the Multi‐Axis Multi‐Layer Perceptron for Image Processing (MAXIM) architecture [28], which uses both local attention and global attention mechanisms to effectively capture multi‐scale contextual information and detailed textures, thereby achieving excellent performance in image enhancement tasks. During model training, parameters of the models for all three types of signals are learned based on paired high‐resolution and low‐resolution contact matrices of the same biological sample. The learned models can then be applied to enhance the resolution of a low‐resolution contact matrix from another sample directly.

### 2.2 SHARP Reconstructs High‐Resolution Hi‐C Contact Matrices Accurately

**FIGURE 2:**
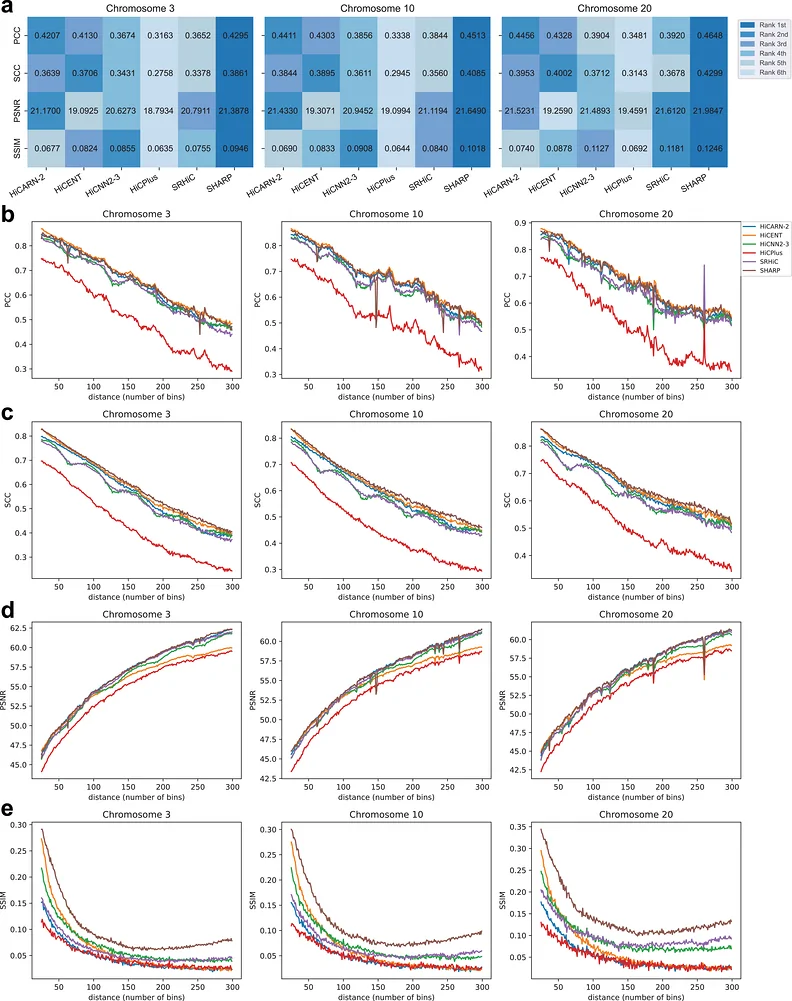
Resolution enhancement performance of SHARP as compared to five other methods. (a), Performance of the different methods when applied to the left‐out chromosomes based on four different performance measures. For all four measures, a larger value corresponds to better performance. (b–e), Performance of the methods for region pairs at different genomic distances based on PCC (b), SCC (c), PSNR (d), and SSIM (e).

To evaluate the effectiveness of SHARP, following previous studies [8, 17, 18, 19, 25, 29, 30, 31], we applied it to enhance a low‐resolution contact matrix created by down‐sampling an actual high‐resolution contact matrix produced from human GM12878 cells [7]. We trained the models using data in both the high‐resolution and low‐resolution matrices from 15 chromosomes, selected the best model during the training process using 4 other chromosomes, and finally evaluated the enhancement performance using the remaining 3 left‐out chromosomes (Chr 3, 10, and 20) (Methods). For benchmarking purpose, we also applied five state‐of‐the‐art Hi‐C resolution enhancement methods in the same way, namely HiCARN‐2 [19], HiCENT [32], HiCNN2‐3 [25], HiCPlus [8], and SRHiC [33]. Based on four standard performance measures (Pearson correlation coefficient [PCC], Spearman correlation coefficient [SCC], peak signal‐to‐noise ratio [PSNR] and structural similarity index measure [SSIM]; Methods), SHARP consistently outperformed these five methods in all cases (Figure 2a).

We further compared the performance of the six methods on pairs of genomic bins at different 1D distances separately. The results show that SHARP stably outperformed the other methods at almost all distance values for all the considered metrics (Figure 2b–e). HiCENT's performance was close to SHARP in terms of PCC, but it had low performance in terms of PSNR and SSIM.

To assess the robustness of these results, we repeated the comparisons using 1) low‐resolution contact matrices down‐sampled from the high‐resolution GM12878 matrix at other sampling rates (1/50 [∼50 kb resolution] and 1/100 [∼90 kb resolution], instead of the original 1/150 [∼200 kb resolution]), 2) an actual low‐resolution contact matrix from a GM12878 Hi‐C data set with low sequencing depth (∼200 kb resolution), instead of one down‐sampled from a high‐resolution matrix, and 3) high‐resolution and down‐sampled Hi‐C data of another cell line (H1‐hESC, instead of the original GM12878). The results show that SHARP also outperformed the other methods in most cases (Figures S1–S3).

### 2.3 SHARP Avoids Creation of Artificial Structures

**FIGURE 3:**
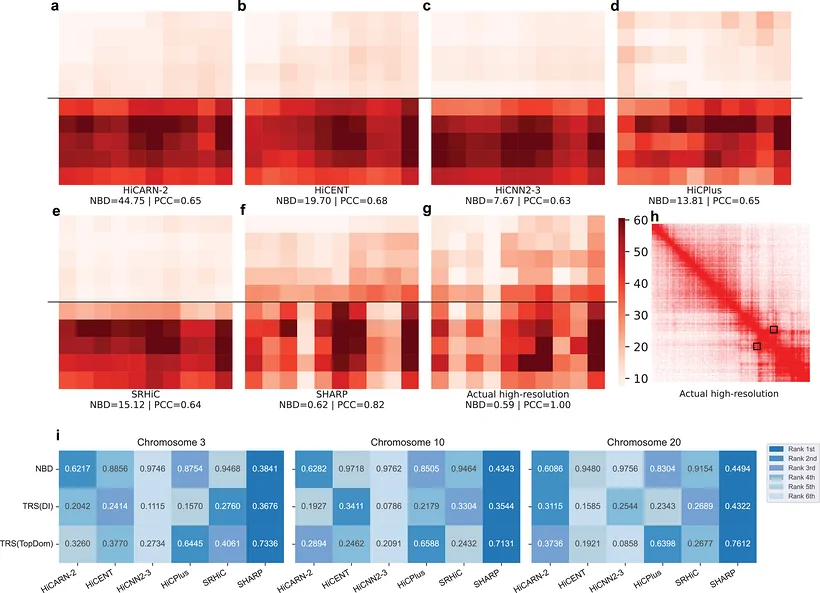
Artificial structures in resolution‐enhanced Hi‐C contact matrices and their avoidance by SHARP. (a–f), An example area (Chr18:79.335 Mb‐79.385 Mb × Chr18:79.565 Mb‐79.615 Mb) of the resolution‐enhanced contact matrix produced by HiCARN‐2 (a), HiCENT (b), HiCNN2‐3 (c), HiCPlus (d), SRHiC (e), and SHARP (f). In all cases, the above and lower halves belong to two different patches. Local PCC and NBD are reported. (g), The actual high‐resolution contact matrix of the same area. (h), Location of the area in the whole high‐resolution matrix of the chromosome marked by the black boxes. (i), Performance of the different methods when applied to the left‐out chromosomes based on three different measures. For the NBD measure, a smaller value corresponds to better avoidance of artificial structures; for the TRS measures, a larger value corresponds to better avoidance of artificial TADs.

The four standard performance measures used in the comparisons above do not specifically quantify artifacts caused by the patches. We examined the resolution‐enhanced contact matrices produced by the five existing methods and found artifacts at patch boundaries in all of them that do not appear in the actual high‐resolution contact matrix (Figure 3a–h, Figures S4–S6). To systematically evaluate the prevalence of this issue, we invented the Normalized Boundary Difference (NBD) measure, which computes the average difference between values across a patch boundary normalized by the average difference between immediate neighboring values within the patch (Figure S7, Methods). A large NBD value indicates abrupt changes across patch boundaries that could be due to artifacts. Based on the NBD measure, SHARP produced a much lower level of artifacts than the other methods (Figure 3i).

To evaluate the downstream impacts of these artificial structures, we called TADs from the resolution‐enhanced contact matrices and compared them with the TADs called from the actual high‐resolution contact matrix. We defined the TAD‐related score (TRS) to quantify how well artificial TADs caused by the patch boundaries are avoided (Methods). A large TRS value indicates better avoidance of these artificial TADs. Comparing the TRS values based on TADs called by two different methods (Directionality Index [DI] [22] and TopDom [34]), SHARP produced fewer artificial TADs at the patch boundaries than the other five methods (Figure 3i).

We also visualized the Hi‐C contact matrices generated by the six methods near patch boundaries (Figures S4–S6). Compared with the other methods, which exhibited clearly visible patch boundaries, SHARP reconstructed smoother transitions across boundaries and produced reconstructions that are more consistent with the actual high‐resolution Hi‐C contact maps. Visualization at a larger genomic scale (Figure S8) further suggests that SHARP introduced fewer artificial boundaries across patches and achieved more accurate reconstruction.

Again, we repeated the comparisons with different down‐sampling rates, an actual low‐resolution contact matrix, and data from another cell line, which confirmed the robustness of SHARP in avoiding the creation of artificial structures at patch boundaries (Figures S9–S11).

To evaluate the role of signal decomposition and the attention backbone in SHARP, we conducted ablation studies comparing SHARP with two simplified variants. First, we removed the signal decomposition stage and directly trained the MAXIM model on the contact matrix (“SHARP‐NoDecomposition”, Methods). Second, we retained the signal decomposition module but replaced the MAXIM backbone with a simple convolutional network (“SHARP‐CNN”, Methods).

The results show that SHARP‐NoDecomposition introduced more artificial structures, confirming that signal decomposition is critical for avoiding artificial boundaries (Figure S12). In contrast, SHARP‐CNN achieved similar performance to SHARP in avoiding artificial boundaries (Figure S14b), further supporting the role of signal decomposition in avoiding artificial structures.

In terms of resolution enhancement accuracy, SHARP outperformed SHARP‐CNN substantially (Figure S14a), demonstrating the benefit of the MAXIM attention backbone. Notably, SHARP‐NoDecomposition still outperformed existing methods (Figure S13), suggesting that the use of both local and global attention contributes to performance gains independent of signal decomposition.

### 2.4 Identification of Statistically Significant Interactions

**FIGURE 4:**
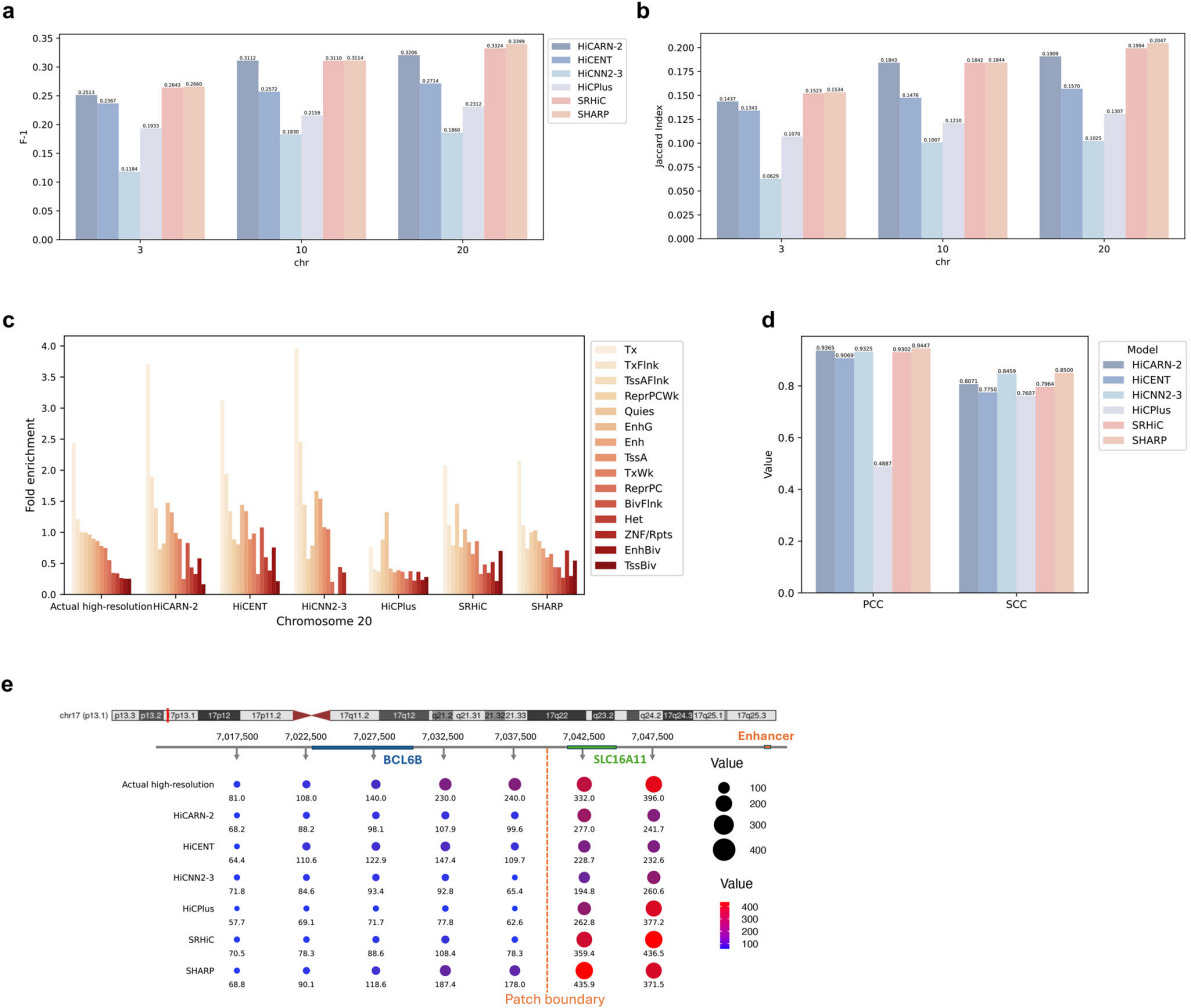
Significant chromatin interactions identified by SHARP as compared to five other methods. (a,b) Comparing the statistically significant chromatin interactions identified from the resolution‐enhanced contact matrices produced by the methods with those identified from the actual high‐resolution matrix, quantified by the F‐1 score (a) or Jaccard Index (b). (c) Enrichment of the identified chromatin interactions at different chromatin states of Chr 20, including strong transcription (Tx), quiescent/low (Quies), weak repressed Polycomb (ReprPCWk), transcription at gene 5' and 3' (TxFlnk), weak transcription (TxWk), flanking active TSS (TssAFlnk), enhancers (Enh), genic enhancers (EnhG), active TSS (TssA), bivalent/poised TSS (TSSBiv), repressed Polycomb (ReprPC), flanking bivalent TSS/Enh (BivFlnk), ZNF genes + repeats (ZNF/Rpts), bivalent enhancer (EnhBiv), and heterochromatin (Het). The chromatin states are sorted in descending order of their enrichment values according to the actual high‐resolution matrix. (d) Correlation of the enrichment values with those computed based on significant interactions identified from the actual high‐resolution matrix. (e) Specific CRE‐gene interactions identified by the different methods.

An important use of resolution‐enhanced Hi‐C contact matrices is identifying high‐resolution functional chromatin interactions. To evaluate SHARP's ability to facilitate this, we started by comparing the statistically most significant interactions in the resolution‐enhanced contact matrices with those in the actual high‐resolution contact matrix (Methods). These interactions are the ones with the strongest enrichment of contact frequencies as compared to random expectation, and they represent promising candidates of functionally important interactions [35]. Based on two different evaluation measures, the significant interactions identified by FitHiC2 [36] produced by SHARP are most consistent with those identified from the actual high‐resolution matrix no matter the whole reconstructed matrix was considered (Figure 4a,b) or only the imputed regions (Figure S15). SHARP also achieved the highest performance when applying the same analysis to the interactions identified by HiCCUPS [7]. However, the total number of interactions fluctuated considerably, making the results insufficiently robust.

Next, we examined the chromatin states of loci involved in these significant interactions (Methods). Judged by fold enrichment values, all methods produced significant interactions in chromatin states resembling those obtained from the actual high‐resolution contact matrix (Figure 4c, Figure S16a,b). However, some important differences are also observed. For example, in Chromosome 20, comparing the enrichment of the significant interactions in the strong transcription state (“Tx”), SHARP's results are closest to the actual high‐resolution matrix while the other methods produced enrichment values either too large (HiCARN‐2, HiCENT and HiCNN2‐3) or too small (HiCPlus and SRHiC) (Figure 4c). Quantitatively, the enrichment values computed from the actual high‐resolution Hi‐C matrix are most consistently correlated with the enrichment values of SHARP (Figure 4d and Figure S16c,d). To assess reproducibility, we evaluated interaction consistency across four replicates of the high‐resolution GM12878 data and observed highly consistent F1 scores, Jaccard index, and chromatin state profiles, supporting the robustness of interaction calls across replicates (Figures S17 and S18).

We also used independently supported interactions between genes and cis‐regulatory elements (CREs) to compare the significant chromatin interactions identified from the different resolution‐enhanced contact matrices (Figures 4e, S19 and S20). Figure 4e shows an example of an enhancer (Chr17:7,130,223‐7,134,160) cataloged in multiple databases (NCBI Reference Sequence [37], FANTOM5 [38], and ENCODE [39]) that interacts with two protein coding genes, *BCL6B* and *SLC16A11* [40]. Both interactions are supported by high gene association scores. In both the actual high‐resolution Hi‐C matrix and the resolution‐enhanced matrices reconstructed by all six methods, the enhancer has the strongest interaction with *SLC16A11*. This is not surprising because *SLC16A11* is relatively close to the enhancer and they are both in the same patch defined by the resolution enhancement methods. However, only SHARP was able to infer a strong interaction between the enhancer and *BCL6B*, which reside in the same TAD, and their interaction is supported by the actual high‐resolution Hi‐C matrix. The other five methods likely missed this interaction because the enhancer and the *BCL6B* locus were located in two different patches, which created difficulties for them to determine the interaction frequency accurately relative to the interaction frequency between the enhancer and the *SLC16A11* locus. Figures S19 and S20 provide two additional examples in which only SHARP successfully reconstructed gene‐CRE interactions that span across patch boundaries.

### 2.5 Applicability of the Human Hi‐C Models to Mouse Data and Micro‐C Data

In the actual use of a Hi‐C resolution‐enhancement method, it is given only a low‐resolution matrix and has to directly enhance it without access to any high‐resolution Hi‐C data from the same sample. This requires applying a model previously trained on data from another sample. To assess such generalizability of the SHARP models, we performed model training using matched high‐ and low‐resolution Hi‐C contact matrices from human GM12878 cells and applied the models to enhance a low‐resolution Hi‐C matrix from mouse embryonic stem cells (mESCs) and a low‐resolution Micro‐C matrix [41] from HFF‐hTERT clone 6 cells (HFFc6). This design allows us to evaluate whether SHARP can be transferred across cell types (from GM12878 lymphoblastoid cells to mESC embryonic stem cells and HFFc6 foreskin fibroblast cells), across species (from human GM12878 cells to mouse mESCs), and across experimental protocols (from Hi‐C data to Micro‐C data). For benchmarking purpose, the same procedure was applied to other models, including HiCARN‐2, HiCENT, HiCNN2‐3, HiCPlus and SRHiC. Chr 3, 10, and 19 were used as the test set for mESC, while Chr 3, 10, 20 were used for HFFc6 cell line.

**FIGURE 5:**
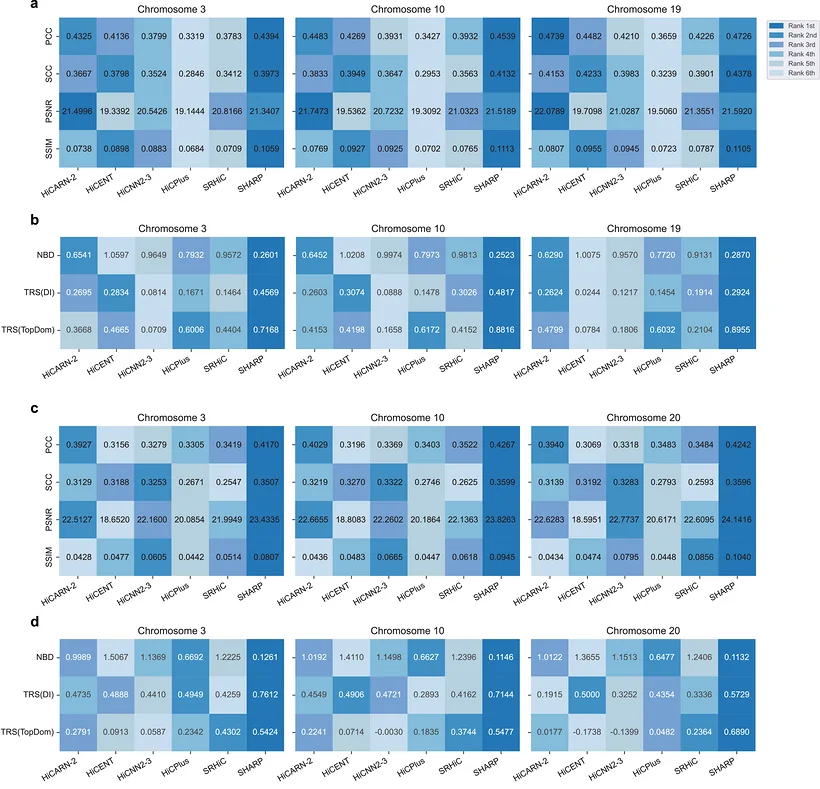
Performance of SHARP as compared to five other methods when applied to a Hi‐C contact matrix of the mESC cell line and a Micro‐C contact matrix of the HFFc6 cell line. (a), Resolution enhancement performance for mESC. (b), Artificial structure avoidance performance for mESC. (c), Resolution enhancement performance for HFFc6. (d), Artificial structure avoidance performance for HFFc6.

By having actual high‐resolution data of the mESC and HFFc6 contact matrix left‐out from the training process, we were able to evaluate the performance objectively. For mESC, in terms of resolution enhancement performance, SHARP ranked first in SCC and SSIM, and shared the top rank with HiCARN‐2 for PCC and PSNR (Figure 5a). We also compared the generalization performance of the methods on pairs of genomic bins at different 1D distances separately (Figure S21). SHARP maintained strong performance at different genomic distances and frequently outperformed other methods. In terms of artificial structure avoidance performance, SHARP consistently outperformed the other five methods in all cases (Figure 5b).

For HFFc6, SHARP consistently achieved the best performance across all evaluation metrics (Figure 5c). Evaluation on pairs of genomic bins at different distances also demonstrates SHARP's superior performance (Figure S22). For HFFc6, SSIM gradually increased with genomic distance for several methods, with the most pronounced increase observed for SHARP. This trend was associated with the increasing sparsity of long‐range Hi‐C contact maps, while SHARP's signal decomposition framework enabled better preservation of the sparse contact distribution. Furthermore, SHARP consistently outperformed other methods in avoiding artificial boundaries (Figure 5d).

### 2.6 Fusing the Resolution‐Enhanced Contact Matrices Produced by SHARP and Other Methods

In computer vision, it has been shown that fusing the outputs of different models is an effective way to improve overall modeling performance since different models can complement each other [42, 43, 44, 45]. To explore this possibility, we developed SHARP‐Fusion, which uses supervised back‐projection [46] to integrate the enhanced Hi‐C contact matrices produced by SHARP and other existing methods (Figure S23a, Methods). The back‐projection procedure projects a base contact matrix onto another matrix, identifies the difference between the latter and the projection, and then adds the back‐projected difference to the base matrix. This is performed multiple times to integrate information contained in the enhanced matrices produced by different existing methods into the enhanced matrix produced by SHARP. The projection is performed using convolutional layers and transposed convolutional layers, the parameters of which are learned using the chromosomes in the training set (Methods).

Comparing SHARP and SHARP‐Fusion, we found that SHARP‐Fusion generally performs better in terms of resolution enhancement accuracy (Figure S23b). However, it creates more artificial structures at the patch boundaries (Figure S23c), likely inherited from the contact matrices produced by the other three methods.

## 3 Discussion

In this study, we have introduced SHARP that accurately enhances the resolution of Hi‐C contact matrices while avoiding the creation of artificial structures at patch boundaries commonly created by other methods. Our main methodological innovations include 1) decomposition of the Hi‐C contact matrices into three types of signals (due to 1D distance, contiguous domains, and other intricate structures, respectively) and enhance them separately, and 2) use of the both local and global attention mechanisms for enhancing the third type of signals. Using actual high‐resolution Hi‐C data of left‐out chromosomes not involved in model training, we have shown that SHARP outperformed five other state‐of‐the‐art methods in terms of both resolution enhancement performance and avoidance of creating artificial structures. We have also shown that these results are robust against 1) different ways to produce the low‐resolution matrix (actual experimental data vs. down‐sampling of high‐resolution data), 2) different down‐sampling rates, and 3) data from different samples (training and testing both on GM12878 or both on h1‐hESC) or another species (training on GM12878 and testing on mESC) or across experimental protocols (training on Hi‐C and testing on Micro‐C).

We have also performed an ablation study to show that the signal decomposition stage of SHARP is useful for avoiding the creation of artificial structures. At the same time, we have shown that the combined use of local and global attention is sufficient to improve resolution enhancement performance over existing methods even without signal decomposition.

In terms of computational resources required, SHARP required relatively higher CPU time (second among all methods) but was on par with the other methods in terms of GPU time and memory usage (Table S1). Among the methods compared, HiCPlus had the lowest computational requirements but its resolution enhancement performance was usually lowest in our computational experiments.

Comparing SHARP and SHARP‐Fusion, SHARP tends to create less artifacts at the patch boundaries while SHARP‐Fusion tends to achieve better overall resolution enhancement performance. This suggests that the fusion procedure integrates some useful signals from the contact matrices produced by the other three methods that are missed by SHARP, but at the same time artificial structures in them are inadvertently also carried over. To combine the advantages of SHARP and SHARP‐Fusion, one potential way is to use SHARP‐Fusion for resolution enhancement but for all significant chromatin interactions and chromatin domains called, use the results of SHARP to confirm or reject them. Alternatively, the loss function and the overall model training procedure could be modified to take into account both enhancement performance and smoothness at patch boundaries.

Thanks to its strong performance in resolution enhancement, SHARP can benefit a broad range of biological applications that rely on high‐quality chromatin interaction maps. For example, low‐input Hi‐C experiments, which often suffer from sparse contact signals due to limited sequencing depth or sample availability, can leverage SHARP's ability to improve signal recovery and facilitate the detection of fine‐scale chromatin structures. In addition, SHARP also facilitates comparative analyzes across different cell types and species by improving the quality of low‐resolution Hi‐C maps, particularly in scenarios where generating high‐depth Hi‐C data for all conditions is challenging. Furthermore, enhanced Hi‐C maps generated by SHARP will strengthen downstream analyzes, including the identification and characterization of TADs and significant chromatin interactions, such as loops. Ultimately, these applications will help provide a more comprehensive understanding of genome organization and its relationship with gene regulation.

While our study evaluated the enrichment of the identified chromatin interactions across different chromatin states, we acknowledge that the lack of extensive enrichment analyzes against other specific functional datasets (e.g., ChIP‐seq, ATAC‐seq, or eQTLs) is a limitation. Incorporating such broader multi‐omic analyzes remains a key direction for future work to further comprehensively validate the biological relevance of our findings.

Extending SHARP to clinical Hi‐C datasets is a critical future direction. However, these datasets typically lack matched high‐resolution ground truth, making objective benchmarking challenging and necessitating extensive wet‐lab validation. Furthermore, many disease genomes (particularly in cancer) harbor massive structural variations (SVs). Because current enhancement models are intrinsically SV‐naive, applying them directly risks erroneously reconstructing underlying SVs computationally as false regulatory loops. Therefore, future algorithmic development must integrate enhancement frameworks with specialized SV callers (e.g., EagleC [47] and NeoLoopFinder [48]). Creating such SV‐aware joint models is essential to safely uncover disease‐specific chromatin interactions without introducing computational artifacts.

In addition, current approaches, including SHARP, primarily rely on the contact matrix itself and do not explicitly incorporate underlying biological determinants such as DNA sequence context or epigenomic features. One advantage of this setting is that no additional data types are required. However, these biological factors are known to influence chromatin organization and may provide complementary information for improving reconstruction accuracy and biological interpretability. Recent studies have begun to explore such integrative strategies, suggesting that combining Hi‐C data with epigenomic signals can further improve tasks such as TAD boundary detection (e.g., EpiTADformer [49]). Building on this, incorporating these additional modalities to enhance Hi‐C resolution represents a promising direction for future development of SHARP and related methods.

## 4 Methods

### 4.1 Details of the SHARP Method

We model a Hi‐C contact matrix by the summation of three types of signals, namely signals due to 1D proximity, signals due to within‐domain and between‐domain contacts of contiguous domains (such as TADs), and signals due to other fine structures. Here we describe how we isolate these three types of signals from a low‐resolution matrix (Algorithm 1), enhance them separately, and sum them back to form a high‐resolution matrix.

ALGORITHM 1 **SHARP Decomposition Pipeline.**

**Table:** 

**Require**: $M\in R^{N\times N}$ (low‐resolution Hi‐C matrix), bin size b, blacklist bins B, parameters Θ	
**Ensure**: Residual matrix R	
1:	**Step 1: Subtract 1D proximity signal**
2:	S←∑M
3:	$k_{\text{init}}\leftarrow 7.0\times S^{0.76}$
4:	k← fine‐tuned value around $k_{\text{init}}$
5:	**for** each pair (i,j) **do**
6:	d←|i−j|×b
7:	**if** d>0 **then**
8:	$P\left[i,j\right]\leftarrow k\cdot d^{-3/2}\cdot \exp\left(-1400/d^{2}\right)$
9:	**else**
10:	continue
11:	**end if**
12:	**end for**
13:	M←M−P
14:	M←max(M,0)
15:	**Step 2: Remove blacklist bins**
16:	Remove all bins in B from M (and re‐index matrix)
17:	**Step 3: Detect and subtract on‐diagonal rectangles**
18:	$R_{\text{on}}\leftarrow$ Detect OnDiagonal Rectangles(M, Θ)
19:	Sort $R_{\text{on}}$ by size (ascending)
20:	**for** each rectangle $r\in R_{\text{on}}$ **do**
21:	Compute: $\mu_{\text{upper}}=$ mean of upper triangle of r
22:	$\mu_{\text{lower}}=$ mean of lower triangle of r
23:	**if** $\mu_{\text{upper}}>1.5\times \mu_{\text{lower}}$ **then**
24:	category ← upper‐triangle
25:	strong region ← upper triangle of r
26:	baseline region ← lower triangle of r
27:	**else if** $\mu_{\text{lower}}>1.5\times \mu_{\text{upper}}$ **then**
28:	category ← lower‐triangle
29:	strong region ← lower triangle of r
30:	baseline region ← upper triangle of r
31:	**else**
32:	category ← uniform
33:	strong region ← all entries in r
34:	baseline region ← immediate outside of r
35:	**end if**
36:	Compute: μ= mean(strong region), $\mu^{\prime }=$ mean(baseline region)
37:	$m\leftarrow (\mu -\mu^{\prime })/\mu$
38:	**for** each x in strong region **do**
39:	x←x−m·x
40:	**end for**
41:	**if** category = upper‐triangle or lower‐triangle **then**
42:	strong region ← all entries in r
43:	baseline region ← immediate outside of r
44:	Compute: $\mu^{\left(2\right)}=$ mean(strong region), $\mu^{\prime (2)}=$ mean(baseline region)
45:	$m^{\left(2\right)}\leftarrow \left(\mu^{\left(2\right)}-\mu^{\prime (2)}\right)/\mu^{\left(2\right)}$
46:	**for** each x in r **do**
47:	$x\leftarrow x-m^{\left(2\right)}\cdot x$
48:	**end for**
49:	**end if**
50:	**end for**
51:	**Step 4: Detect and subtract off‐diagonal rectangles**
52:	**while** an off‐diagonal rectangle can still be identified **do**
53:	Detect an off‐diagonal rectangle r from M
54:	Compute μ (inside r)
55:	Compute $\mu^{\prime }$ (immediate outside of r)
56:	$m\leftarrow (\mu -\mu^{\prime })/\mu$
57:	**for** each x in r **do**
58:	x←x−m·x
59:	**end for**
60:	**end while**
61:	**Step 5: Restore blacklist bins**
62:	Re‐insert bins in B into M at original positions (fill with zeros)
63:	**Step 6: Output**
64:	R←M
65:	**return** R

#### 4.1.1 Modeling Signals Due to 1D Proximity

Contacts due to one‐dimensional genomic proximity are conceptually largely independent of the higher‐order three‐dimensional genome architecture. Genomic regions that are close in linear genomic distance are necessarily spatially close and therefore tend to exhibit high contact frequencies in Hi‐C data, as reflected by the strong signals near the matrix diagonal. As proposed previously [27], this distance‐dependent contact decay can be modeled by the following formula:

(1) $$f\left(d\right)=k\times d^{-3/2}\times e^{-1,400/d^{2}},$$

where d denotes the genomic distance between two loci, and k is a proportionality constant that reflects the efficiency of the cross‐linking reaction.

When applying this formula to model contact frequencies in a Hi‐C matrix, we discretized all genomic distances to multiples of the bin size.

We tested the goodness‐of‐fit of different functional classes for determining a suitable value of k. We found that the formula $k=7.0\times S^{0.76}$ provides a good starting point, where S is the sum of the whole matrix. We then fine‐tuned k by testing different values around this number and finding the value that provided the best balance between removing signals on the diagonal and preserving signals in rectangles, aided by the Hi‐C data visualization tool Juicebox [50]. Using this procedure, we found $k=3.5\times 10^{6}$ worked best for the low‐resolution GM12878 matrix at subsampling rate of 1/150 (Figure S24) and $k=4\times 10^{7}$ worked best for the high‐resolution GM12878 matrix (Figure S25).

To further assess the robustness of this parameter, we conducted a sensitivity analysis by perturbing k by ±20% around its selected value. We observed that most of the metrics, including PCC, SCC, PSNR, SSIM and NBD showed only minor variations (Figures S26–S30), indicating that moderate deviations from the selected k value have limited effects on overall reconstruction quality. In contrast, TAD‐related metrics exhibited relatively larger fluctuations (Figures S31 and S32) because they are based on downstream TAD identification and matching. Small changes in local interaction patterns may shift the identified TAD boundaries, thereby altering TAD matching results and overlap ratios used for TRS calculation. Nevertheless, SHARP consistently achieved higher TRS values than competing methods under the tested perturbations. These results indicate that the empirical formula provides a robust guideline for selecting k, while moderate deviations from the selected value do not substantially affect the overall performance or comparative advantage of SHARP.

#### 4.1.2 Modeling Signals Due to Contiguous Domains

After subtracting out signals due to 1D proximity from the contact matrix, SHARP next identifies and subtracts out signals due to contiguous domains. These signals appear as rectangles that contain larger contact frequencies than surrounding areas (Figure S33). These rectangles can take the form of either squares that appear along the matrix diagonal, which contain interactions between different loci within the same domain, or off‐diagonal stripes [51], which contain interactions between loci from two different domains.

To cope with potentially very complex genome structures, we allow these rectangles to be nested and overlapping. Effects of overlapping rectangles sum together to produce the overall signals due to contiguous domains.

The detection of these rectangles somewhat resembles the task of TAD calling. While some current methods can identify hierarchical TAD structures or work on low‐resolution data [52, 53], they are insufficient for our purpose due to our need for detecting off‐diagonal structures and modeling effects of rectangles on contact frequencies. Therefore, we developed our own procedure for detecting the rectangular blocks and modeling their effects. Here we first give a high‐level description of the procedure, and then provide further details.

##### 4.1.2.1 Overview of the procedure

Our procedure detects and subtracts out the rectangles in the following order:

1. Detect all on‐diagonal rectangles.
2. Subtract out all on‐diagonal rectangles.
3. Detect and subtract out an off‐diagonal rectangle.
4. Repeat #3 until no more off‐diagonal rectangles can be detected.

On‐diagonal and off‐diagonal rectangles are handled differently because on‐diagonal rectangles overlap with each other much more frequently than off‐diagonal rectangles. Therefore, if an on‐diagonal rectangle is subtracted out immediately after its detection before knowing the presence of other overlapping rectangles, its effects on contact frequencies may not be modeled accurately. In contrast, since off‐diagonal rectangles seldom overlap with each other, they can be detected and subtracted out independently using a simpler procedure. The nested detection and subtraction of rectangles enable SHARP to compensate for potentially misidentified patch boundaries.

To prevent our procedure from being misled by problematic regions that possibly contain spurious signals, we excluded all bins (and moved the following bins up) that overlap the low mappability regions and high signal regions on the ENCODE blacklist [54], obtained from https://github.com/Boyle‐Lab/Blacklist/blob/master/lists/hg38‐blacklist.v2.bed.gz.

##### 4.1.2.2 Detecting on‐diagonal rectangles

The procedure for detecting on‐diagonal rectangles is shown in Figure S34. Each on‐diagonal rectangle is a square fully specified by its starting and ending bins. SHARP searches for on‐diagonal rectangles by considering each bin as a potential starting bin in turn. From a potential starting bin, SHARP defines a thin stripe as the potential upper edge of the rectangle. The average contact frequency within the stripe is compared to that of an area of the same length immediately outside the tentative rectangle, which serves as reference. If the former has a larger average frequency, the tentative rectangle will be recorded and further refined in the next stage. To detect rectangles of different sizes, several stripe lengths are tested for each potential starting bin.

For each of these tentative rectangles, SHARP then attempts to expand it by comparing the average contact frequencies within the rectangle, the expansion, and the area immediately outside. The expansion process is then repeated iteratively until the next expansion would not increase the average contact frequency of the whole rectangle.

Details of the whole procedure is given in Algorithm 2.

ALGORITHM 2 **Detecting on‐diagonal rectangles.**

**Table:** 

1:	▹ Variables and their default values
2:	el←10 ▹ Tentative edge length
3:	et←3 ▹ Edge thickness
4:	rt←10 ▹ Outside reference area's thickness
5:	li←10 ▹ Edge length increment during refinement
6:	lm←400 ▹ Edge length maximum
7:	thr←1.3 ▹ A threshold for deciding whether to expand rectangle further
8:	
9:	▹ Find tentative upper edges of all rectangles
10:	**function** findedges(X) ▹ X is the contact frequency matrix
11:	**for all** b **do** ▹ b is the potential starting bin
12:	**for** mul:[1,2,3] **do** ▹ Trying 3 edge length multipliers
13:	edge←X[b:b+et,b:b+el×mul] ▹ edge is the potential upper edge
14:	ref←X[b:b−rt,b:b+el×mul] ▹ ref is the outside reference area
15:	**if** avg(edge) > avg(ref) **then** ▹ Found a promising edge
16:	findrectangle(X,b,el×mul)
17:	**end if**
18:	**end for**
19:	**end for**
20:	**end Function**
21:	
22:	▹ Find the whole rectangle based on the input starting bin and tentative edge length
23:	**function** findrectangle(X,b,l)
24:	cl←l ▹ cl is current edge length
25:	**while** cl<lm **do**
26:	cur←X[b:b+cl,b:b+cl] ▹ cur is current rectangle
27:	ref←X[b:b+cl,b−rt:b] ▹ ref is the outside reference area
28:	exp←X[b:b+cl,b+cl:b+cl+li] ▹ exp is part of potential expansion
29:	fur←X[b:b+cl,b+cl+li:b+cl+li×2] ▹ fur is a further area
30:	
31:	▹ Return current rectangle if expansion has weak signals or the next expansion would lead to too much signal degradation
32:	**if** avg(exp) < avg(ref) OR avg(cur) < avg(fur) ×thr **then**
33:	**return** cur
34:	**end if**
35:	cl←cl+li ▹ Repeat to try expanding more
36:	**end while**
37:	**return** cur ▹ Return the rectangle if maximum size has reached
38:	**end Function**

##### 4.1.2.3 Categorizing on‐diagonal rectangles

While a large proportion of contact frequencies within an on‐diagonal rectangle are larger than those in the immediate surrounding areas, there are three different patterns commonly observed, namely fairly uniform signals in the whole rectangle (Figure S35a), stronger signals in the lower triangle (Figure S35b), and stronger signals in the upper triangle (Figure S35c). The first category includes domains in which most loci can be freely in contact with each other, while the second and third categories include domains that have loci close to one domain boundary more flexibly interacting with other loci in the domain than those close to the other domain boundary.

Based on this observation, for each on‐diagonal rectangle detected, SHARP puts it into:

- The upper triangle category if the average contact frequency in the upper triangle is >1.5 times the average in the lower triangle
- The lower triangle category if the average contact frequency in the lower triangle is >1.5 times the average in the upper triangle
- The uniform signal category otherwise

##### 4.1.2.4 Modeling and subtracting out effects of on‐diagonal rectangles

For all three categories of on‐diagonal rectangles, SHARP models its effects by a constant multiplying factor m that is obtained by comparing strong values within the rectangle with some baseline values. Where the strong values and baseline values are obtained differs for the three categories (Figure S36):

- For the uniform signal category, we take contact frequencies in the whole rectangle as the strong values and contact frequencies in the immediate outside areas as the baseline values.
- For the lower triangle category, we take contact frequencies in the lower triangle as the strong values and contact frequencies in the upper triangle as the baseline values.
- For the upper triangle category, we take contact frequencies in the upper triangle as the strong values and contact frequencies in the lower triangle as the baseline values.

Let μ and $\mu^{\prime }$ be the average of the strong values and the average of the baseline values, respectively. The multiplying factor m is computed as:

(2) $$m=\frac{\mu -\mu^{\prime }}{\mu }$$

To subtract out the signals due to the rectangle, each contact frequency x within the “strong value” area is adjusted using the multiplying factor:

(3) x:=x−m×x

In other words, each original contact frequency in the rectangle is $\frac{1}{1-m}$ times of the adjusted value.

After this adjustment, for each on‐diagonal rectangle in the uniform signal category, the effect of the whole rectangle is expected to be subtracted out such that contact frequencies inside and immediately outside the rectangle should be similar. On the other hand, for the other two categories, the procedure only turns the rectangle into one that has uniform signals but it may still have stronger signals than the immediate outside areas. Therefore, for each rectangle originally in these categories, we apply this adjustment one more time but treating it as in the uniform signal category during this second round of adjustment.

Since different rectangles can be nested or overlapping, SHARP performs the above operations on small rectangles first before large rectangles, by ordering all rectangles by their sizes. By doing so, if a small rectangle is within a big nested rectangle, the baseline values taken immediately outside the small rectangle will likely be still within the big rectangle. As a result, when the small rectangle is processed, only the effect due to it will be adjusted while the effect due to the big rectangle will remain, which will be subtracted out when it is the big rectangle's turn to be processed.

##### 4.1.2.5 Detecting off‐diagonal rectangles and modeling and subtracting out their effects

Off‐diagonal rectangles are detected in a way similar to on‐diagonal rectangles, except that 1) they can be anywhere in the matrix rather than only along the diagonal and 2) they are not necessarily squares. Therefore, each off‐diagonal rectangle needs to be specified by two pairs of starting and ending bins instead of just one pair. During the searching process, when the upper edge of a potential rectangle is identified, both the height and width of the rectangle need to be determined by applying the same procedure to them separately.

When the whole rectangle is defined, its effect on contact frequencies is modeled and subtracted out using the same way as an on‐diagonal rectangle that belongs to the uniform signal category.

##### 4.1.2.6 Sensitivity test

We performed a sensitivity analysis for the parameters used in the rectangle detection algorithm (Figures S26–S32). We observed that performance remained largely stable across a broad range of parameter settings. Importantly, across most of the settings, SHARP's relative performance compared to competing methods remained unchanged, consistently outperforming them in terms of SCC, NBD, and TRS (TopDom).

#### 4.1.3 Modeling Other Signals

After signals due to 1D proximity and contiguous domains have been subtracted out from the contact matrix, SHARP next models relationships between the remaining signals in the low‐resolution and high‐resolution matrices using deep learning. These residual signals, such as chromatin loops, are typically sparse, localized, and low in intensity. Since the first two decomposition steps are not patch‐based, the resulting residuals are not expected to be systematically enriched at patch boundaries, which further reduces the likelihood of introducing strong discontinuities at patch boundaries. Therefore, we use the standard strategy of dividing up the contact matrix into regular patches.

We chose MAXIM [28] as the model architecture, which uses the attention mechanism of the highly successful transformers [55, 56]. Overcoming limitations of full self‐attention [57, 58], which can only be applied to small patches, and local attention [59, 60], which leads to a small receptive field, MAXIM combines both local and global attention.

MAXIM achieves this using a special encoder‐decoder architecture (Figure S37a). A conventional autoencoder aims at reconstructing the input from the embedding captured by the bottleneck layer. MAXIM modifies this objective by considering both the original input and down‐sampled versions of it, which ignore some details but instead focus on higher‐level information. Each version of the data has its own encoder‐decoder pair, but outputs of the different encoders are integrated using cross‐gating blocks (GCBs), thereby allowing features from the different versions of the data to jointly reconstruct each version of it.

Each encoder, decoder, and bottleneck block is composed of layers of multi‐axis gated multi‐layer perceptron (MLP) and residual channel attention blocks [61, 62] (Figure S37b). The multi‐axis gated MLP block performs both regional and dilated attention. Regional attention captures local information more effectively and follows the principles of convolutional operations, while dilated attention is primarily used to fuse long‐range features (i.e., global feature fusion) and is akin to dilated convolutional operations. The residual channel attention block adaptively recalibrates channel‐wise features to emphasize on the most crucial information.

The CGBs were designed to enhance feature propagation in skip‐connections by selectively gating features through contextual information (Figure S37c). At the core of the CGB is the multi‐axis cross‐gating mechanism, which conditions one feature map on another by applying regional and dilated attention, akin to the Multi‐axis Gated MLP block. Three CGBs are employed in the overall MAXIM architecture (Figure S37a), each corresponding to a different input scale to capture contextual interactions at various stages of feature extraction and reconstruction.

The MAXIM backbone is a highly non‐linear model, allowing SHARP to further capture complex relationships between low‐ and high‐resolution signals that may not be fully modeled by the earlier linear decomposition steps.

In our adoption of MAXIM, we use Mean Absolute Error (MAE) [63] as the loss function since it is relatively robust to outliers and does not penalize large error terms as strongly as some other loss functions such as the Mean Squared Error (MSE). In Hi‐C contact matrices, there are occasionally some extremely large values, and it is important to prevent the training process from focusing on modeling these values.

To train the MAXIM model, we used the Adam optimizer [64] with an initial learning rate of $2\times 10^{-4}$. This learning rate was gradually decreased to $10^{-7}$ using cosine annealing decay.

#### 4.1.4 Error Propagation Across Different Stages

SHARP decomposes signals into contacts due to one‐dimensional proximity, contacts due to contiguous domains, and contacts due to other fine‐scale structures, and models them sequentially. Artificial boundaries may be introduced if the modeling is not accurate, for example, if the detected rectangles do not accurately match the true contiguous domains. To mitigate such error accumulation, SHARP incorporates several design choices.

The decomposition of one‐dimensional proximity effects primarily targets strong diagonal signals. Given that this step is based on well‐established models, the associated errors are expected to be limited and unlikely to create artificial boundaries (Figures S24 and S25).

During off‐diagonal rectangle detection, each identified rectangle is subtracted before detecting subsequent ones. This iterative subtraction helps reduce error propagation, as slight inaccuracies in rectangle boundary localization typically leave strong interaction signals in the residual matrix. These remaining signals can still be detected and compensated by subsequent rounds of off‐diagonal rectangle detection, making this type of error generally recoverable and therefore having little impact on the final reconstruction. An illustrative example from an actual SHARP reconstruction on the 1/150 downsampled dataset (Figure S38) shows strong interaction signals remaining after an imperfect rectangle detection are successfully removed in later iterations and are accurately reconstructed in the final enhanced Hi‐C map.

In the final stage, the MAXIM backbone introduces a highly non‐linear refinement step to further correct decomposition errors that remain after the preceding stages. Such errors mainly arise when interaction patterns are too weak to be detected under low‐signal conditions. Trained on paired low‐ and high‐resolution Hi‐C patches, MAXIM can infer part of the missing fine‐scale structures from surrounding interaction patterns and the learned low‐to‐high‐resolution mapping. Because the missing information is no longer explicitly available, this recovery is often only partial, and the corresponding decomposition errors may therefore propagate to the final reconstruction. An illustrative example from an actual SHARP reconstruction on the 1/150 downsampled dataset is provided (Figure S39), where one subdomain is successfully recovered whereas another weaker subdomain is only partially reconstructed, demonstrating both the capability and the limitation of the refinement stage. Moreover, because the remaining fine‐scale residual signals, such as chromatin loops, are typically sparse and localized, they can still be effectively recovered even when overlapping with patch boundaries containing artificial structures.

#### 4.1.5 The Complete Training Process

The overall model training process of SHARP requires paired low‐resolution and high‐resolution Hi‐C contact matrices of the same sample (Figure 1g).

First, signals due to 1D proximity are subtracted out from both the low‐resolution and high‐resolution matrices independently.

Next, from the resulting low‐resolution matrix, on‐diagonal and off‐diagonal rectangles are detected, modeled, and subtracted out. For each rectangle, three types of parameters specific to it are recorded, namely 1) its starting and ending bins (one pair for an on‐diagonal rectangle, two pairs for an off‐diagonal rectangle) 2) its rectangle category, and 3) multiplying factor m. These results are then applied to the high‐resolution matrix to subtract out signals due to contiguous domains, using the parameters learned from the low‐resolution matrix directly.

Finally, taking the remaining signals from the low‐resolution and high‐resolution matrices together, the MAXIM model is trained to minimize the error in enhancing the low‐resolution one to become the high‐resolution one.

#### 4.1.6 Applying SHARP to Enhance a Low‐Resolution Hi‐C Contact Matrix

In the actual use of SHARP, it is given only a low‐resolution Hi‐C contact matrix as input and is tasked to reconstruct a high‐resolution of it using the model previously learned from another sample. The procedure involves several steps:

1. Signals due to 1D proximity are subtracted out from the low‐resolution matrix.
2. Signals due to contiguous domains are detected, modeled, and subtracted out from the low‐resolution matrix.
3. The previously learned MAXIM model is applied to the resulting low‐resolution matrix to reconstruct the high‐resolution matrix without the first two types of signals.
4. Signals due to contiguous domains are added to the reconstructed high‐resolution matrix using the same parameters obtained in Step 2.
5. Signals due to 1D proximity are added to the reconstructed high‐resolution matrix using a proportionality constant obtained from the high‐resolution matrix involved in the original model training of SHARP.

In the reconstructed high‐resolution matrix, if there are any negative values, they are all set to zero. Also, for values larger than the maximum value of the low‐resolution contact matrix multiplied by a threshold, we set them to the median of their surrounding four values. The default value of this threshold is the ratio of total contact frequency of the high‐resolution matrix to that of the low‐resolution matrix of the training data. This clipping step is introduced as a precautionary measure to handle rare extreme values that may arise during reconstruction. To assess its impact, we performed a sensitivity analysis by comparing results with and without clipping (“SHARP‐noclipping”). The results show that this operation had a negligible effect on all evaluation metrics (Figure S40), indicating that it does not materially influence the performance of SHARP but serves as a conservative safeguard.

### 4.2 Other Hi‐C Resolution Enhancement Methods Compared

To assess the performance of SHARP, we compared it with five state‐of‐the‐art Hi‐C resolution enhancement methods, HiCARN [19], HiCENT [32], HiCNN2 [25], HiCPlus [8], and SRHiC [33].

In the original publications of HiCARN and HiCNN2, multiple variants of these models are described. We chose the best‐performing ones based on the results presented in the original publications, namely HiCARN‐2 for HiCARN and HiCNN2‐3 for HiCNN2.

When applying these methods, we follow the parameter settings recommended in the original publications, including learning rate, optimizer, and other hyperparameters.

To ensure a fair comparison of boundary effects across methods, we standardized the output patch size to 64×64, consistent with the design of SHARP, and avoided any overlap between output patches during both training and inference. To achieve this, for methods that preserved the input patch size (HiCENT and HiCARN‐2), we directly partitioned the matrices into non‐overlapping patches of size 64×64; For methods that produced output regions smaller than the input patches (HiCNN2‐3, HiCPlus, and SRHiC), we used input patches of size 76×76 with an overlap of 6 bins on each side with the next patch, leading to output regions of size 64×64. For every method, the reconstructed patches were assembled into the full matrix without overlap.

All reconstructed contact matrices were visualized using the same visualization pipeline. Specifically, the reconstructed matrices were directly loaded into Juicebox [50] without additional image preprocessing to ensure a fair visual comparison across methods.

### 4.3 Details of the SHARP‐Fusion Method

The back‐projection method [46] used by SHARP‐Fusion was originally developed for image enhancement, with a goal of reconstructing a high‐resolution image by combining multiple low‐resolution images sequentially. In our adoption of the method, we used it to reconstruct a high‐resolution Hi‐C contact matrix by combining multiple reconstructed high‐resolution matrices produced by different methods sequentially (Figure S23a).

Specifically, the output (i.e., reconstructed high‐resolution matrix) of SHARP is used as the base matrix. Information contained in the output of HiCPlus that is useful for reconstructing the high‐resolution matrix but missing in the base matrix is added to it. The same procedure is then repeated to add useful information from the outputs of HiCARN‐2 and HiCNN2‐3 sequentially. Mathematically, this process can be summarized by the following formulas:

(4) $$\begin{array}{ccc} F_{1} & = & O_{\text{SHARP}}+C_{1}^{\prime }\left(C_{1}\left(O_{\text{SHARP}}\right)-O_{\text{HiCPlus}}\right) \end{array}$$

(5) $$\begin{array}{ccc} F_{2} & = & F_{1}+C_{2}^{\prime }\left(C_{2}\left(F_{1}\right)-O_{\text{HiCARN-2}}\right) \end{array}$$

(6) $$\begin{array}{ccc} F_{3} & = & F_{2}+C_{3}^{\prime }\left(C_{3}\left(F_{2}\right)-O_{\text{HiCNN2-3}}\right), \end{array}$$

where $O_{X}$ is the output of method X, $C_{i}$ and $C_{i}^{\prime }$ are convolutional layers for projecting one matrix onto another and projecting the difference back, respectively, in the i‐th sequential fusion stages, and $F_{i}$ is the fusion result of the i‐th stage. The final output, $F_{3}$, is the output of SHARP‐Fusion.

During model training, parameters in the 6 convolutional layers are learned together to minimize reconstruction errors as compared to the actual high‐resolution contact matrix. To train the model, we used the Adam optimizer with a learning rate of $5\times 10^{-4}$.

When using SHARP‐Fusion to enhance a low‐resolution matrix without paired high‐resolution data, the above formulas are applied directly using the learned parameters.

### 4.4 Data Sets Used to Compare the Different Methods

#### 4.4.1 Data Sources

The Hi‐C data used in this study were produced by in situ Hi‐C [7] and Micro‐C [41]. We downloaded the data from the 4D Nucleome Data Portal [2, 65], with experiment set accession numbers 4DNES3JX38V5 (high‐resolution GM12878), 4DNESBJ1KYYH (low‐resolution GM12878), 4DNESX75DD7R (H1‐hESC), 4DNESDXUWBD9 (mESC), and 4DNESWST3UBH (Micro‐C HFFc6).

#### 4.4.2 Resolution and Bin Size

Following the criteria established by Rao et al. [7], resolution is defined as the smallest bin size at which at least 80% of bins contain ≥ 1000 contacts. Under this definition, the high‐resolution GM12878 reference data correspond to 5 kb resolution, whereas the 1/150 downsampled data correspond to approximately 200 kb resolution. It is important to distinguish this data resolution (which reflects sequencing depth) from the modeling bin size (the length of the genomic interval used for computation). To ensure a fair comparison across methods and fully exploit the biological information supported by our reference data, the modeling bin size is fixed at 5 kb for all experimental settings. While our framework can be adapted to larger bin sizes (e.g., 10 kb), using a 5 kb bin size is important for preserving fine‐scale chromatin structures and enabling downstream analyzes such as enhancer‐target gene pair identification, where larger bins may contain multiple enhancers or genes simultaneously.

#### 4.4.3 Data Processing

For each Hi‐C dataset, we converted the data into a symmetric contact matrix at 5 kb bin size using Juicer [66] without applying any normalization. Each matrix entry therefore represents the interaction frequency between a pair of genomic bins. We note that this bin size is fixed for all the settings to ensure fair comparison across methods, and reflects the highest resolution supported by our reference data. While larger bin sizes (e.g., 10 kb) could be used, they would not fully utilize the available resolution, although our model can be readily adapted to other targets if needed.

We used unnormalized Hi‐C matrices as input for two main reasons. First, normalization methods such as KR [67] or ICE [68] can be applied to unnormalized matrices, whereas once a matrix has been normalized, it is not always straightforward to recover the unnormalized version or transform it into another normalized form. Second, different Hi‐C resolution enhancement methods may require different preprocessing strategies, such as scaling the data to different value ranges. By providing unnormalized matrices as input, each method can perform its preferred preprocessing procedure consistently.

We found some outlier values in the Hi‐C data. For example, in the high‐resolution GM12878 data, the largest contact frequency, between two bins on chromosome 16, has a value of 106 901, while in other chromosomes, the largest contact frequency is only around 2000. These outlier values were likely caused by technical errors [69]. To avoid these outlier values from biasing the models, we set them to a more reasonably large value. Specifically, we sorted all contact frequencies in ascending order and identified the first value in the sorted list that is 10 or more larger than its previous value. That previous value was defined as the new maximum and all values after it on the sorted list were set to this maximum.

When applying models trained on GM12878 to enhance low‐resolution matrices from other cell lines (e.g., mESC and HFFc6), an additional normalization step was performed to account for differences in contact value ranges between samples. We analyzed the median values of the diagonals in the low‐resolution Hi‐C matrices and found that these differences were primarily concentrated on the main diagonal and neighboring diagonals, whereas contact frequencies at larger genomic distances exhibited comparable value ranges across samples. Therefore, for each chromosome, we calculated the median contact frequency for the main diagonal and neighboring diagonals separately in the low‐resolution matrices and collected these values across chromosomes. For each diagonal, the overall median contact frequency was used to estimate the relative value difference between the target sample (mESC or HFFc6) and the source sample (GM12878). Scaling factors were then calculated independently for each diagonal and applied only when substantial differences in contact values were observed. As the genomic distance increased, the contact values between samples became comparable; therefore, diagonals at larger genomic distances were left unchanged without additional scaling. All scaling factors were estimated solely from the input low‐resolution Hi‐C matrices and applied before resolution enhancement. One potential limitation of this strategy is that, when substantial copy number variations (CNVs) are present in the genome, such as in cancer samples, diagonal entries may vary considerably across genomic regions. In such cases, the scaling factor may need to be estimated using a strategy that explicitly accounts for CNVs.

All Hi‐C resolution enhancement methods received the same processed data as input.

#### 4.4.4 Down‐sampling for Simulating Low‐resolution Matrices

From an actual high‐resolution Hi‐C contact matrix, we performed down‐sampling to simulate a low‐resolution contact matrix of the same sample. For a down‐sampling rate of r, for each contact in the high‐resolution matrix, we kept it in the simulated low‐resolution matrix with a probability of r. We implemented it by down‐sampling each matrix entry in turn. For a matrix entry originally having a contact count of c in the high‐resolution matrix, we sampled a value from the binomial distribution with mean cr and variance cr(1−r) to become the corresponding contact count in the simulated low‐resolution matrix.

### 4.5 Experimental Settings

#### 4.5.1 Bin Pairs for Model Training, Selection, and Evaluation

To enable unbiased performance evaluation, following previous studies [17, 25], we divided all chromosomes into three disjoint subsets. For human data (GM12878 and H1‐hESC), Chromosomes 1, 2, 4‐9, 11‐15, 18, and 19 were used for model training. Some methods produced different possible models during the training process, such as the different MAXIM models produced by SHARP in different training epochs. The performance of these different models was compared using Chromosomes 16, 17, 21, and 22, and the one with the best performance (quantified by PCC × SCC after confirming they were both positive) was chosen. Finally, the model was evaluated using Chromosomes 3, 10, and 20, which were left‐out from all these previous steps. These three chromosomes serve as representatives of long, medium, and short chromosomes, respectively.

When applying models learned from GM12878 to enhance the mESC contact matrix, mouse Chromosomes 3, 10, and 19 were used for performance evaluations. When applying models learned from GM12878 to enhance the HFFc6 contact matrix, Chromosomes 3, 10, and 20 were used for performance evaluations.

For all the resolution enhancement methods, we used a patch size of 64 bins × 64 bins, i.e., 320 kb × 320 kb. Consistent with established practices in Hi‐C resolution enhancement (e.g., HiCPlus [8], HiCARN‐2 [19] and HiCNN2 [25]), we focused our evaluation on chromatin interactions within a genomic distance of 1.92 Mb. This cutoff is chosen because the vast majority of meaningful regulatory interactions, such as those within TADs, typically occur within a 1‐2 Mb range. Beyond this distance, chromatin contacts become increasingly sparse, and evaluations become increasingly susceptible to experimental noise. These bin pairs will be described as those included in the “evaluation set” below. For the remaining regions in the Hi‐C contact maps, we directly copied the data from the low‐resolution matrix to the inferred high‐resolution matrix.

The small‐patch splitting strategy is necessary because high‐resolution Hi‐C contact matrices are extremely large in practice (e.g., 50, 000 × 50, 000 at 5 kb resolution for human chromosome 1), making end‐to‐end processing computationally challenging in terms of both memory and runtime. For example, with only 32 feature channels, a single activation layer in a deep neural network would require approximately 320 GB of memory for the full matrix alone. Even when restricting the analysis to interactions within a 1.92 Mb genomic distance cutoff, the matrix still needs to be divided into 384 × 384 patches at 5 kb resolution, resulting in approximately 130 patches for chromosome 1. Under this setting, a single activation layer would still require approximately 2.5 GB of memory. Since deep neural networks contain many activation layers and additional intermediate tensors during training, the actual memory usage is substantially higher than this estimate, making full chromosome processing impractical on commonly used GPUs.

#### 4.5.2 Default Setting

In the empirical comparisons, by default the low‐resolution contact matrix used was the one down‐sampled from the corresponding high‐resolution contact matrix at a sampling rate of 1/150 because it matches the resolution of the actual low‐resolution GM12878 matrix. Accordingly, by default the “ground truth” high‐resolution matrix used for performance evaluation was that actual high‐resolution contact matrix.

In all the results reported, except for the changes explicitly stated (e.g., using a different down‐sampling rate or using another sample), all other configurations remain the same as this default setting.

#### 4.5.3 Alternative Settings for Robustness Evaluation

In addition to the default setting, we also compared the performance of SHARP with the five other methods using some alternative settings. These include using other down‐sampling rates (1/50 and 1/100) to simulate a low‐resolution GM12878 contact matrix, using an actual low‐resolution GM12878 contact matrix, and using Hi‐C data from H1‐hESC (with the low‐resolution contact matrix of it produced by down‐sampling at the rate of 1/150). In each of these cases, the paired high‐ and low‐resolution matrices were used to perform training, model selection, and testing, based on the different sets of chromosomes as in the default setting.

#### 4.5.4 Runtime and Memory Usage

We systematically evaluated the runtime and memory usage of SHARP and the baseline methods under consistent experimental conditions. Specifically, all measurements were collected during the first training epoch using the same batch size (100), input patch size (64×64), and hardware platform (RTX 2080Ti) to ensure a fair comparison.

Each method was benchmarked in terms of CPU memory usage, GPU memory usage, CPU runtime and GPU runtime (Table S1). Overall, SHARP exhibited comparable resource requirements to existing methods. While SHARP incurred higher computational cost on the CPU, its GPU runtime remained competitive with the other deep learning‐based approaches. In terms of memory usage, SHARP required slightly more GPU memory than HiCNN2‐3 and HiCPlus, but substantially less than HiCARN‐2. These results indicate that the improved performance of SHARP was achieved with moderate computational overhead.

#### 4.5.5 Ablation Study

To evaluate the significance of signal decomposition, we produced a variation of SHARP (called “SHARP‐NoDecomposition”) that does not subtract out the first two types of signals. Instead, it takes the low‐resolution matrix and directly trains a MAXIM model to enhance it. In addition, to assess the contribution of the MAXIM backbone architecture, we implemented another variant that retains the same signal decomposition strategy but replaces the MAXIM model with a simple 5‐layer convolutional network (denoted as “SHARP‐CNN”).

### 4.6 Evaluation Measures for Resolution Enhancement Performance

We used four standard evaluation measures to quantify the resolution enhancement performance of the methods compared. These measures consider different aspects of the performance and were commonly used in previous studies [17, 19, 29]. For each measure, we computed its value independently for each 64 × 64 Hi‐C patch and reported the average across all patches in the evaluation set. This patch‐level evaluation assesses the overall reconstruction quality within local genomic regions, where contacts spanning multiple genomic distances jointly contribute to each metric.

The first measure is the Pearson correlation coefficient (PCC) [70], which measures the linear correlation between two lists of values of the same length. PCC ranges from –1 to 1, with a larger value corresponding to a more positive linear relationship between the two lists. To use PCC to evaluate resolution enhancement performance, we flattened the reconstructed and actual high‐resolution contact matrices within each patch into two one‐dimensional vectors according to the same genomic ordering, computed the PCC for each patch independently, and finally averaged the PCC values across all patches.

The second measure is the Spearman correlation coefficient (SCC) [71]. It is similar to PCC but instead of considering the contact frequencies directly, SCC considers their positions in the sorted list of contact frequencies. We used the stats.spearmanr function in the SciPy library, which gives all values in tie their average rank in the group. As compared to PCC, SCC emphasizes more on the order of the values than the exact values. As a result, SCC is less affected by outliers than PCC. On the other hand, SCC is more affected than PCC when there is a lot of similar values the order of which is not important.

The third measure is peak signal‐to‐noise ratio (PSNR) [72]. It compares the maximum value and the root mean square error between the reconstructed and actual high‐resolution matrices. A larger value of PSNR indicates better reconstruction performance.

The fourth measure is the structural similarity index measure (SSIM) [73]. It uses spatial dependencies to model structural features and compares these features in the reconstructed and actual high‐resolution matrices. However, the interpretation of SSIM may vary across different genomic distance ranges due to differences in contact sparsity. At shorter genomic distances, where interaction signals are relatively dense, SSIM more directly reflects the preservation of local chromatin interaction patterns. In contrast, at larger genomic distances, where Hi‐C contact maps become increasingly sparse, SSIM can be strongly influenced by the overall distribution of low‐frequency or zero‐valued contacts. Therefore, a higher SSIM at large genomic distances does not necessarily indicate more accurate recovery of individual long‐range chromatin interactions, although it can reflect better preservation of the overall contact‐frequency distribution. SSIM should thus be interpreted together with other correlation‐based and structural evaluation metrics for a comprehensive assessment of reconstruction performance.

### 4.7 Evaluation Measures for Artificial Structure Avoidance Performance

We proposed two measures for quantifying the performance of different resolution enhancement methods in avoiding the creation of artificial structures due to patch boundaries.

The first measure is Normalized Boundary Difference (NBD). Consider the left boundary of a patch i in a reconstructed high‐resolution matrix. Let $x^{\left(i\right)}$, $y^{\left(i\right)}$, and $z^{\left(i\right)}$ be the first column inside the patch, second column inside the patch, and first column outside the patch, respectively (Figure S7). If no artificial structures are created at this boundary, the internal difference between $x^{\left(i\right)}$ and $y^{\left(i\right)}$ should be similar to the cross‐boundary difference between $x^{\left(i\right)}$ and $z^{\left(i\right)}$. Based on this, we defined the following measure considering the left boundaries of all patches together:

(7) $$\begin{array}{ccc} D_{\text{int}} & = & \sum_{i}\sum_{j=1}^{n}\left|x_{j}^{\left(i\right)}-y_{j}^{\left(i\right)}\right| \end{array}$$

(8) $$\begin{array}{ccc} D_{\text{cross}} & = & \sum_{i}\sum_{j=1}^{n}\left|x_{j}^{\left(i\right)}-z_{j}^{\left(i\right)}\right| \end{array}$$

(9) $$\begin{array}{ccc} NBD_{\text{left}} & = & \frac{D_{\text{cross}}-D_{\text{int}}}{D_{\text{int}}} \end{array}$$

We also defined the same measure for the other three boundaries of the patches and used their average as the final performance measure:

(10) $$NBD=\frac{NBD_{\text{left}}+NBD_{\text{right}}+NBD_{\text{top}}+NBD_{\text{bottom}}}{4}$$

NBD is influenced by both genomic distance and sequencing coverage, which are intrinsically correlated in Hi‐C contact maps. Patches close to the main diagonal typically contain stronger interaction signals and therefore higher coverage, whereas distal regions become progressively sparser. Consequently, these two factors exhibit similar effects on NBD.

Near the main diagonal, Hi‐C contact frequencies are strongly governed by the distance‐dependent power‐law decay of chromatin interactions, in which interaction intensity decreases super‐linearly with increasing genomic distance. Therefore, the internal difference near the boundary is expected to be relatively larger than the difference across the boundaries, leading to slightly negative NBD values in patches closest to the diagonal and with the highest coverage.

As distance to the diagonal increases, the distance‐dependent decay becomes less dominant and local structural patterns contribute more prominently to the contact map. In those patches, the difference across the boundaries exceeds the internal difference near the boundary, resulting in moderately positive NBD values.

As genomic distance further increases, the Hi‐C matrix becomes increasingly sparse and noisy, making the cross‐boundary difference and within‐patch differences more similar. Accordingly, NBD values gradually approach zero in these patches.

Although biological structures such as TAD boundaries can locally affect NBD when they overlap with patch boundaries, these events occur irregularly and do not systematically coincide with the patch boundaries. Therefore, the dominant contribution to NBD arises from artificial discontinuities introduced rather than from intrinsic chromatin organization.

We performed a sanity check to verify the properties of NBD with the coverage (Figure S41) and the distance‐to‐diagonal (Figure S42). The values in actual high‐resolution maps were performed explicitly as we expected.

The second measure is the TAD‐related score (TRS). It quantifies incorrect TADs called from a reconstructed high‐resolution matrix caused by the patch boundaries. Roughly speaking, a TAD called from a reconstructed matrix is considered incorrect if it is not also called from the actual matrix. However, there are three difficulties in defining such a quantitative measure. First, if a TAD is considered correct only if there is also a TAD called from the actual high‐resolution matrix with exactly the same starting and ending bins as it, most TADs would be considered incorrect since TAD boundaries can be easily shifted by small changes of the contact matrix. Second, even if the definition of a correct TAD tolerates some shifts of the TAD boundaries, it is still not trivial to determine how TADs called from two matrices should be matched. Third, the number of TADs called can easily bias the results. For example, if from a reconstructed matrix very few TADs are called, the ratio of them with a starting bin or ending bin close to a patch boundary could be small just by chance, but this does not mean the resolution enhancement method used to reconstruct the matrix is unaffected by patch boundaries.

Taking all these into account, our TRS measure first considers the ratio of TADs called from the reconstructed matrix that have a starting bin or ending bin near a patch boundary. A larger ratio means potentially more incorrect TADs caused by the patch boundaries. However, some TADs called from the actual high‐resolution matrix can also have starting or ending bins near patch boundaries. Therefore, our TRS measure further checks to what extent these actual TADs overlap the TADs called from the reconstructed matrix. A larger overlap means more support that the TAD boundaries close to patch boundaries are actually correct. Finally, these two components are combined to become the overall TRS measure.

Specifically, the procedure for computing TRS includes the following steps (more details of individual steps will be provided later):

1. Perform a matching of all the TADs called from the actual high‐resolution contact matrix and all the TADs called from the reconstructed matrix. For each pair of overlapping TADs, an overlap ratio is computed based on their starting and ending bins. If a TAD cannot be matched, its overlapping ratio is 0.
2. Collect all TADs called from the actual high‐resolution matrix that have a starting bin or ending bin near a patch boundary.
3. Compute the average overlap ratio of all the TADs collected in Step 2, defined as the **TAD Recovery Score**.
4. Compute the **Boundary Artifact Score** as
$$\frac{\begin{array}{c} \#\text{TADs called from reconstructed matrix with}\\ \;\text{starting or ending bin near a patch boundary} \end{array}}{\#\text{TADs called from reconstructed matrix}}.$$
  Then, compute the final TRS as
TRS=TADRecoveryScore−BoundaryArtifactScore.

Based on this definition, a larger TRS value indicates a smaller number of incorrect TADs caused by the patch boundaries. If very few TADs are called from the reconstructed matrix, the average overlap ratio would be small since most TADs called from the actual matrix would be unmatched, and therefore the TRS value would not be large in that case.

The TAD matching is performed using a greedy algorithm. For each TAD i called from the actual high‐resolution matrix and each TAD j called from the reconstructed matrix, an overlap ratio is computed as $\frac{1}{2}\left(\frac{l_{ij}}{l_{i}}+\frac{l_{ij}}{l_{j}}\right)$, where $l_{i}$ and $l_{j}$ are the lengths (i.e., number of bins) of TAD i and TAD j, respectively, and $l_{ij}$ is length of their intersection. All these overlap ratios are then sorted in descending order. The two TADs that give the largest overlap ratio are matched, and all overlap ratios of these TADs are removed from the sorted list. This matching process is then repeated until the largest overlap ratio is 0, at which time all remaining TADs are considered unmatched and they all have an overlap ratio of 0.

In Steps 2 and 4, the starting bin or ending bin of a TAD is considered near a patch boundary if they are differed by no more than 2 bins.

Intuitively, a reconstructed Hi‐C matrix achieves the highest TRS when it both minimizes TAD boundaries artificially aligned with patch boundaries and accurately recovers true biological TADs. A sanity check of the TAD Recovery Score and Boundary Artifact Score (Figure S43) shows trends consistent with intuition. Reconstruction methods with fewer artificial boundaries and more accurate TAD recovery obtained higher overall TRS values.

However, if the reconstruction excessively smooths the Hi‐C contact map, resulting in only a small number of detected TADs, TRS can be artificially maximized in cases where no true TAD boundaries are near patch boundaries, and the reconstructed matrix likewise contains no TADs near such boundaries.

### 4.8 Analysis of Significant Interactions

#### 4.8.1 Definition of Significant Interactions

From a (reconstructed or actual) high‐resolution Hi‐C contact matrix, we applied FitHiC2 [36] and HiCCUPS [7] to estimate the statistical significance of the contact frequencies. In FitHiC2, all bin pairs at a genomic distance between 15 kb and 1 Mb with a q‐value less than $1\times 10^{-7}$ were considered to have a significant interaction. We utilized the default parameters specified by HiCCUPs for processing high‐resolution Hi‐C contact maps.

#### 4.8.2 Evaluation Measures for Significant Interactions

We used two common measures to evaluate the concordance between the significant interactions identified from the reconstructed and actual high‐resolution matrices.

The first measure is the F‐1 score. Let R and A be the sets of significant interactions identified from the reconstructed and actual high‐resolution matrices, respectively, and |X| be the number of significant interactions in a set X. Precision computes the proportion of significant interactions identified from the reconstructed matrix that are also supported by the actual matrix; recall computes the proportion of significant interactions identified from the actual matrix that are also identified from the reconstructed matrix; finally the F‐1 score aggregates these two measure by taking their harmony mean:

(11) $$\begin{array}{ccc} \text{Precision} & = & \frac{\left|R\cap A\right|}{\left|R\right|} \end{array}$$

(12) $$\begin{array}{ccc} \text{Recall} & = & \frac{\left|R\cap A\right|}{\left|A\right|} \end{array}$$

(13) $$\begin{array}{ccc} \text{F-1} & = & \frac{2\times \text{Precision}\times \text{Recall}}{\text{Precision}+\text{Recall}} \end{array}$$

The second measure is the Jaccard index. It uses a different way to penalize both wrong and missing significant interactions by considering the number of interactions in the union of the two sets:

(14) $$\begin{array}{ccc} J & = & \frac{\left|R\cap A\right|}{\left|R\cup A\right|} \end{array}$$

Both measures have a range between 0 and 1, with a larger value corresponding to better concordance between the two sets of significant interactions.

#### 4.8.3 Analysis of Chromatin States

We obtained the chromatin states of individual 200bp regions in the GM12878 genome inferred by a ChromHMM [74] 15‐state model lifted to hg38 from https://egg2.wustl.edu/roadmap/data/byFileType/chromhmmSegmentations/ChmmModels/coreMarks/jointModel/final/ produced by the NIH Roadmap Epigenomics Mapping Consortium [75]. For each genomic bin involved in a significant interaction, we recorded the number of base pairs in it that overlap with each chromatin state. By summing these base pair counts over the genomic bins, we computed the fraction of total genomic span of these bins that belongs to each chromatin state. Similarly, we computed the fraction of genomic span that belongs to this chromatin state in the whole genome. The ratio of the former to the latter was defined as the fold enrichment.

To evaluate the consistency of chromatin state enrichment patterns between reconstructed and actual high‐resolution maps, we computed PCC and SCC between their enrichment profiles. Specifically, for each matrix, the fold enrichment values across the 15 chromatin states were arranged into a 15‐dimensional vector. PCC was calculated to measure the linear correspondence between the enrichment values of the reconstructed and actual high‐resolution maps, whereas SCC was calculated based on the rank ordering of the enrichment values across chromatin states. Higher PCC and SCC values indicate better preservation of the chromatin state enrichment patterns.

#### 4.8.4 Reproducibility Analysis Across Replicates

To evaluate the reproducibility of interaction across replicates, we used four replicates of high‐resolution GM12878 Hi‐C data. To avoid the bias introduced by the unbalanced reads, each replicate was subsampled to match the lowest read count among them at each chromosome. The SHARP model was trained using replicate 1, and evaluation was performed on left‐out chromosomes from replicate 1 as well as the corresponding chromosomes from replicates 2‐4.

### 4.9 Genomic Distance‐Stratified Analysis

In addition to the patch‐level evaluation described above, we also performed a genomic distance‐stratified analysis to evaluate reconstruction performance at individual genomic distances.

For each of PCC, SCC, PSNR, and SSIM, and for each genomic distance (i.e., number of genomic bins), we collected all contact frequencies in the reconstructed and actual high‐resolution contact matrices corresponding to bin pairs separated by exactly that distance and computed a single performance value. Thus, unlike the patch‐level evaluation, which combines contacts spanning multiple genomic distances within each local region, this analysis evaluates the agreement of contact frequencies at a specific genomic distance only. For PSNR and SSIM, the input was therefore a one‐dimensional vector of contact frequencies rather than a two‐dimensional Hi‐C patch used in the patch‐level evaluation.

### 4.10 Statistical Analysis

Hi‐C contact maps were generated at 5 kb resolution without additional normalization. To minimize the influence of extreme outlier contact frequencies, unusually large values in the Hi‐C contact matrices were capped using a data‐driven threshold.

For both reconstructed and actual high‐resolution Hi‐C matrices, the statistical significance of chromatin interactions was evaluated using FitHiC2 and HiCCUPS. In FitHiC2, interactions spanning 15 kb to 1 Mb with q‐values below $1\times 10^{-7}$ were considered significant. HiCCUPS was performed using its default settings for high‐resolution Hi‐C data.

## Author Contributions

KYY conceived the study. QL and KYY designed the methods. QL implemented the methods, collected and processed the data, and performed the data analysis. QL, KYL, CN, SKWT, PLP, QC, and KYY interpreted the results. QL, QC, and KYY wrote the manuscript. All authors read and approved the final version of the manuscript.

## Conflicts of Interest

The authors declare no conflicts of interest.

## Supporting information


**Supporting File**: advs77221‐sup‐0001‐SuppMat.pdf.

## Data Availability

All the data that support the findings of this study are openly available on the 4D Nucleome data portal at https://data.4dnucleome.org/.
