# Supplementary material for: Overcoming Artificial Structures in Resolution‐Enhanced Hi‐C Data by Signal Decomposition and Multi‐Scale Attention
Source: Adv Sci (Weinh). 2026 Sep 8:e24180. Online ahead of print. doi: 10.1002/advs.202524180 (PMC13554405; doi:10.1002/advs.202524180)
Supplement: Supplementary file 1 [file ADVS-9999-e24180-s001.pdf]

Supplementary information

Supplementary Tables

Supplementary Table S1: Comparison of computational resources required by the different methods for one batch of training in the first epoch.

Method	CPU Memory (MB)	GPU Memory (MB)	CPU Time (ms)	GPU Time (s)
HiCARN-2	2322.85	666.85	788.17	2.62
HiCENT	5749.00	13.28	1819.50	73.90
HiCNN2-3	2290.74	47.18	773.32	3.03
HiCPlus	2259.38	1.46	387.80	1.53
SRHiC	2854.89	5.39	223.02	13.599
SHARP	2370.04	77.61	1290.00	3.01

Supplementary Figures

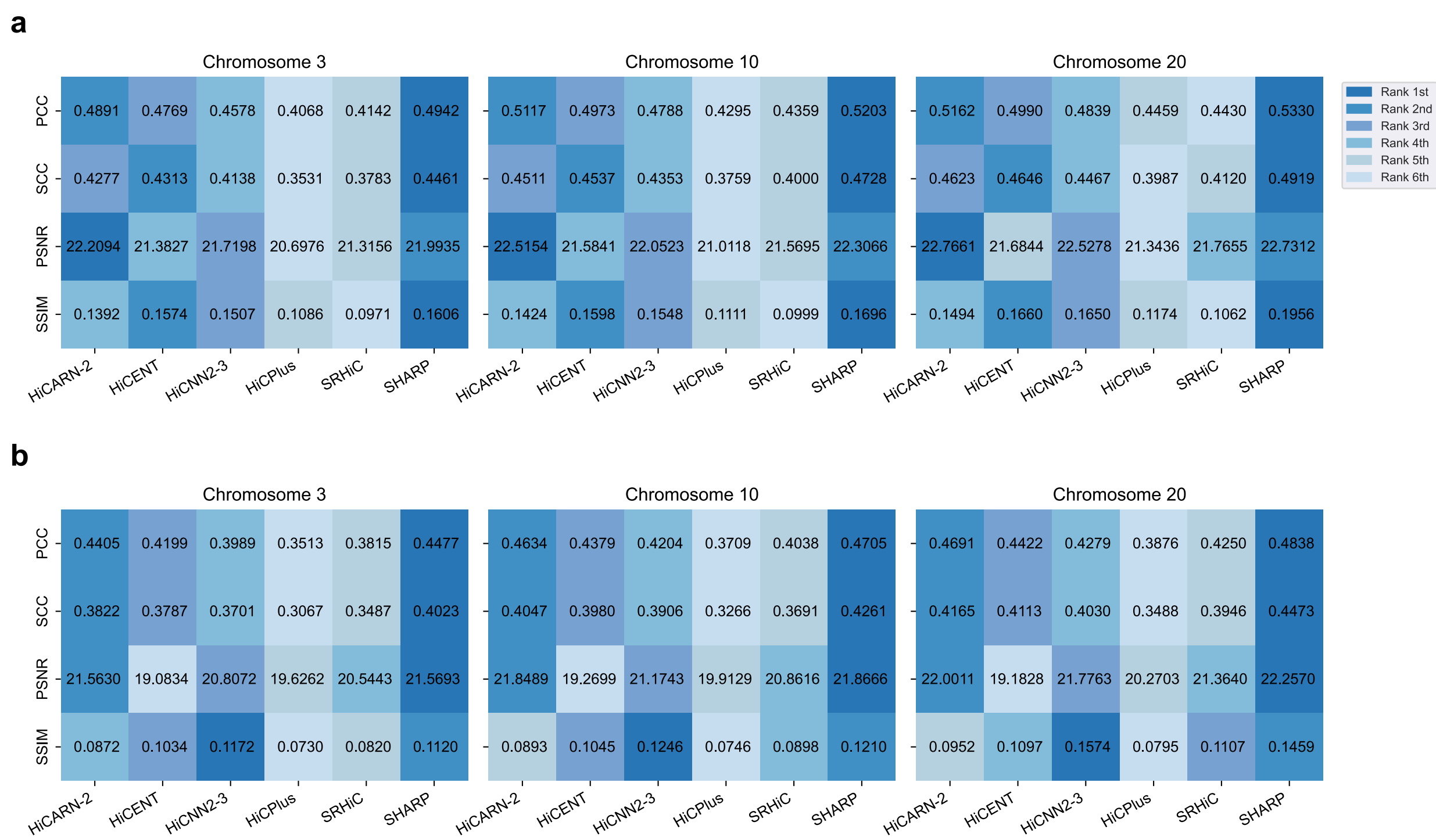

Supplementary Figure S1: Resolution enhancement performance of SHARP as compared to five other methods when applied to low-resolution contact matrices down-sampled from a high-resolution matrix at different sampling rates. a, Sampling rate of 1/50. **b,** Sampling rate of 1/100.

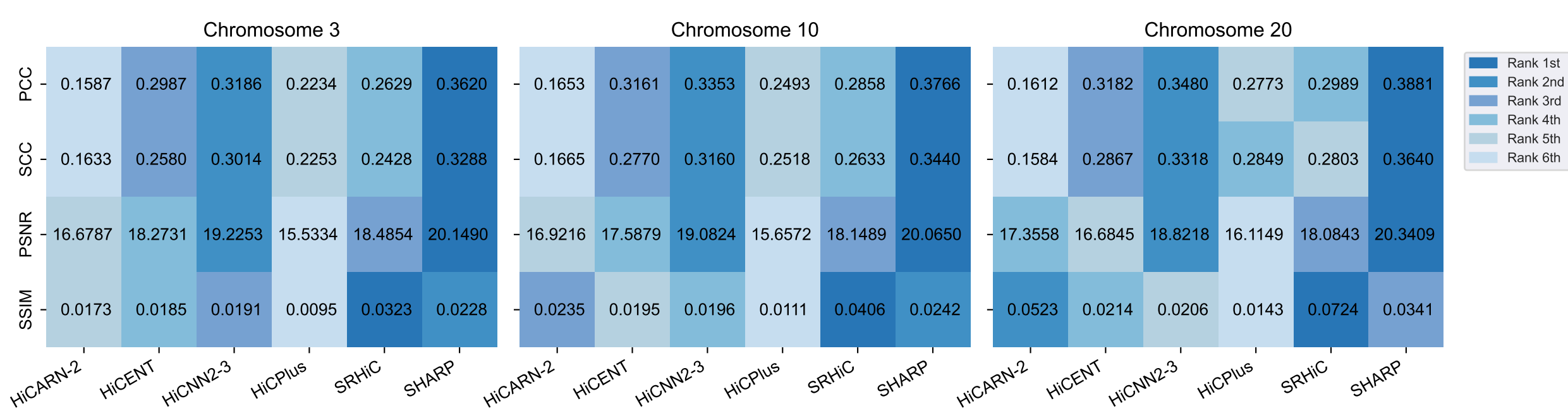

Supplementary Figure S2: Resolution enhancement performance of SHARP as compared to five other methods when applied to an actual low-resolution contact matrix.

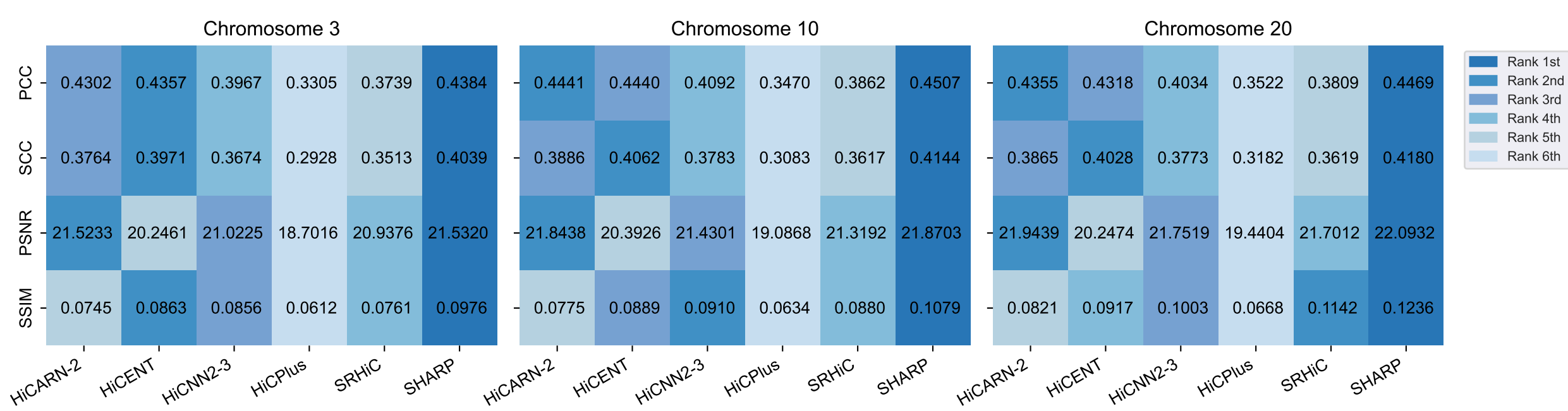

Supplementary Figure S3: Resolution enhancement performance of SHARP as compared to five other methods when applied to a Hi-C contact matrix of the H1-hESC cell line.

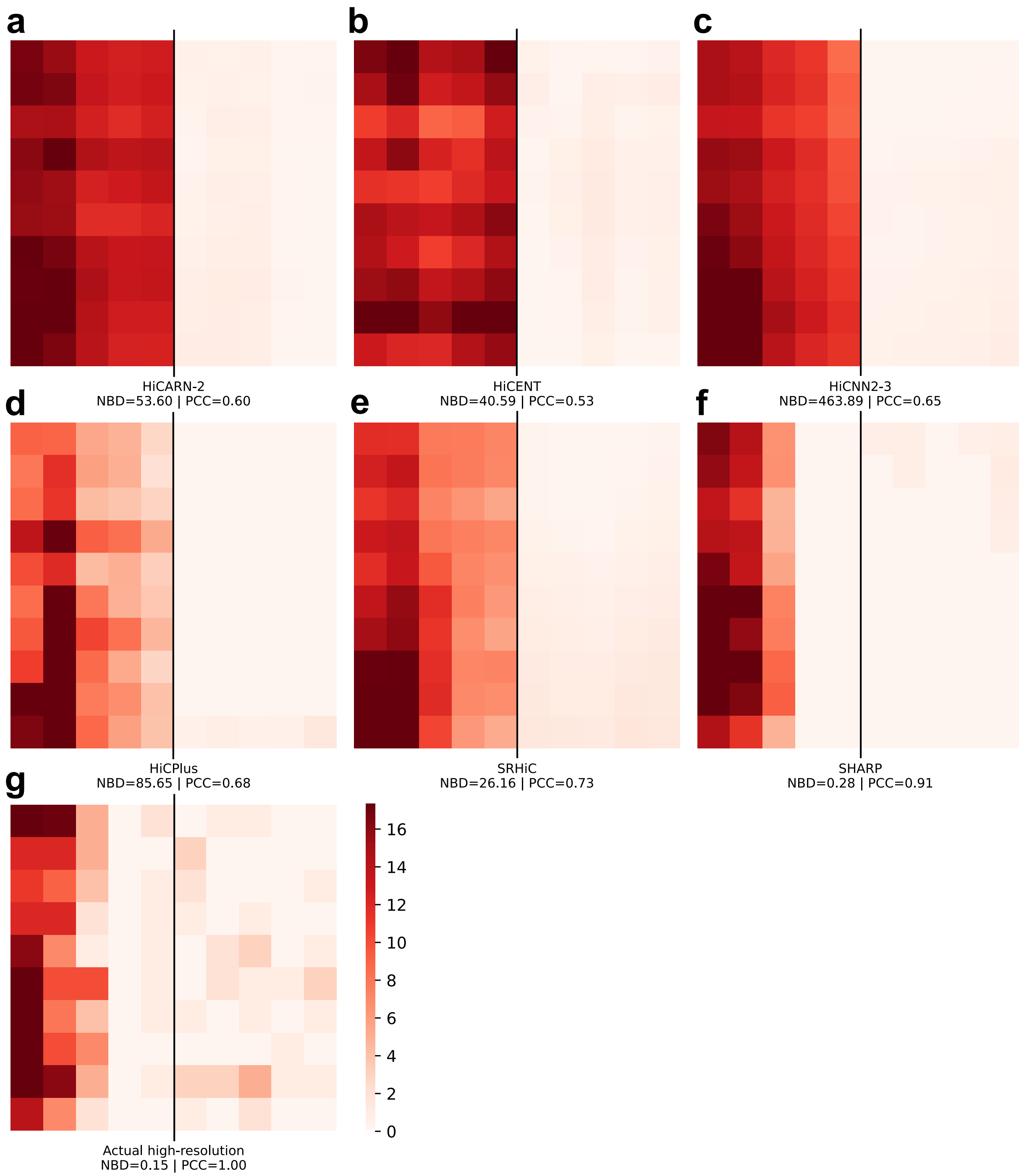

Supplementary Figure S4: Artificial structures in resolution-enhanced Hi-C contact matrices of chromosome 22 (20.815Mb-20.865Mb × 21.095Mb-21.145Mb) of GM12878 cells. a-f, The resolution-enhanced contact matrices produced by HiCARN-2 (a), HiCENT (b) HiCNN2-3 (c), HiCPlus (d), SRHiC (e), and SHARP (f). In all cases, the left half is assigned to one patch and the right half is assigned to another patch. Local PCC and NBD are reported. **g,** The actual high-resolution contact matrix of the same area.

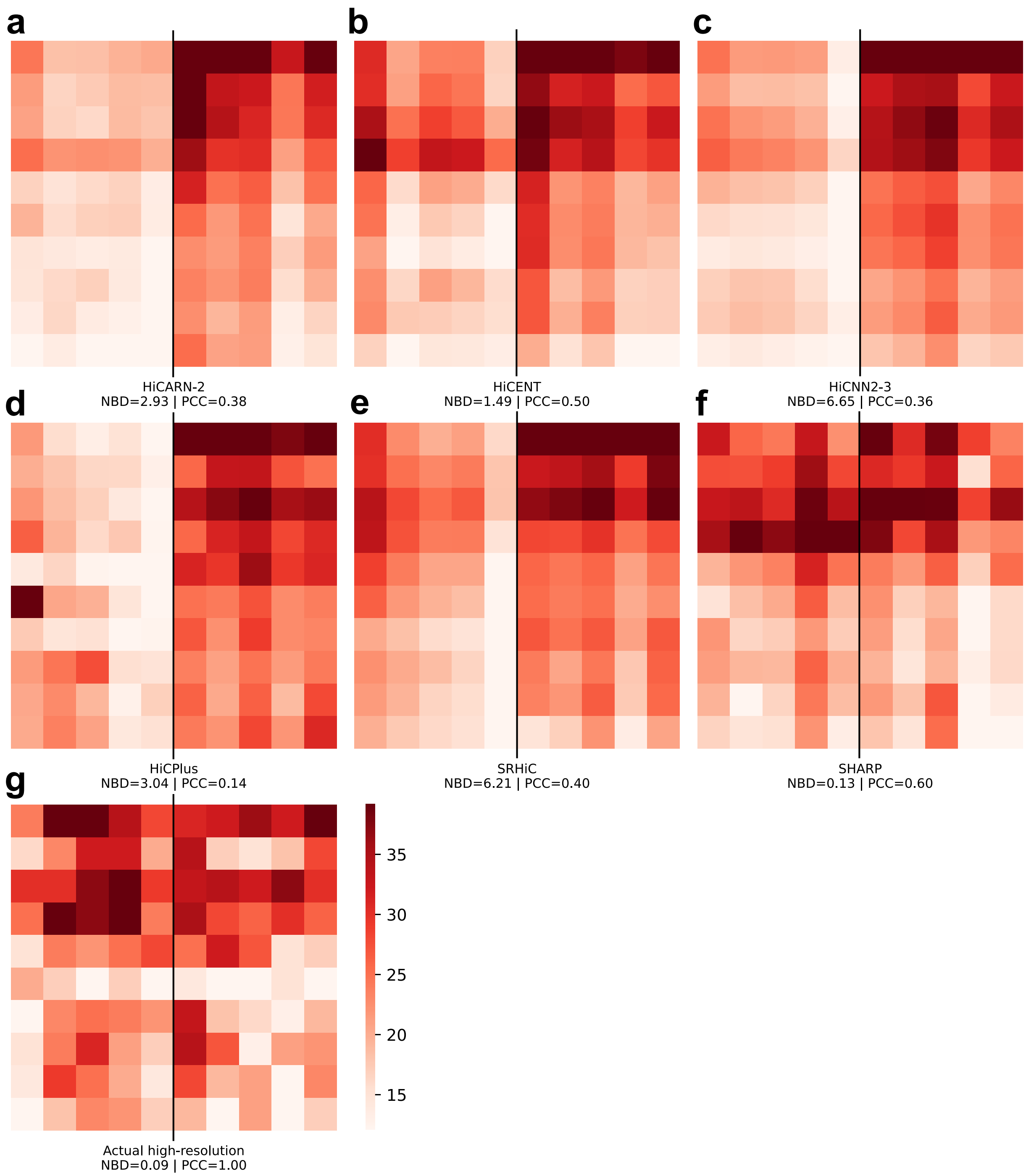

Supplementary Figure S5: Artificial structures in resolution-enhanced Hi-C contact matrices of chromosome 10 (21.945Mb-21.995Mb × 21.735Mb-21.785Mb) of GM12878 cells. a-f, The resolution-enhanced contact matrices produced by HiCARN-2 (a), HiCENT (b) HiCNN2-3 (c), HiCPlus (d), SRHiC (e), and SHARP (f). In all cases, the left half is assigned to one patch and the right half is assigned to another patch. Local PCC and NBD are reported. **g,** The actual high-resolution contact matrix of the same area.

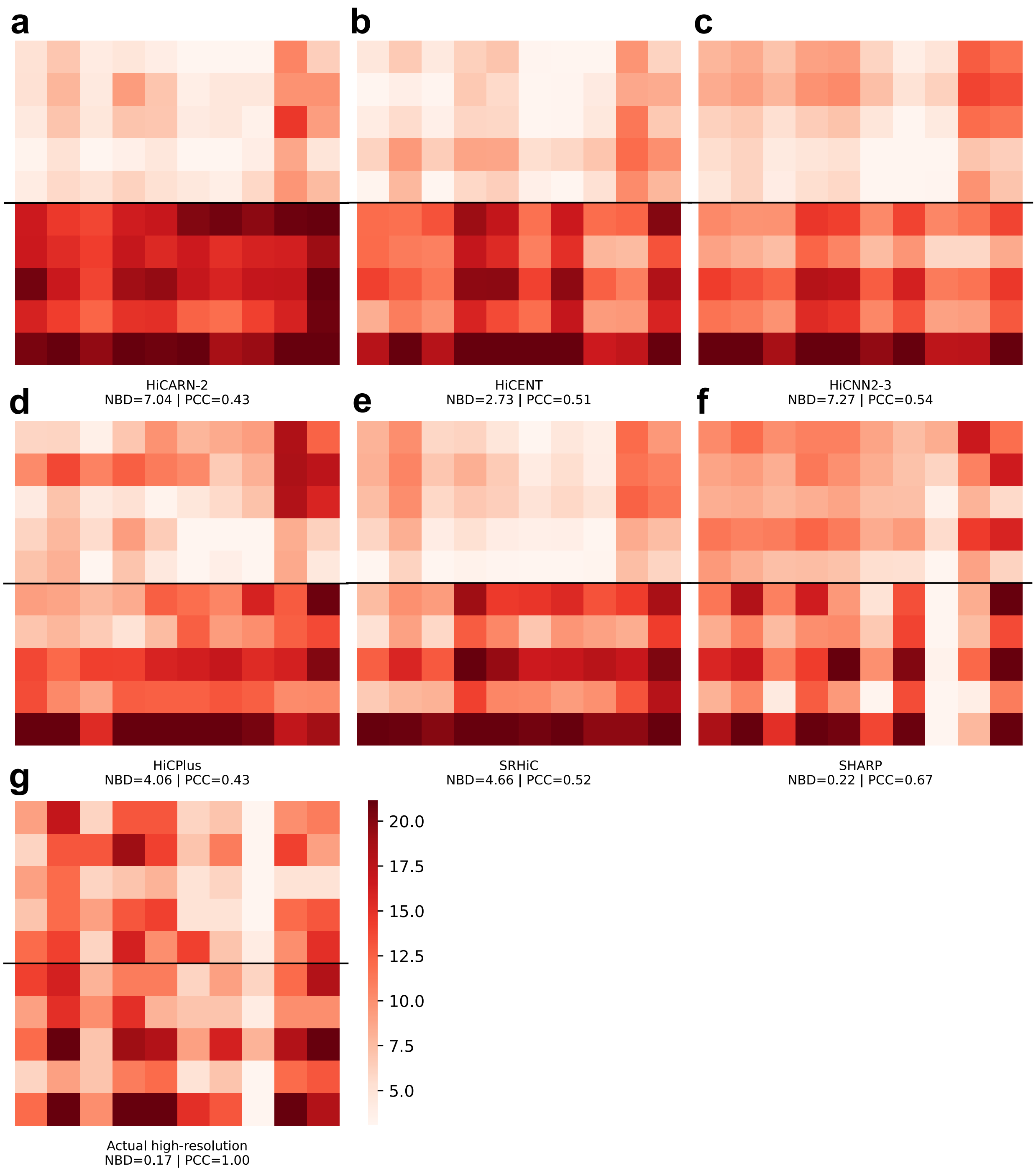

Supplementary Figure S6: Artificial structures in resolution-enhanced Hi-C contact matrices of chromosome 3 (11.175Mb-11.225Mb × 11.445Mb-11.495Mb) of GM12878 cells. a-f, The resolution-enhanced contact matrices produced by HiCARN-2 (a), HiCENT (b) HiCNN2-3 (c), HiCPlus (d), SRHiC (e), and SHARP (f). In all cases, the upper half is assigned to one patch and the lower half is assigned to another patch. Local PCC and NBD are reported. **g,** The actual high-resolution contact matrix of the same area.

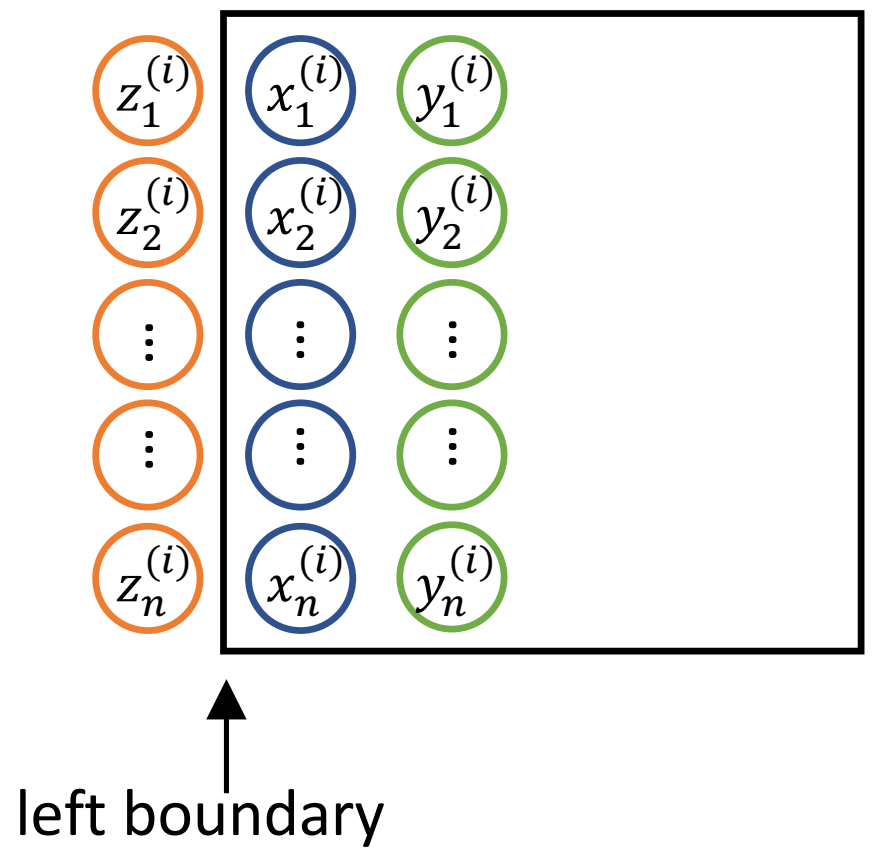

Supplementary Figure S7: Illustration of the calculation of the NBD measure. The black box shows a patch (Patch i) defined by a resolution enhancement method. The NBD measure computes the average difference across a boundary (between x and z) and uses the average difference of the immediate neighboring values within the patch (between x and y) as control. The NBD measure considers all four boundaries in this way.

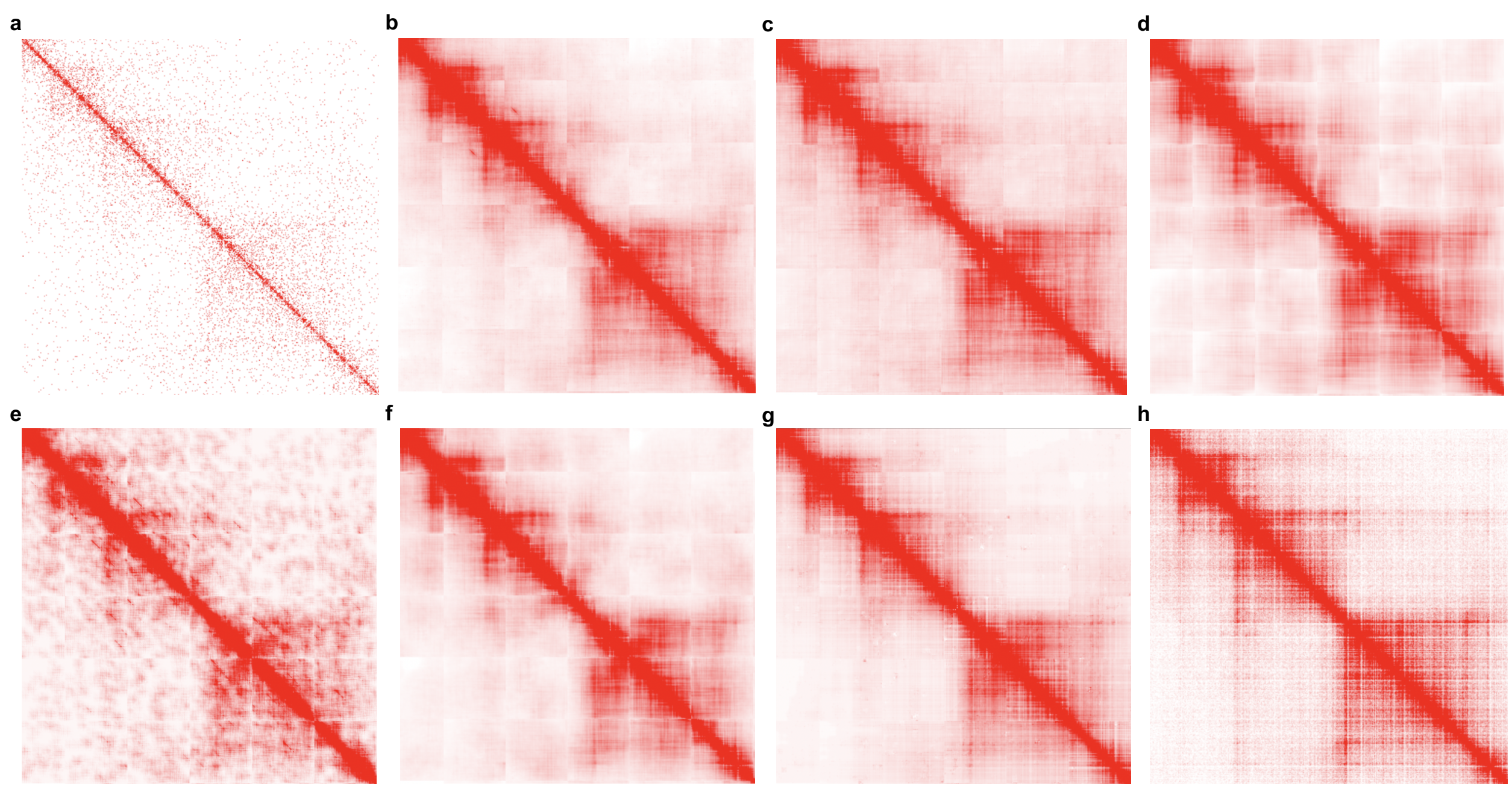

Supplementary Figure S8: Hi-C contact matrices of chromosome 18 (50.655MB-52.5MB × 50.655MB-52.5MB) reconstructed by SHARP and other methods. **a,** The actual low-resolution contact matrix. **b-g,** The resolution-enhanced contact matrix produced by HiCARN-2 (b), HiCENT (c), HiCNN2-3 (d), HiCPlus (e), SRHiC (f) and SHARP (g). **h,** The actual high-resolution contact matrix.

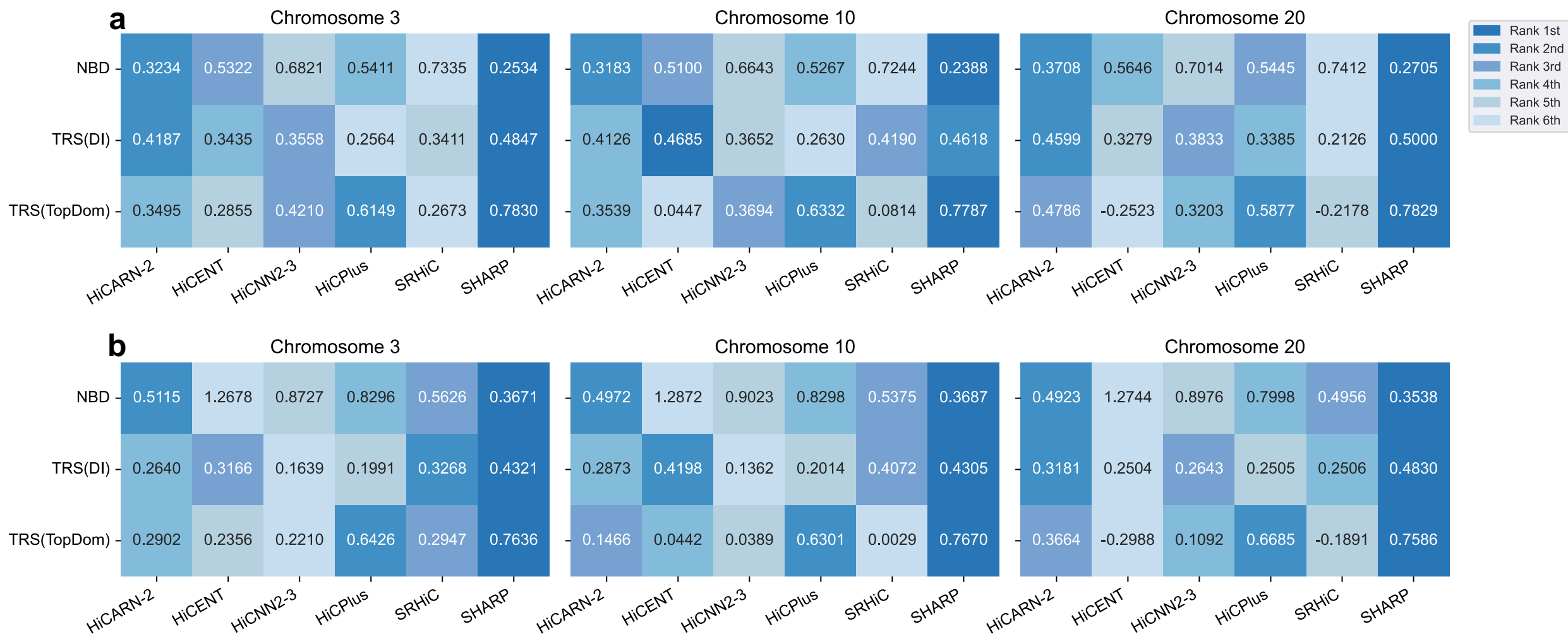

Supplementary Figure S9: Artificial structure avoidance performance of SHARP as compared to five other methods when applied to low-resolution contact matrices down-sampled from a high-resolution matrix at different sampling rates. For the NBD measure, a smaller value corresponds to better avoidance of artificial structures; for the TRS measures, a larger value corresponds to better avoidance of artificial TADs. **a,** Sampling rate of 1/50. **b,** Sampling rate of 1/100.

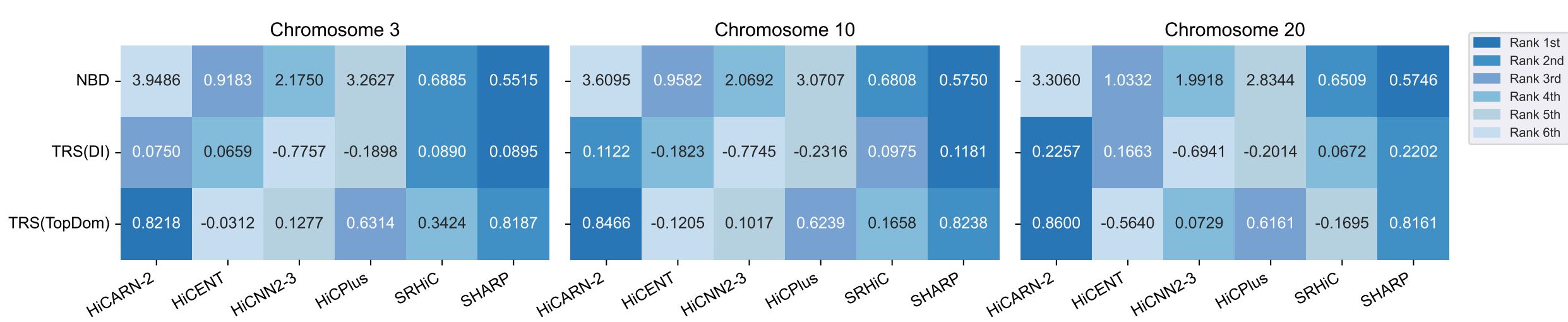

Supplementary Figure S10: Artificial structure avoidance performance of SHARP as compared to five other methods when applied to an actual low-resolution contact matrix. For the NBD measure, a smaller value corresponds to better avoidance of artificial structures; for the TRS measures, a larger value corresponds to better avoidance of artificial TADs.

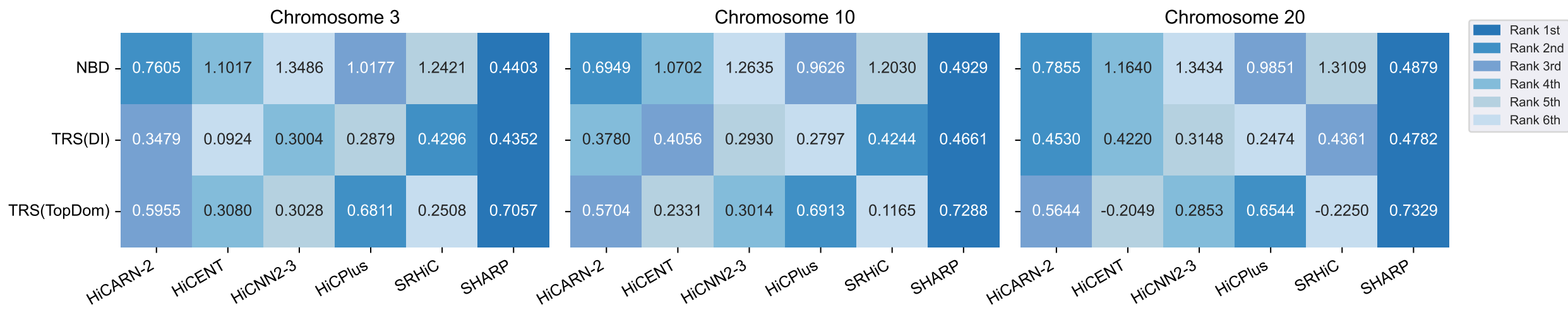

Supplementary Figure S11: Artificial structure avoidance performance of SHARP as compared to five other methods when applied to a Hi-C contact matrix of the H1-hESC cell line. For the NBD measure, a smaller value corresponds to better avoidance of artificial structures; for the TRS measures, a larger value corresponds to better avoidance of artificial TADs.

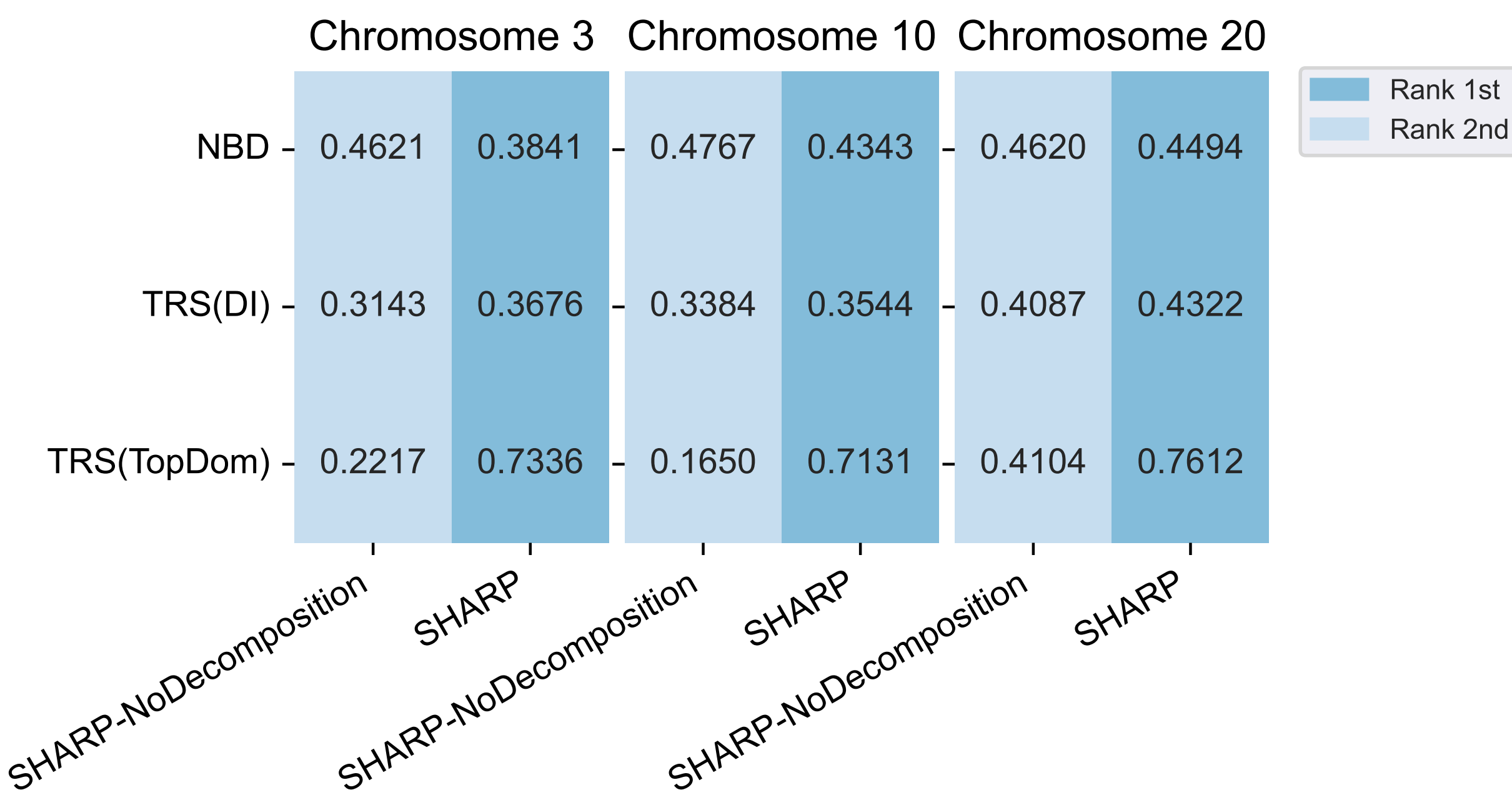

Supplementary Figure S12: Artificial structure avoidance performance of SHARP and a simplified version of it that does not perform block detection and removal (SHARP-NoDecomposition). For the NBD measure, a smaller value corresponds to better avoidance of artificial structures; for the TRS measures, a larger value corresponds to better avoidance of artificial TADs.

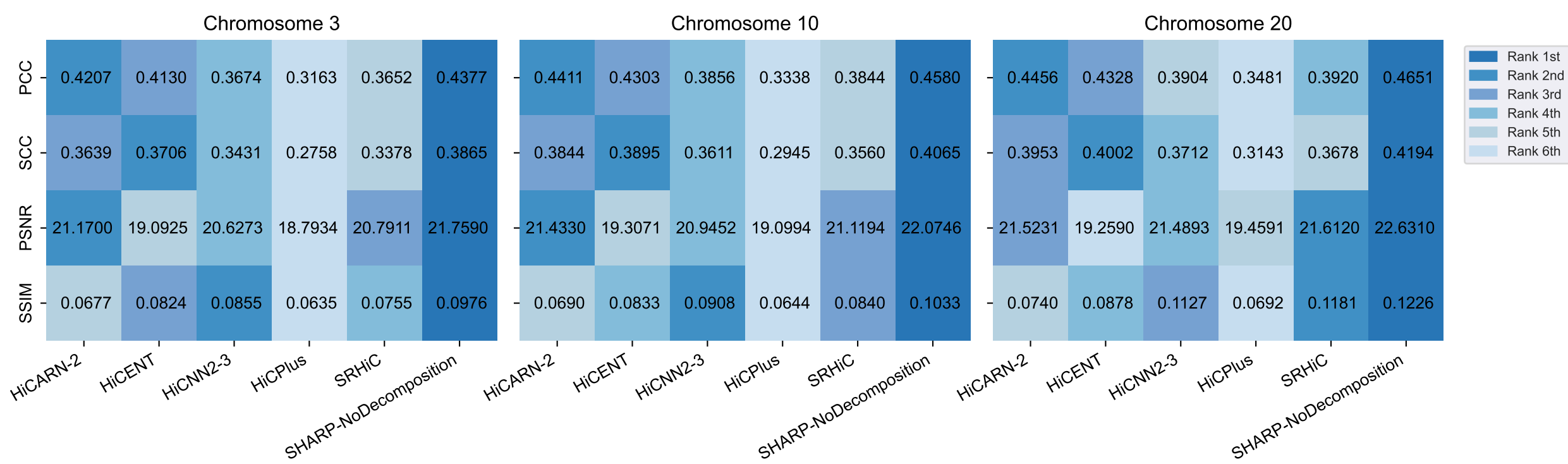

Supplementary Figure S13: Resolution enhancement performance of SHARP-NoDecomposition as compared to five other methods. For all four measures, a larger value corresponds to better performance.

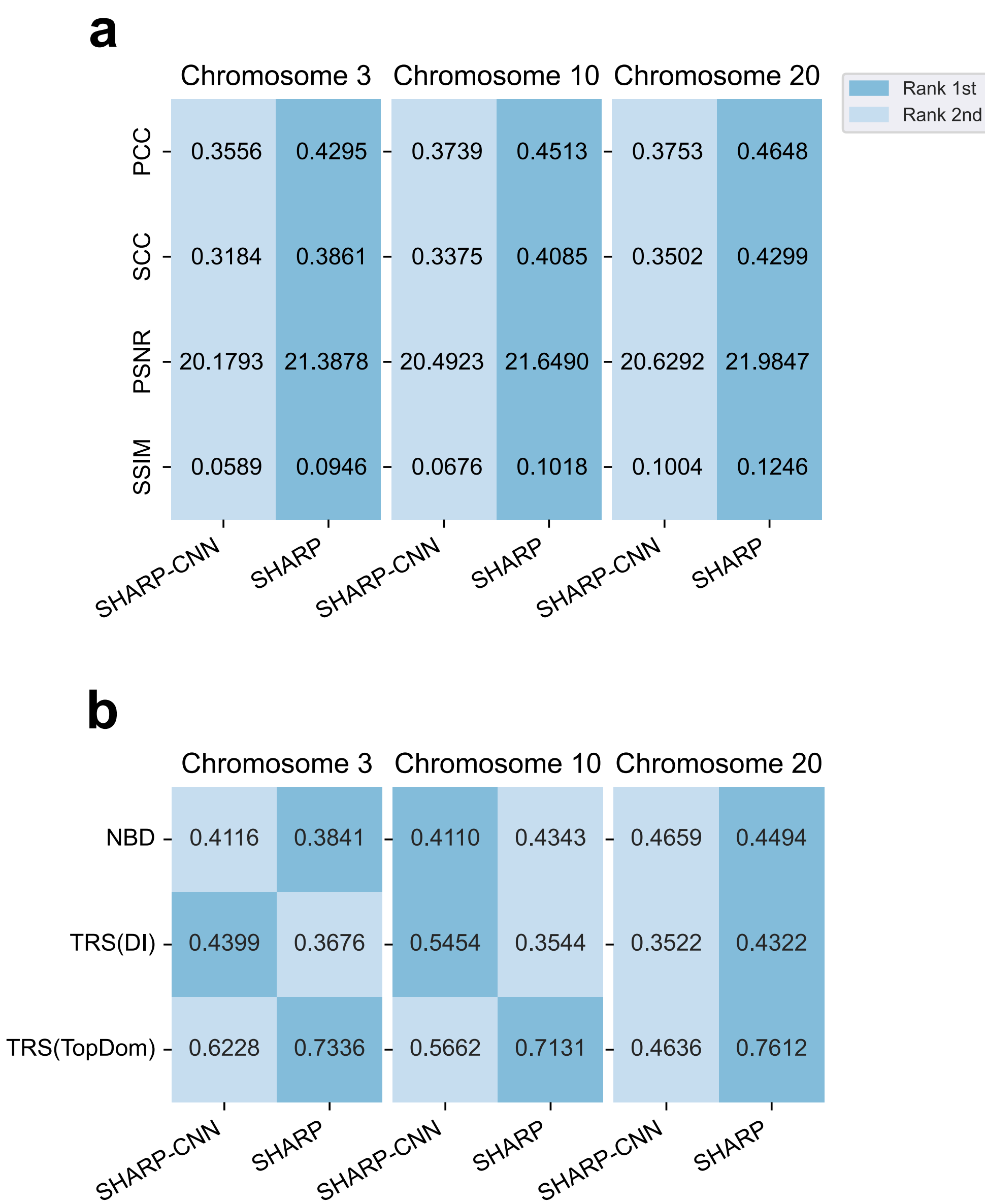

Supplementary Figure S14: Comparison between SHARP and a simplified version of it in which the MAXIM backbone is replaced with a convolutional network. **a,** Resolution enhancement performance of SHARP-CNN as compared to SHARP. For all four measures, a larger value corresponds to better performance. **b,** Artificial structure avoidance performance of SHARP-CNN as compared to SHARP. For the NBD measure, a smaller value corresponds to better avoidance of artificial structures; for the TRS measures, a larger value corresponds to better avoidance of artificial TADs.

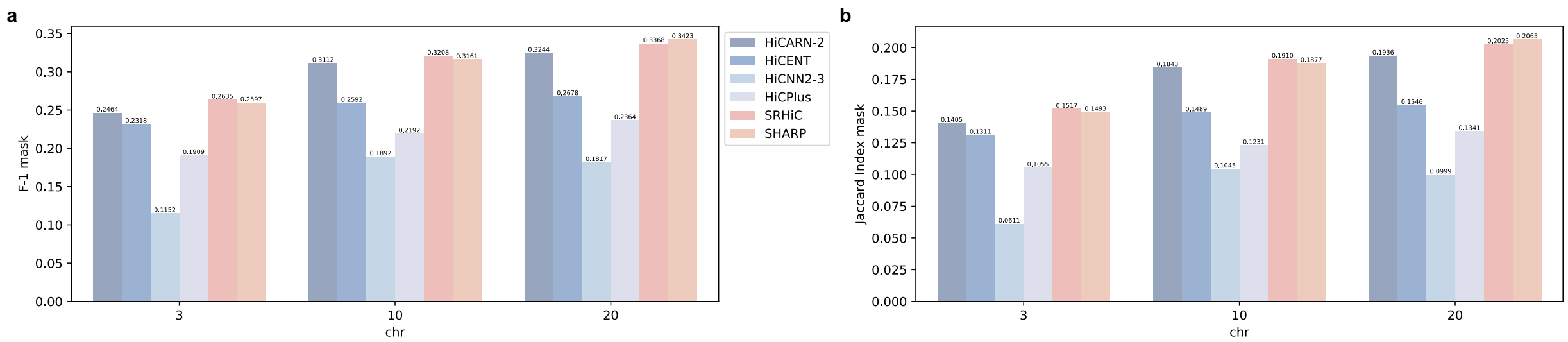

Supplementary Figure S15: Significant chromatin interactions in the imputed regions (i.e., bin pairs in the evaluation set only) identified by SHARP as compared to five other methods. a-b, Comparing the statistically significant chromatin interactions identified from the resolution-enhanced contact matrices produced by the methods with those identified from the actual high-resolution matrix, quantified by the F-1 score (a) or Jaccard Index (b).

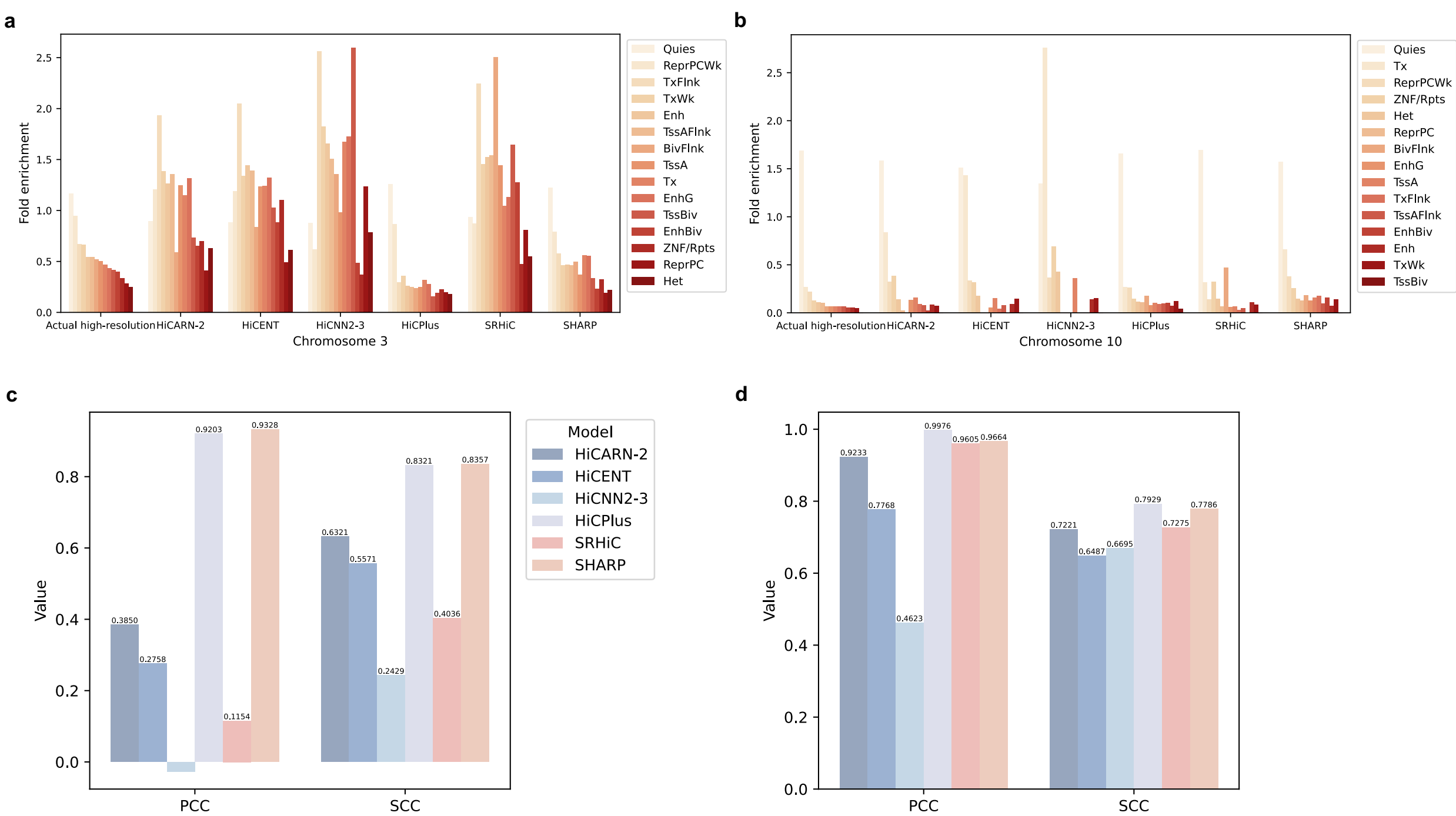

Supplementary Figure S16: Significant chromatin interactions identified by SHARP as compared to five other methods on Chr 3 and Chr 10. a-b, Enrichment of the identified chromatin interactions at different chromatin states of Chr 3 (a) and Chr 10 (b). **c-d,** Pearson correlation between the enrichment values and those computed from significant interactions identified in the actual high-resolution matrix for Chr 3 (c) and Chr 10 (d).

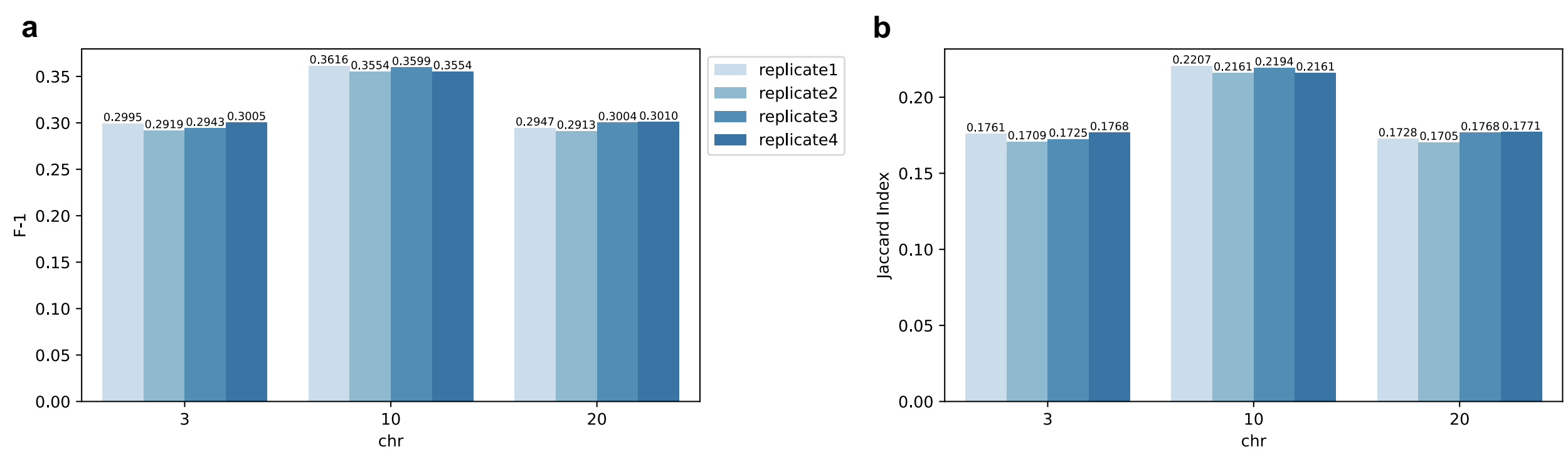

Supplementary Figure S17: Significant chromatin interactions in the whole matrix identified by four replicates. a-b, Comparing the statistically significant chromatin interactions identified from the resolution-enhanced contact matrices produced by the replicates with those identified from the actual high-resolution matrix, quantified by the F-1 score (a) or Jaccard Index (b).

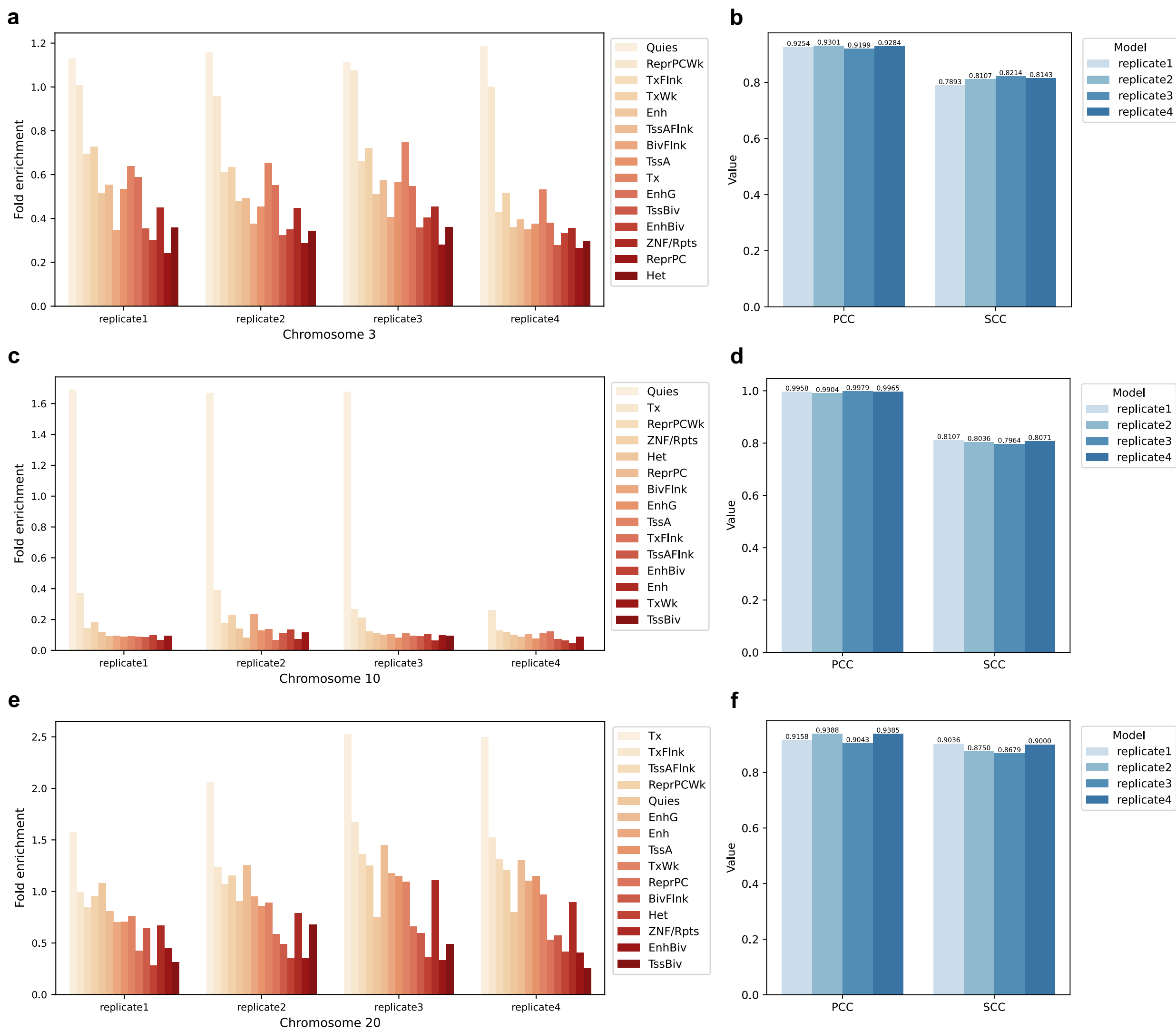

Supplementary Figure S18: Significant chromatin interactions identified by four replicates. a, Enrichment of the identified chromatin interactions at different chromatin states of Chr 3. **b,** Correlation of the enrichment values with those computed based on significant interactions identified from the actual high-resolution matrix of Chr 3. **c,** Enrichment of the identified chromatin interactions at different chromatin states of Chr 10. **d,** Correlation of the enrichment values with those computed based on significant interactions identified from the actual high-resolution matrix of Chr 10. **e,** Enrichment of the identified chromatin interactions at different chromatin states of Chr 20. **f,** Correlation of the enrichment values with those computed based on significant interactions identified from the actual high-resolution matrix of Chr 20.

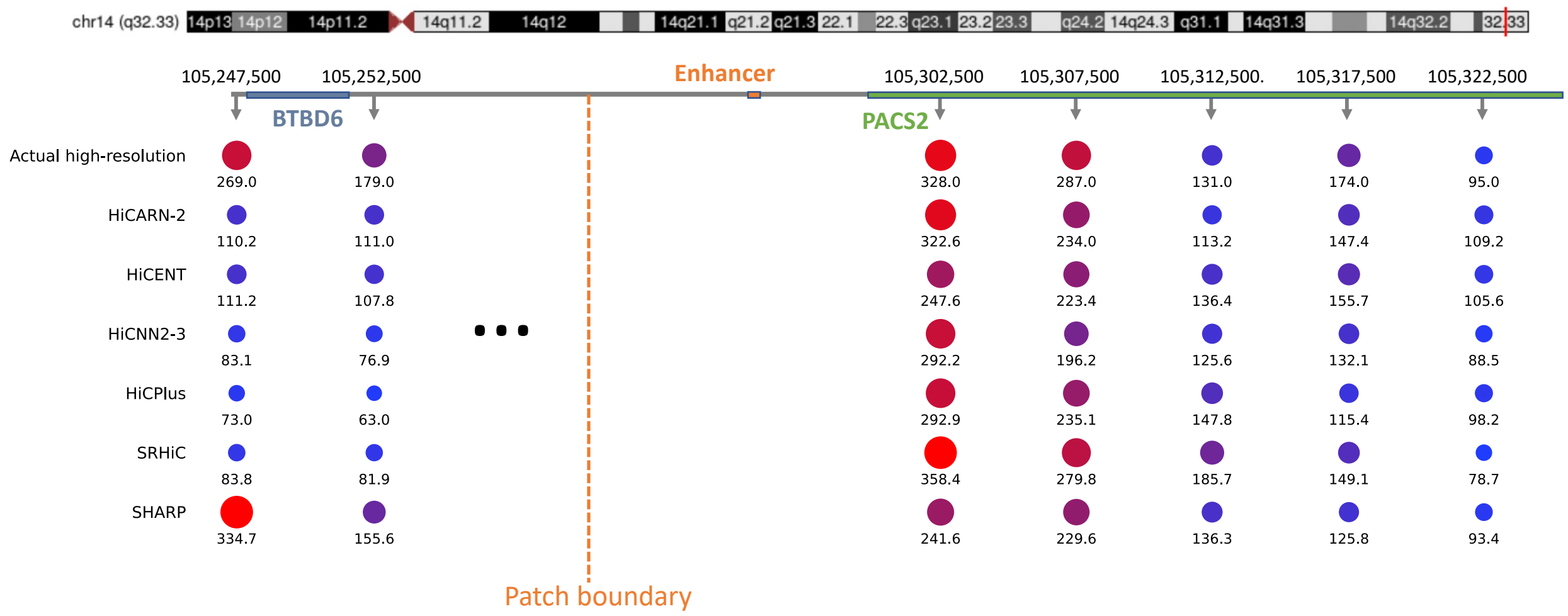

Supplementary Figure S19: Specific CRE-gene interactions identified by the different methods. All methods successfully reconstruct the strong interaction between an enhancer and PACS2, which are located within the same patch. However, only SHARP recovers the interaction between the enhancer and BTBD6, which spans across patch boundaries; this interaction is supported by the corresponding high-resolution contact map.

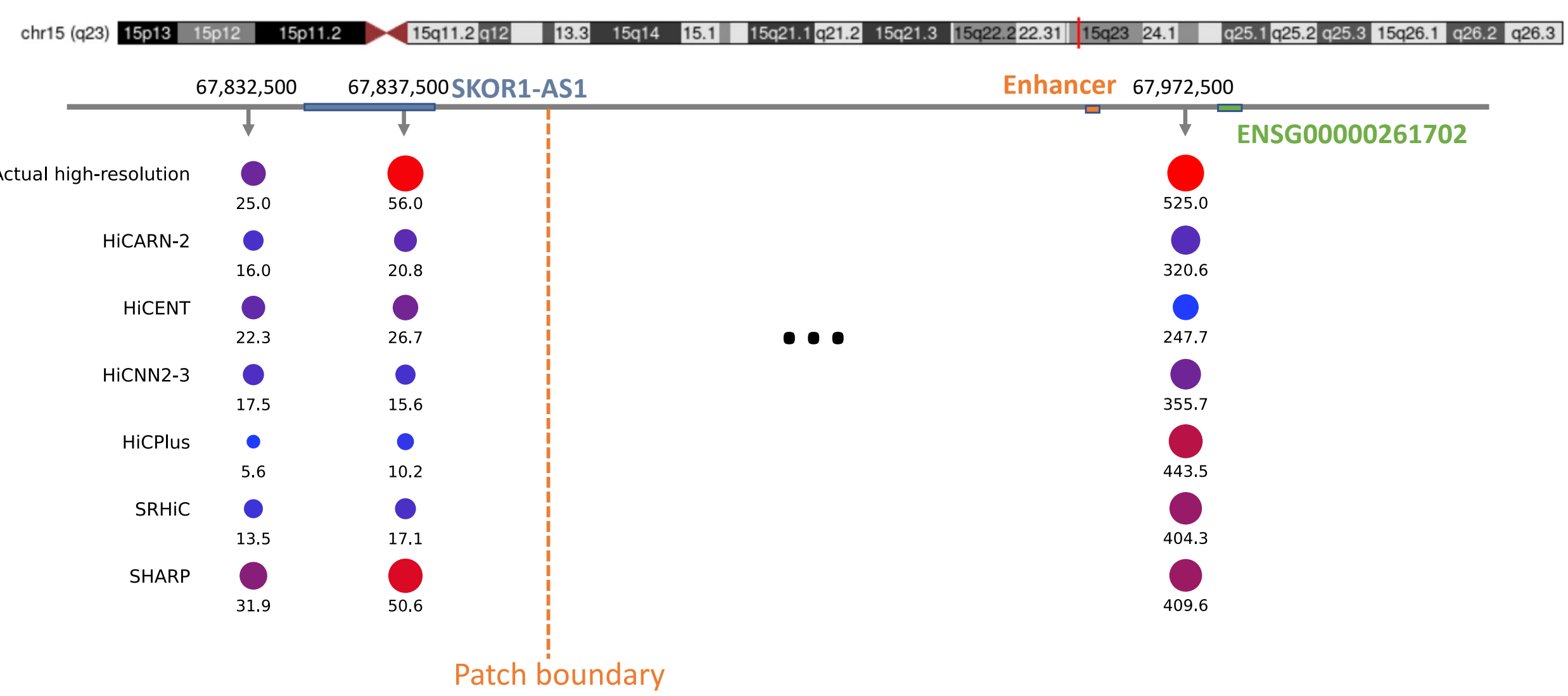

Supplementary Figure S20: Specific CRE-gene interactions identified by the different methods. Most methods reconstruct the interaction between an enhancer and ENSG00000261702 within the same patch, but only SHARP captures the interaction between the enhancer and SKOR1-AS1, which lies across different patches.

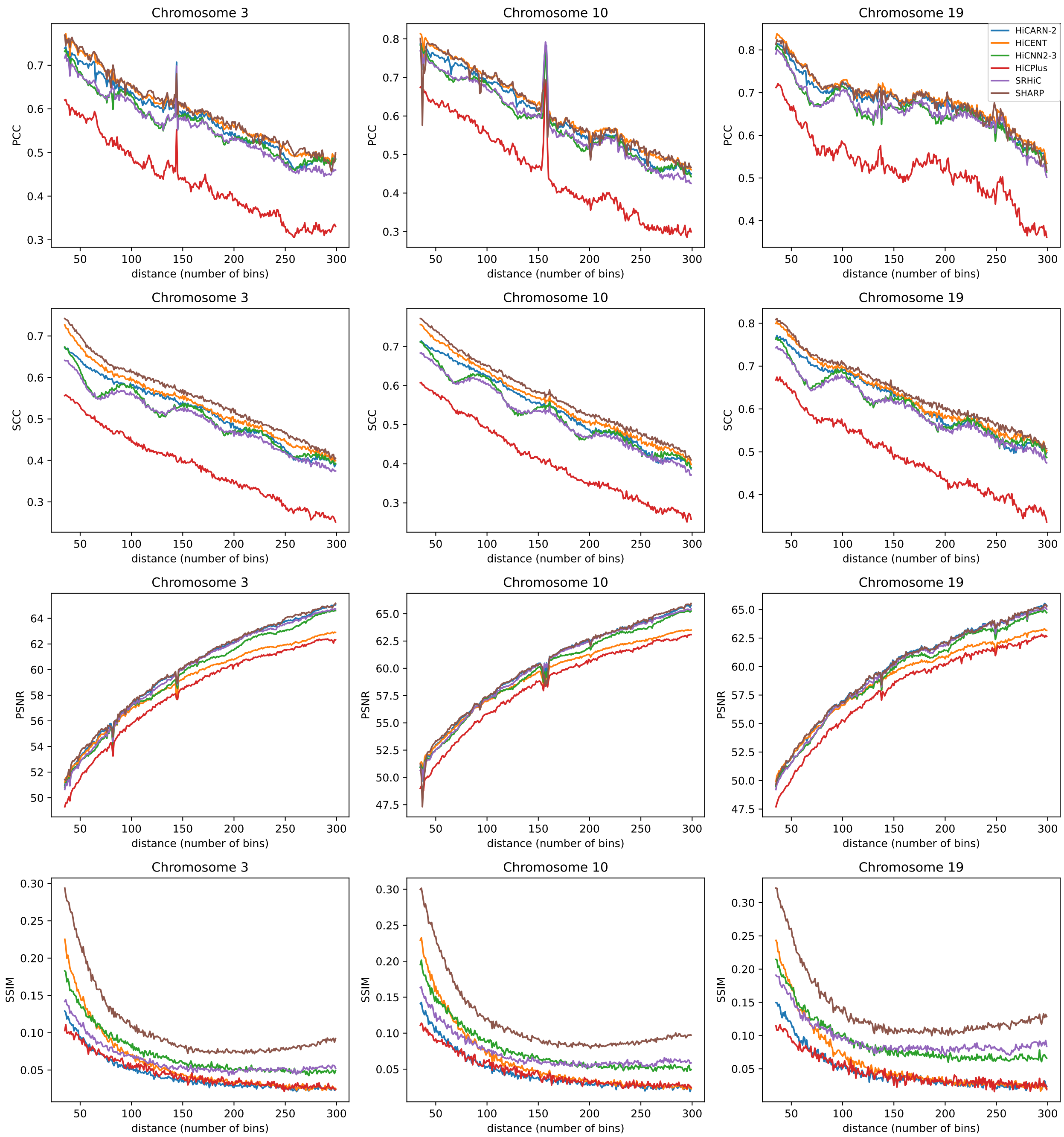

Supplementary Figure S21: Performance of the methods for region pairs at different genomic distances for mESC cells. a, PCC. **b,** SCC. **c,** PSNR. **d,** SSIM.

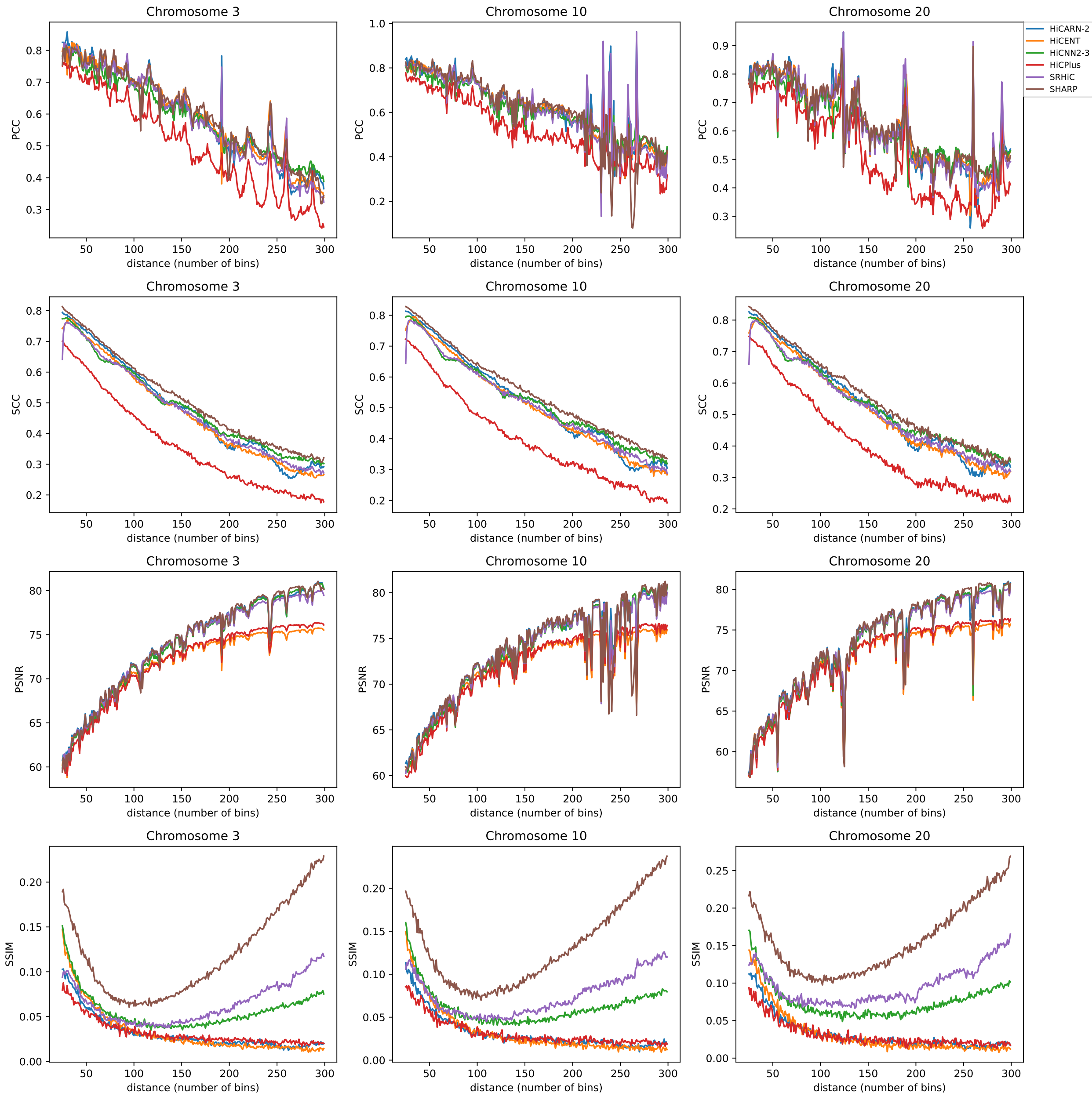

Supplementary Figure S22: Performance of the methods for region pairs at different genomic distances for HFFc6 cells. a, PCC. **b,** SCC. **c,** PSNR. **d,** SSIM.

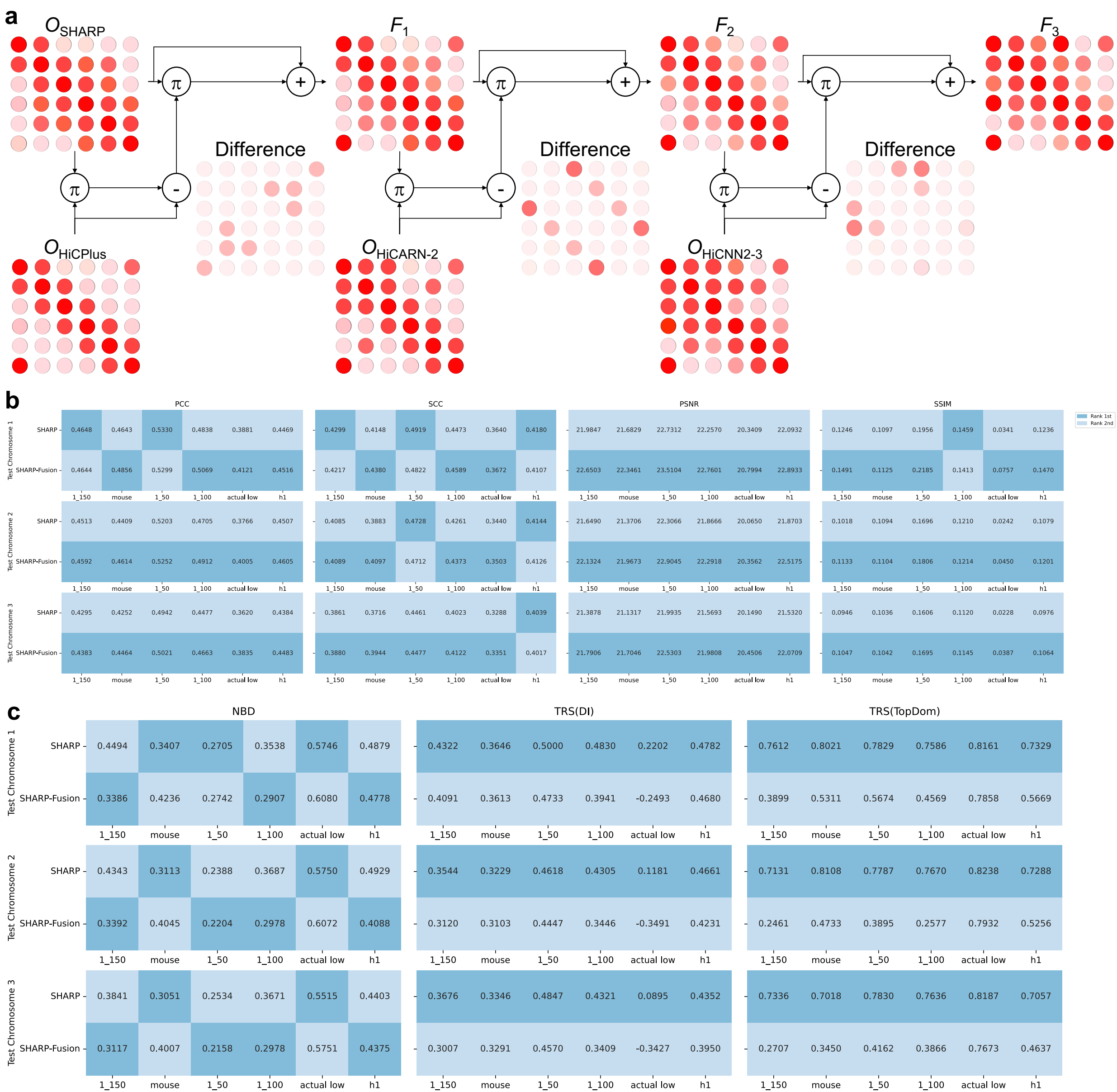

Supplementary Figure S23: Overview of SHARP-Fusion. a, The method of SHARP-Fusion. **b,** Resolution enhancement performance of SHARP-Fusion as compared to SHARP. **c,** Artificial structure avoidance performance of SHARP-Fusion as compared to SHARP.

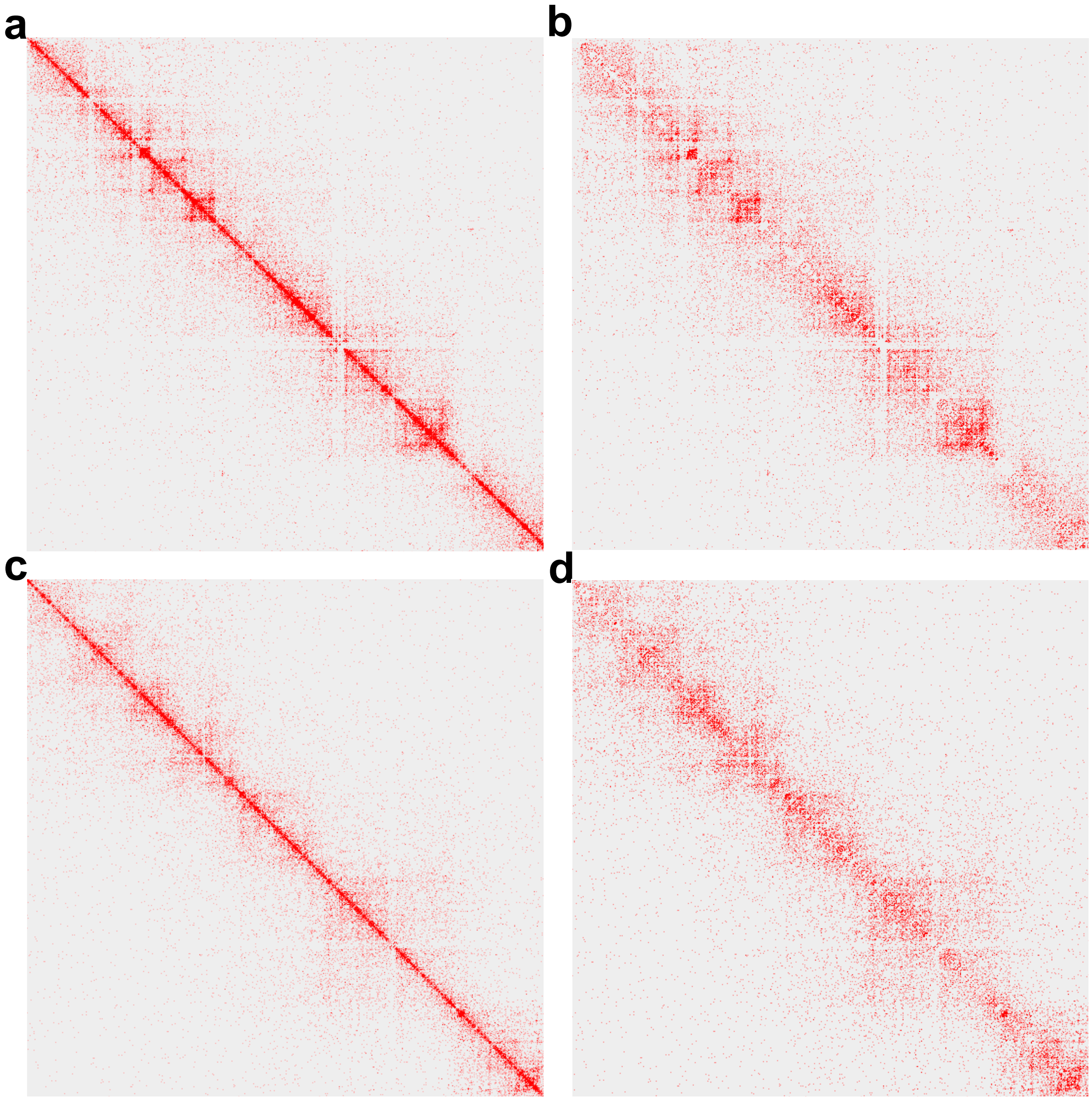

Supplementary Figure S24: Removing signals due to 1D proximity from the low-resolution GM12878 matrix at subsampling rate of 1/150. a-b, The original low-resolution data for the region Chr22:22.3Mb-25Mb (a) and the resulting data after removing signals due to 1D proximity using our formula (b). **c-d,** The original low-resolution data for the region Chr22:36.5Mb-39.2Mb (c) and the resulting data after removing signals due to 1D proximity using our formula (d).

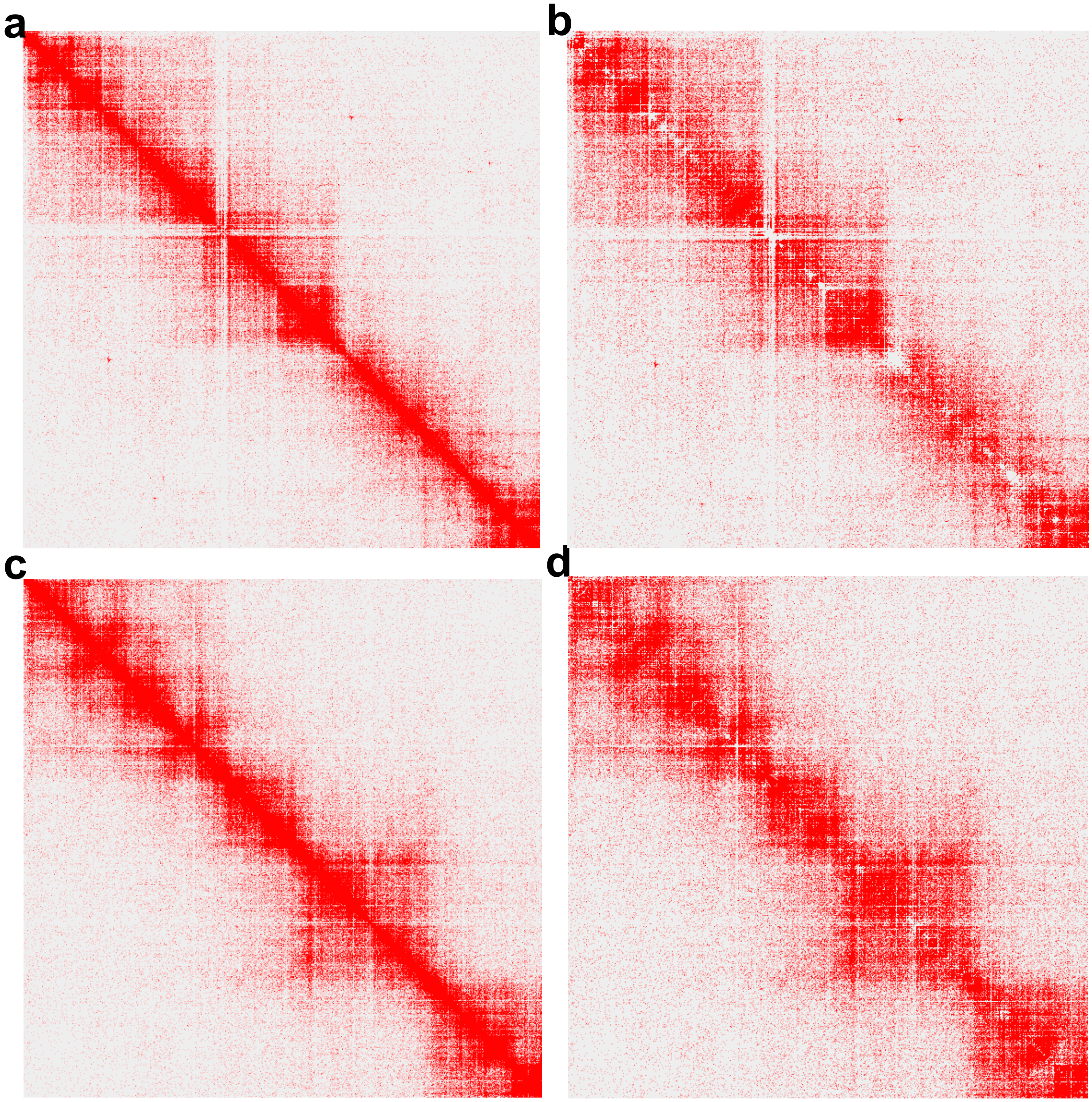

Supplementary Figure S25: Removing signals due to 1D proximity from the high-resolution GM12878 matrix. a-b, The original high-resolution data for the region Chr22:22.3Mb-25Mb (a) and the resulting data after removing signals due to 1D proximity using our formula (b). **c-d,** The original high-resolution data for the region Chr22:36.5Mb-39.2Mb (c) and the resulting data after removing signals due to 1D proximity using our formula (d).

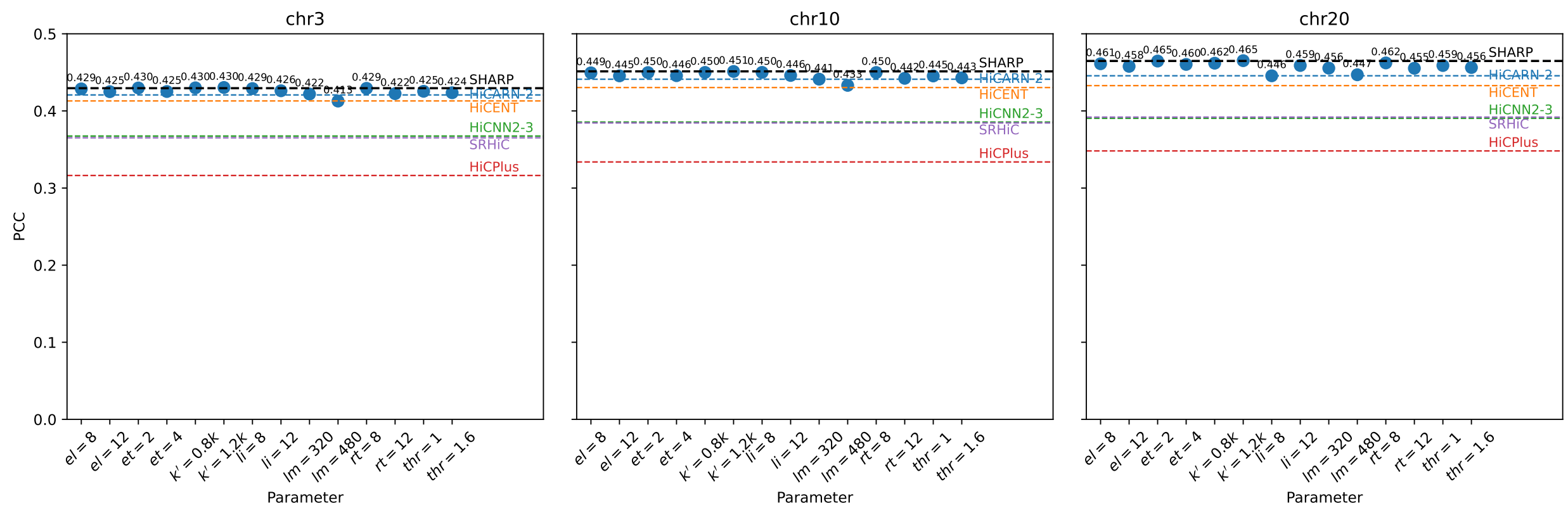

Supplementary Figure S26: Sensitivity analysis of SHARP across key hyperparameters evaluated using PCC. Scatter plots show PCC performance variations under ±20% perturbations of parameters controlling 1D proximity modeling and rectangle detection. Each panel corresponds to a chromosome, with points representing different parameter settings. Horizontal dashed lines denote baseline performance of SHARP and competing methods.

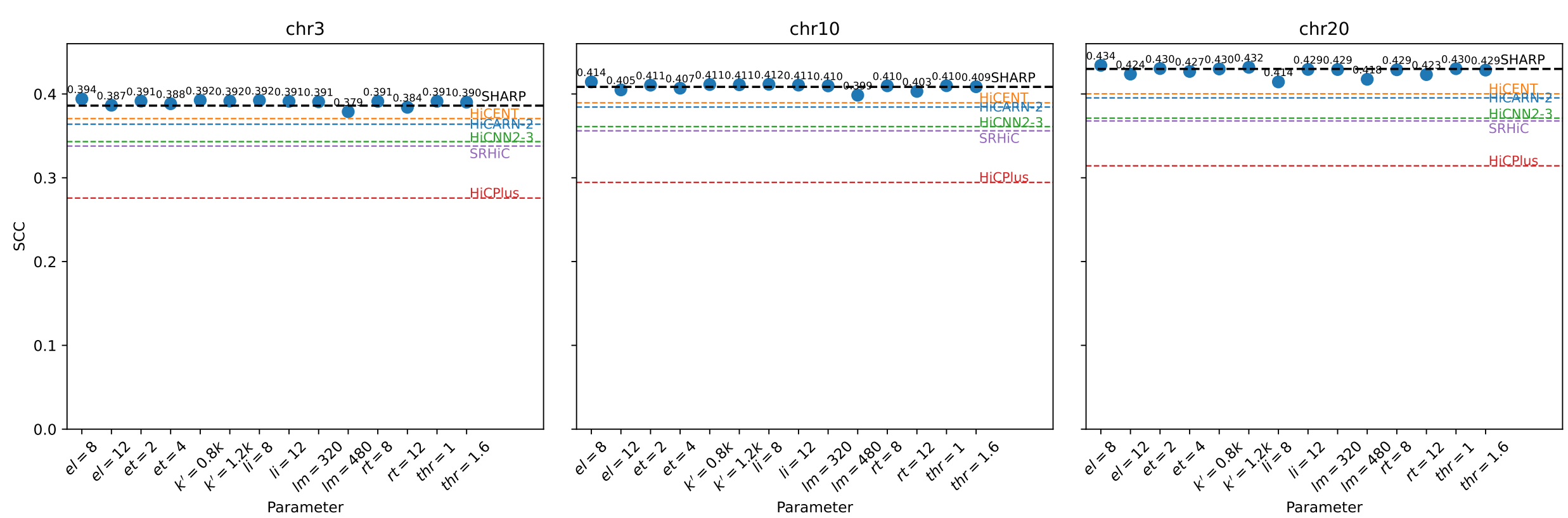

Supplementary Figure S27: Sensitivity analysis of SHARP across key hyperparameters evaluated using SCC. Scatter plots show SCC performance variations under ±20% perturbations of parameters controlling 1D proximity modeling and rectangle detection. Each panel corresponds to a chromosome, with points representing different parameter settings. Horizontal dashed lines denote baseline performance of SHARP and competing methods.

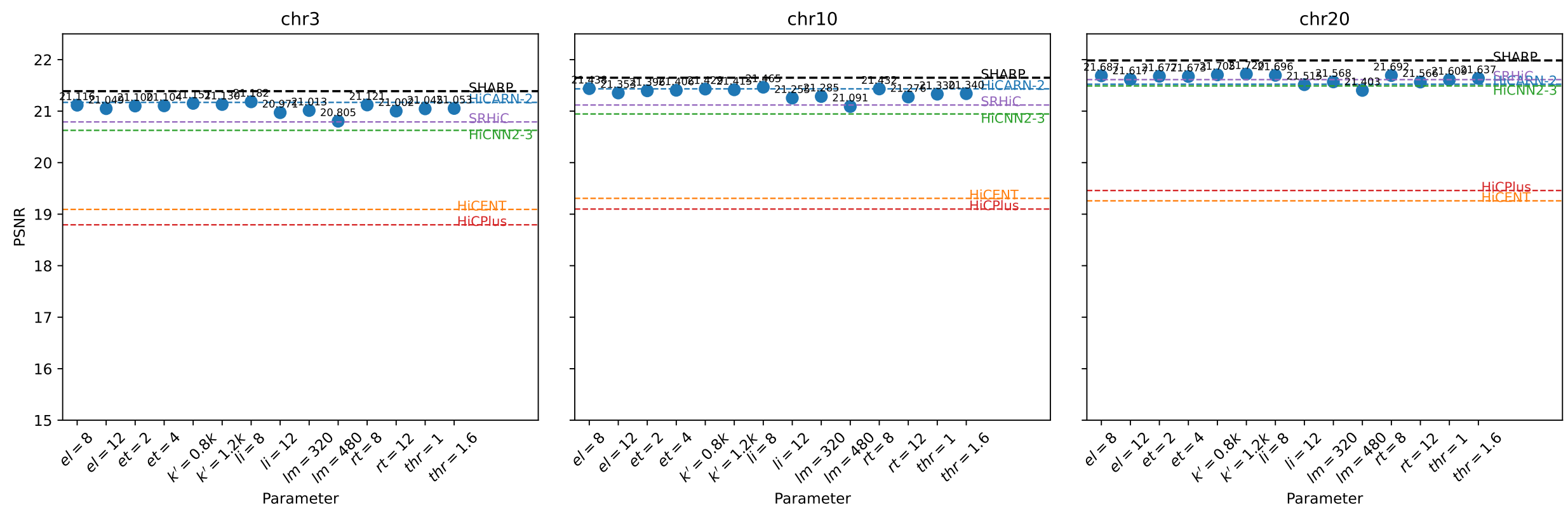

Supplementary Figure S28: Sensitivity analysis of SHARP across key hyperparameters evaluated using PSNR. Scatter plots show PSNR performance variations under ±20% perturbations of parameters controlling 1D proximity modeling and rectangle detection. Each panel corresponds to a chromosome, with points representing different parameter settings. Horizontal dashed lines denote baseline performance of SHARP and competing methods.

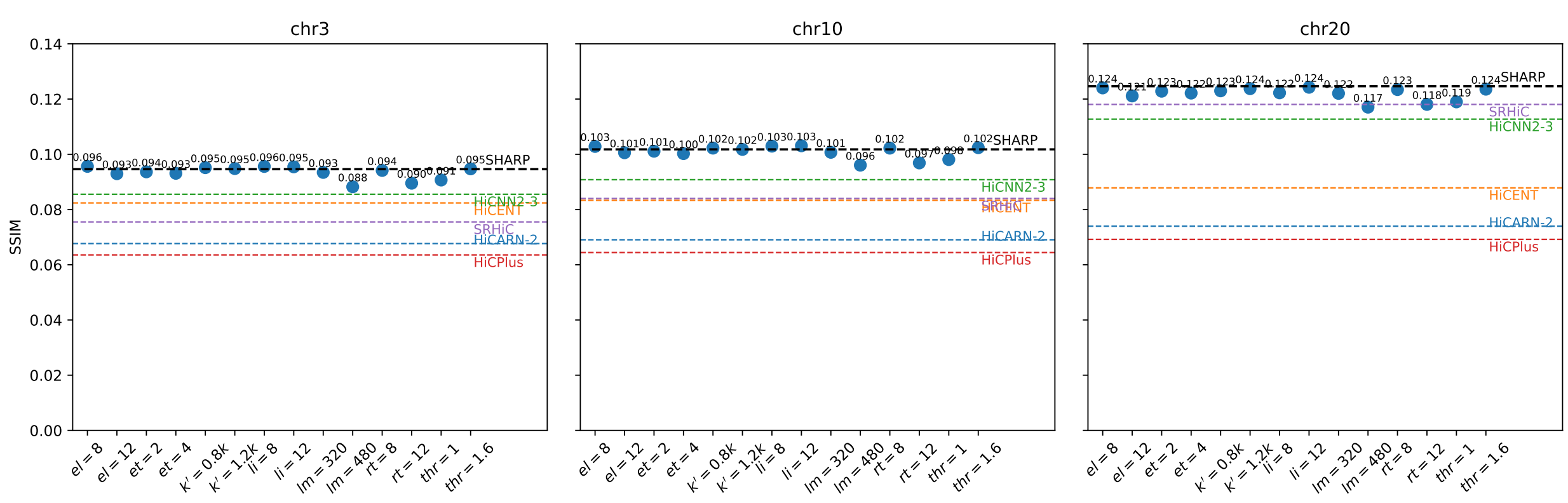

Supplementary Figure S29: Sensitivity analysis of SHARP across key hyperparameters evaluated using SSIM. Scatter plots show SSIM performance variations under ±20% perturbations of parameters controlling 1D proximity modeling and rectangle detection. Each panel corresponds to a chromosome, with points representing different parameter settings. Horizontal dashed lines denote baseline performance of SHARP and competing methods.

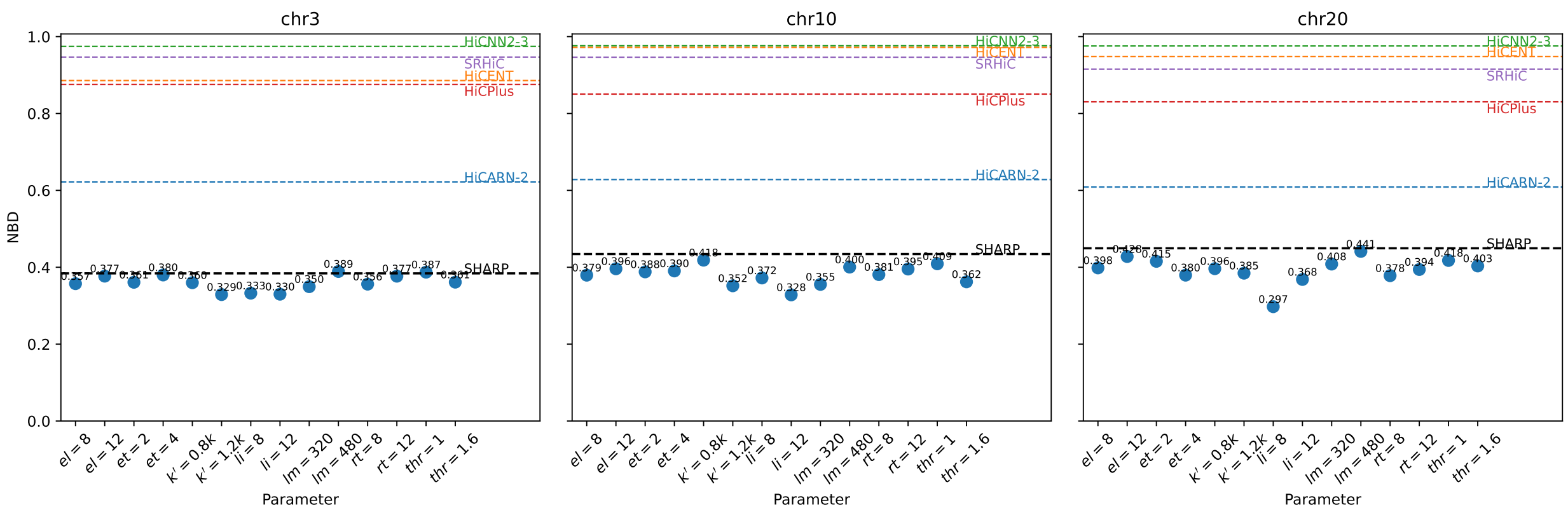

Supplementary Figure S30: Sensitivity analysis of SHARP across key hyperparameters evaluated using NBD. Scatter plots show NBD performance variations under ±20% perturbations of parameters controlling 1D proximity modeling and rectangle detection. Each panel corresponds to a chromosome, with points representing different parameter settings. Horizontal dashed lines denote baseline performance of SHARP and competing methods.

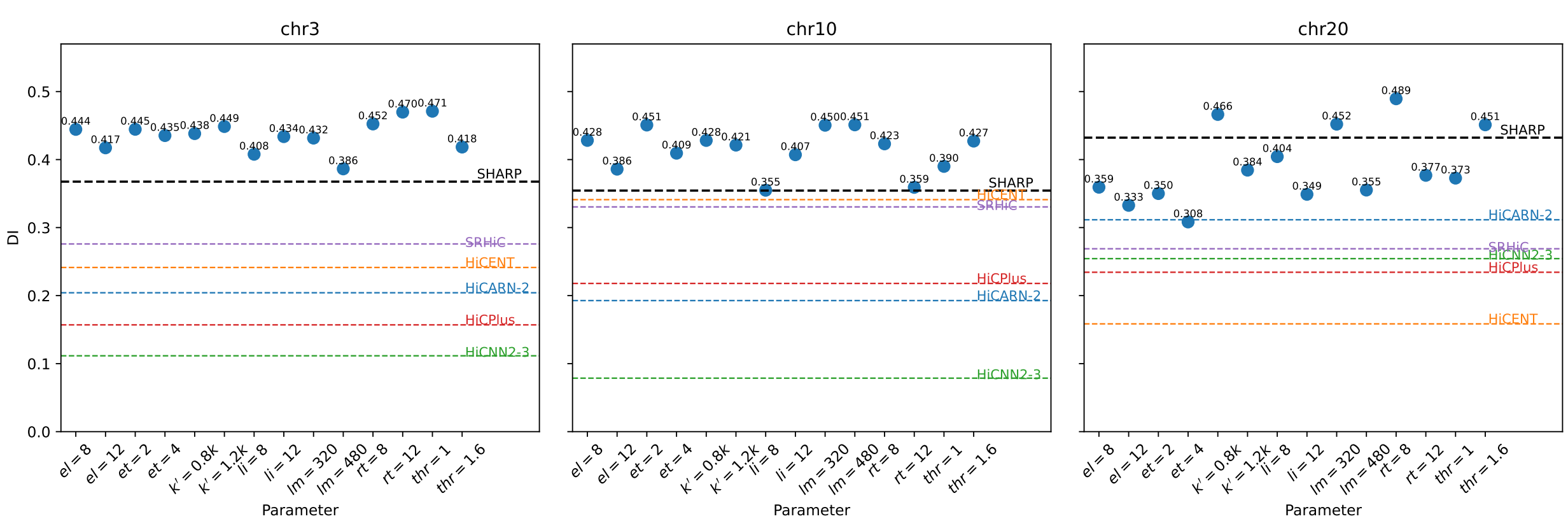

Supplementary Figure S31: Sensitivity analysis of SHARP across key hyperparameters evaluated using TRS (DI). Scatter plots show TRS (DI) performance variations under ±20% perturbations of parameters controlling 1D proximity modeling and rectangle detection. Each panel corresponds to a chromosome, with points representing different parameter settings. Horizontal dashed lines denote baseline performance of SHARP and competing methods.

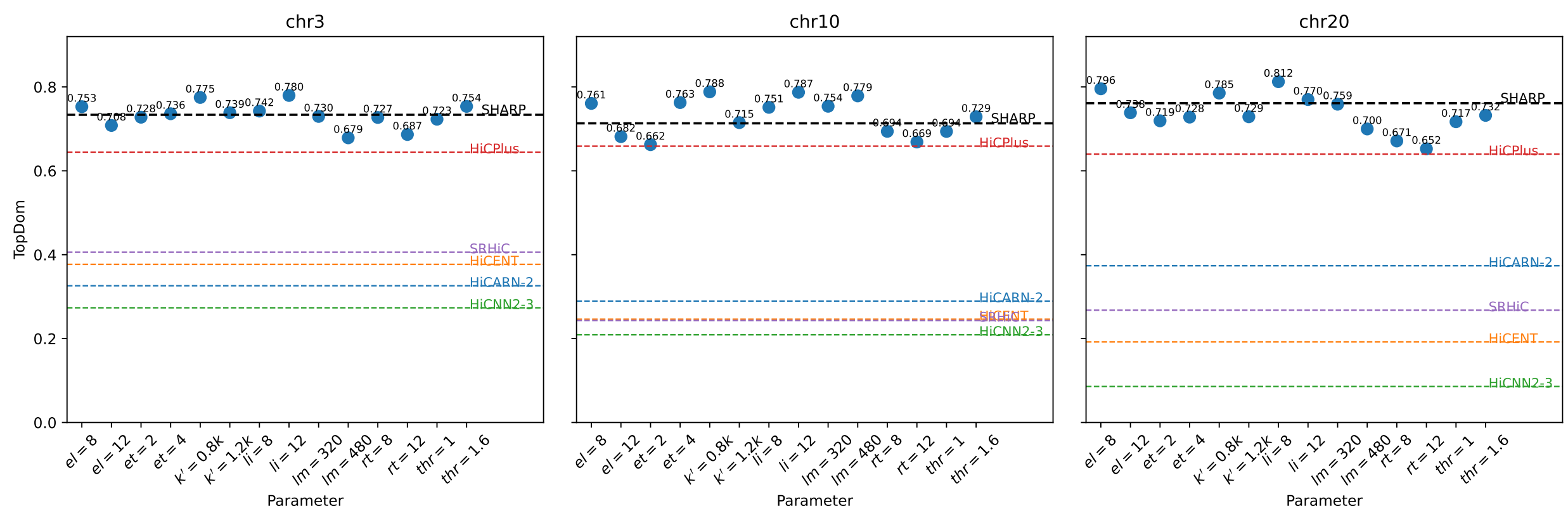

Supplementary Figure S32: Sensitivity analysis of SHARP across key hyperparameters evaluated using TRS (TopDom). Scatter plots show TRS (TopDom) performance variations under ±20% perturbations of parameters controlling 1D proximity modeling and rectangle detection. Each panel corresponds to a chromosome, with points representing different parameter settings. Horizontal dashed lines denote baseline performance of SHARP and competing methods.

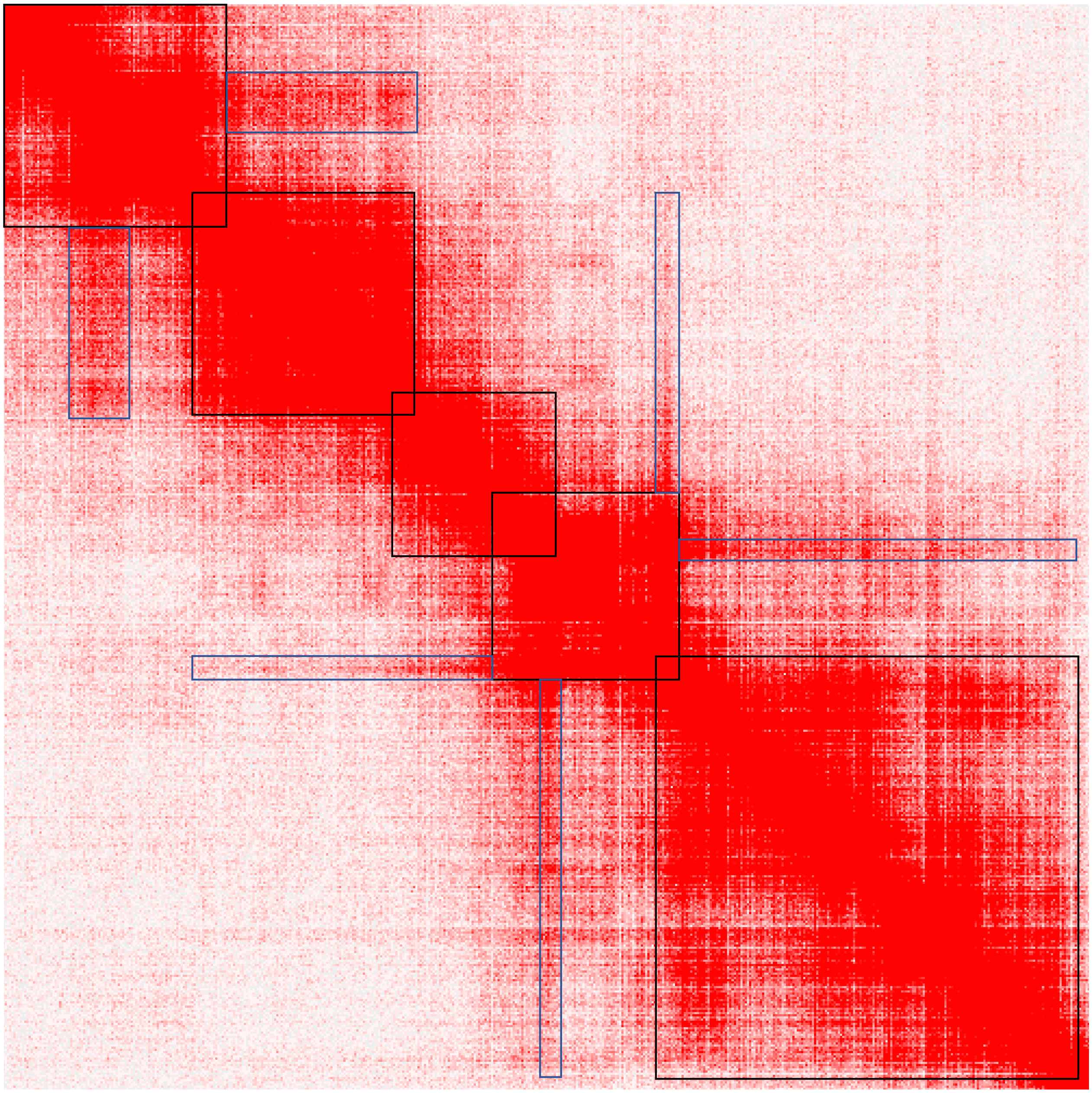

Supplementary Figure S33: Signals due to contiguous domains. Examples of the within-domain signals on the main diagonal and the between-domain signals off-diagonal are highlighted in black and blue boxes, respectively.

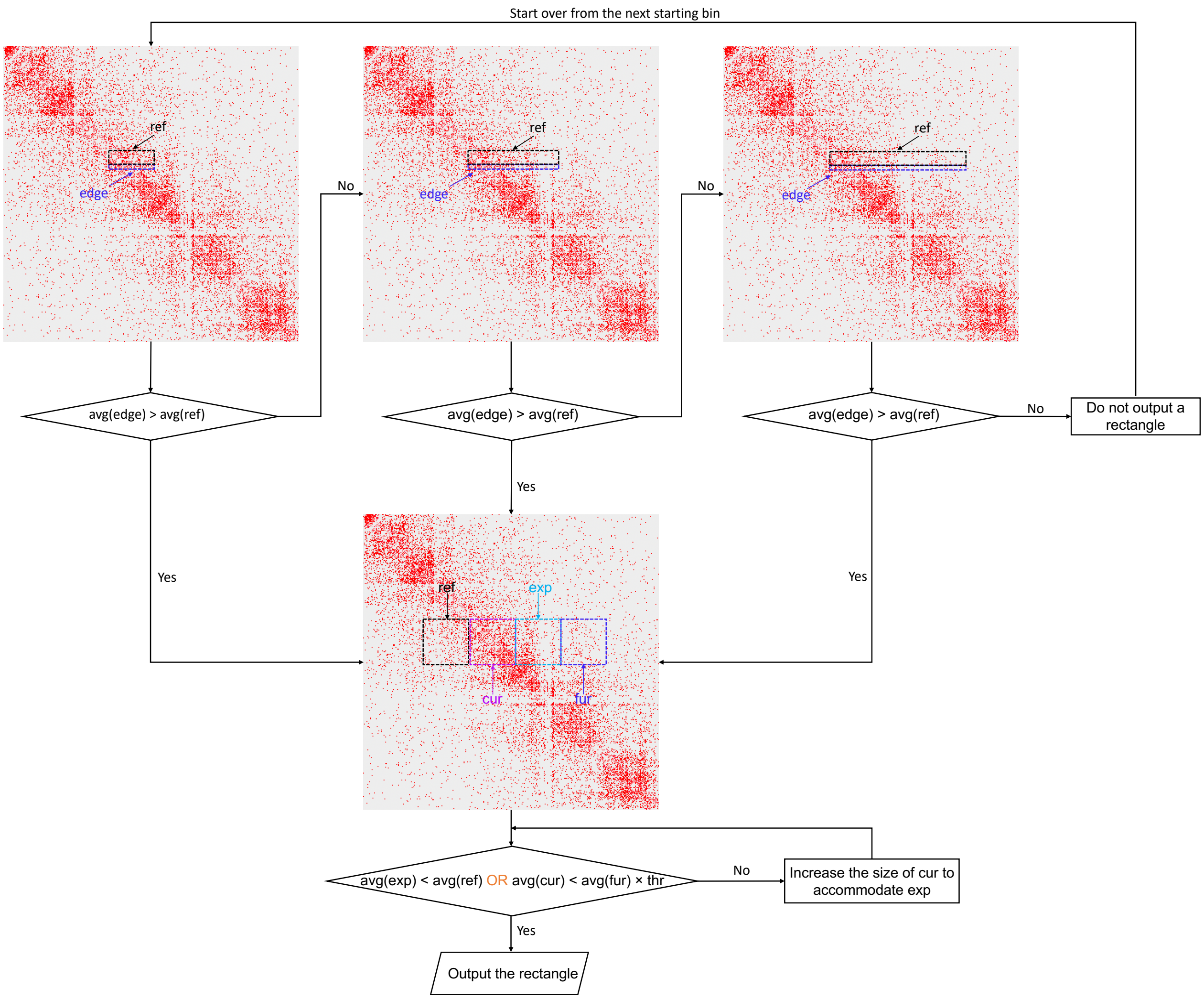

Supplementary Figure S34: Detection of on-diagonal rectangles caused by contiguous domains. The procedure is illustrated by a flow chart and described in detail in the Methods section.

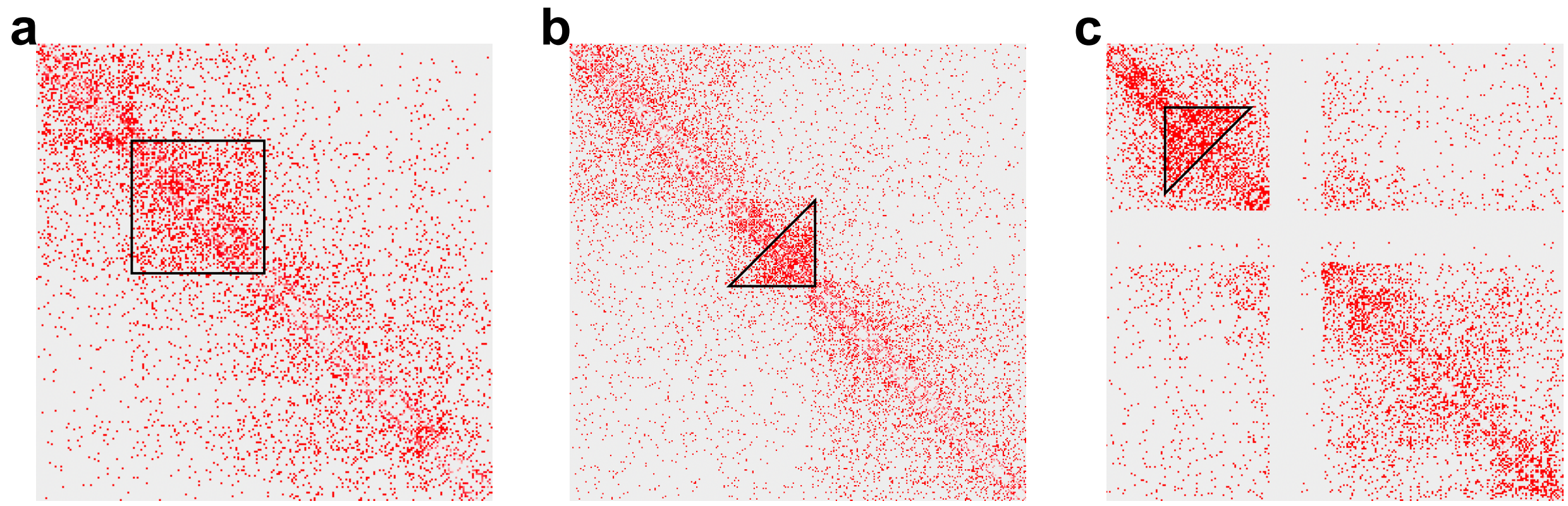

Supplementary Figure S35: Categorization of on-diagonal rectangles caused by contiguous domains. a-c, Common signal patterns of on-diagonal rectangles include uniform signals (a), stronger lower triangle (b), and stronger upper triangle (c).

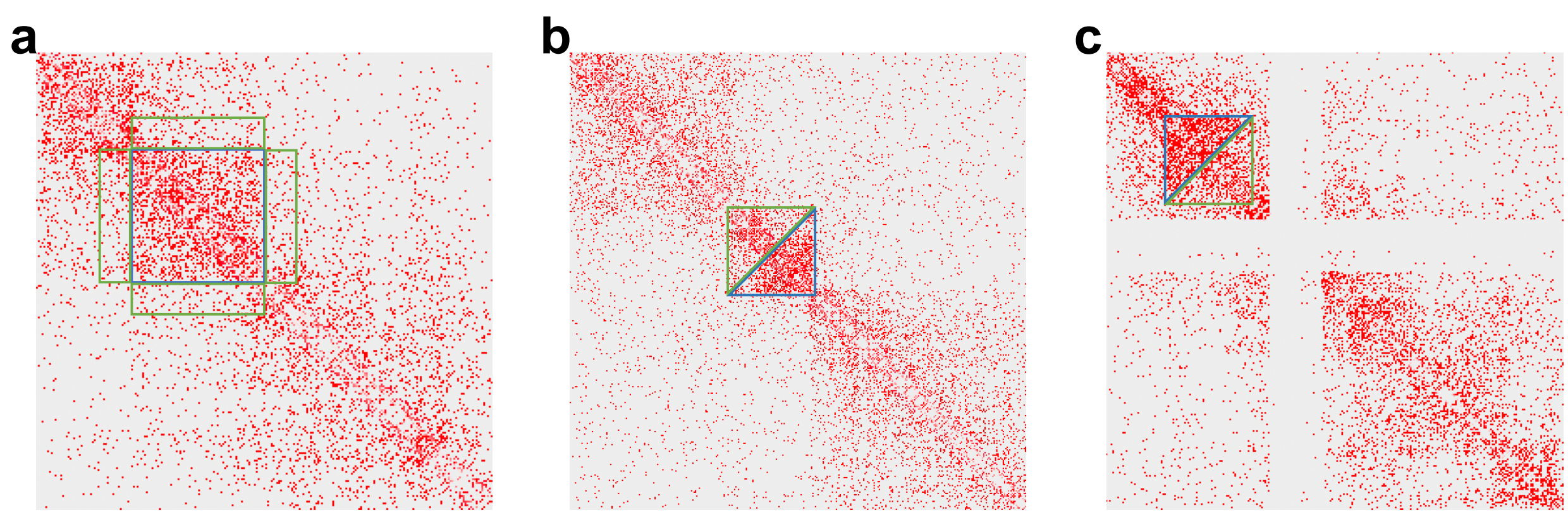

Supplementary Figure S36: Obtaining strong values and baseline values from on-diagonal blocks a-c, The blue and green lines show areas from which strong values and baseline values are obtained, respectively, for modeling on-diagonal blocks in the uniform signal category (a), lower triangle category (b), and upper triangle category (c).

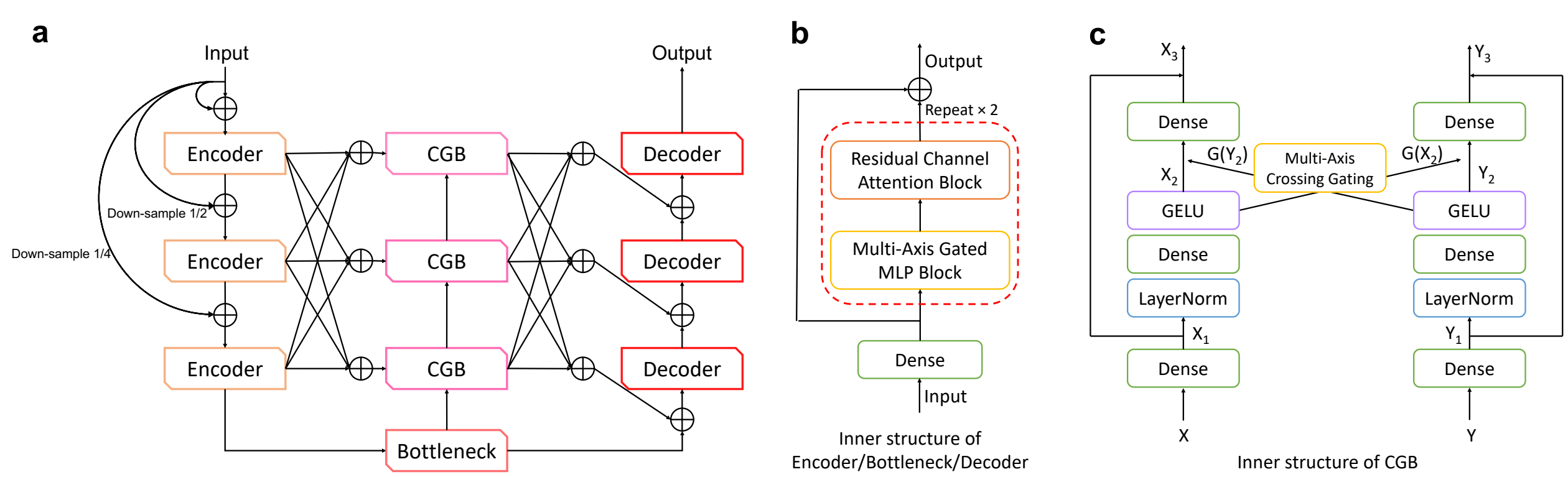

Supplementary Figure S37: The MAXIM architecture a, The overall design of MAXIM. **b,** Zoom-in view of a encoder, decoder, or bottleneck block. **c,** Zoom-in view of a GCB.

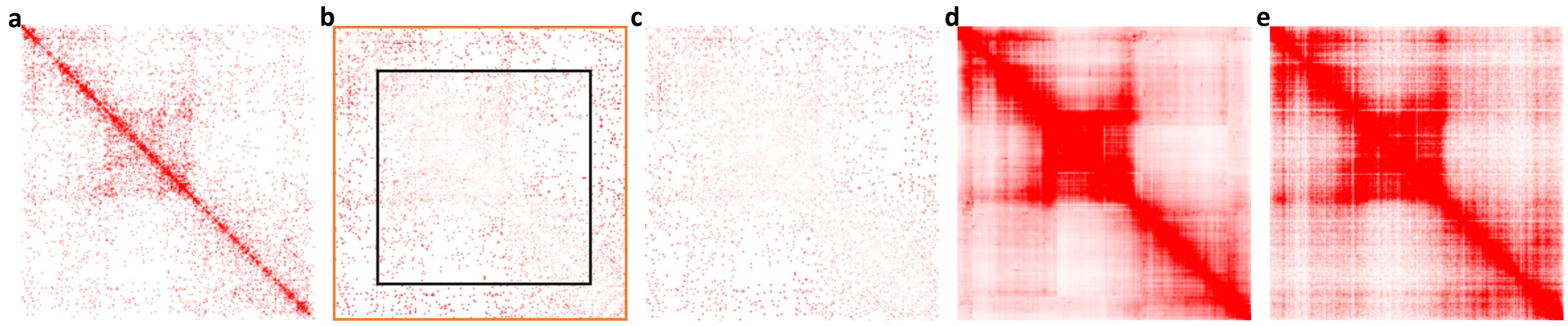

Supplementary Figure S38: Recoverable decomposition errors caused by imperfect rectangle boundary localization. **a,** The original low-resolution Hi-C map before decomposition. **b,** Residual contact map after removing the one-dimensional proximity effects and on-diagonal blocks. The largest detected off-diagonal rectangle (black box) does not fully cover the underlying interaction domain (orange box), leaving strong interaction signals outside the detected boundary. **c,** Residual contact map after subsequent rounds of off-diagonal rectangle detection (same color scale as panel b). Most of the remaining strong interaction signals are detected and removed, resulting in a more uniform residual map. **d,** Hi-C map reconstructed by SHARP. **e,** Corresponding actual high-resolution Hi-C map. The recovered interaction patterns closely match those in the ground truth.

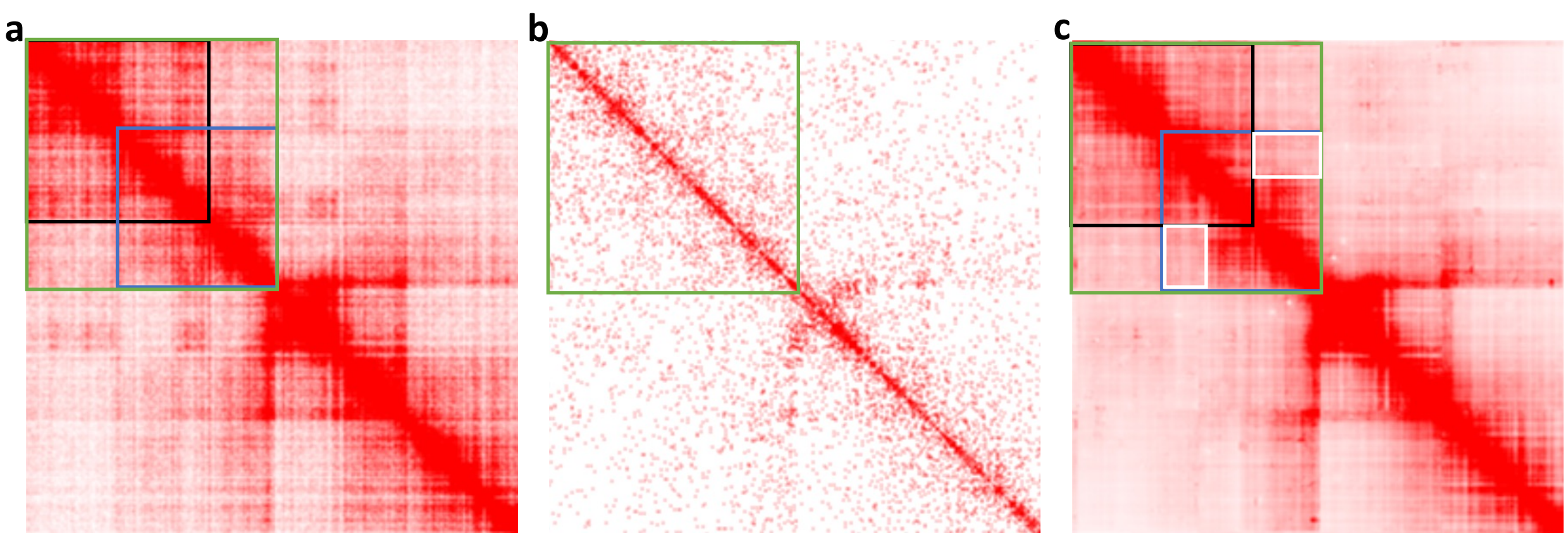

Supplementary Figure S39: Partially compensable decomposition errors under low-signal conditions. **a,** Corresponding actual high-resolution Hi-C map, showing that the green region consists of two distinct interaction subdomains. **b,** Low-resolution Hi-C map containing a weak interaction domain. Only the overall interaction region (green box) is clearly visible, while its two constituent subdomains (black and blue boxes) cannot be distinguished. **c,** Hi-C map reconstructed by SHARP. The stronger subdomain (black box) is successfully recovered, whereas the weaker subdomain (blue box) is only partially reconstructed, with several strong interaction signals near its upper-right and lower-left corners (outlined in white) remaining unrecovered.

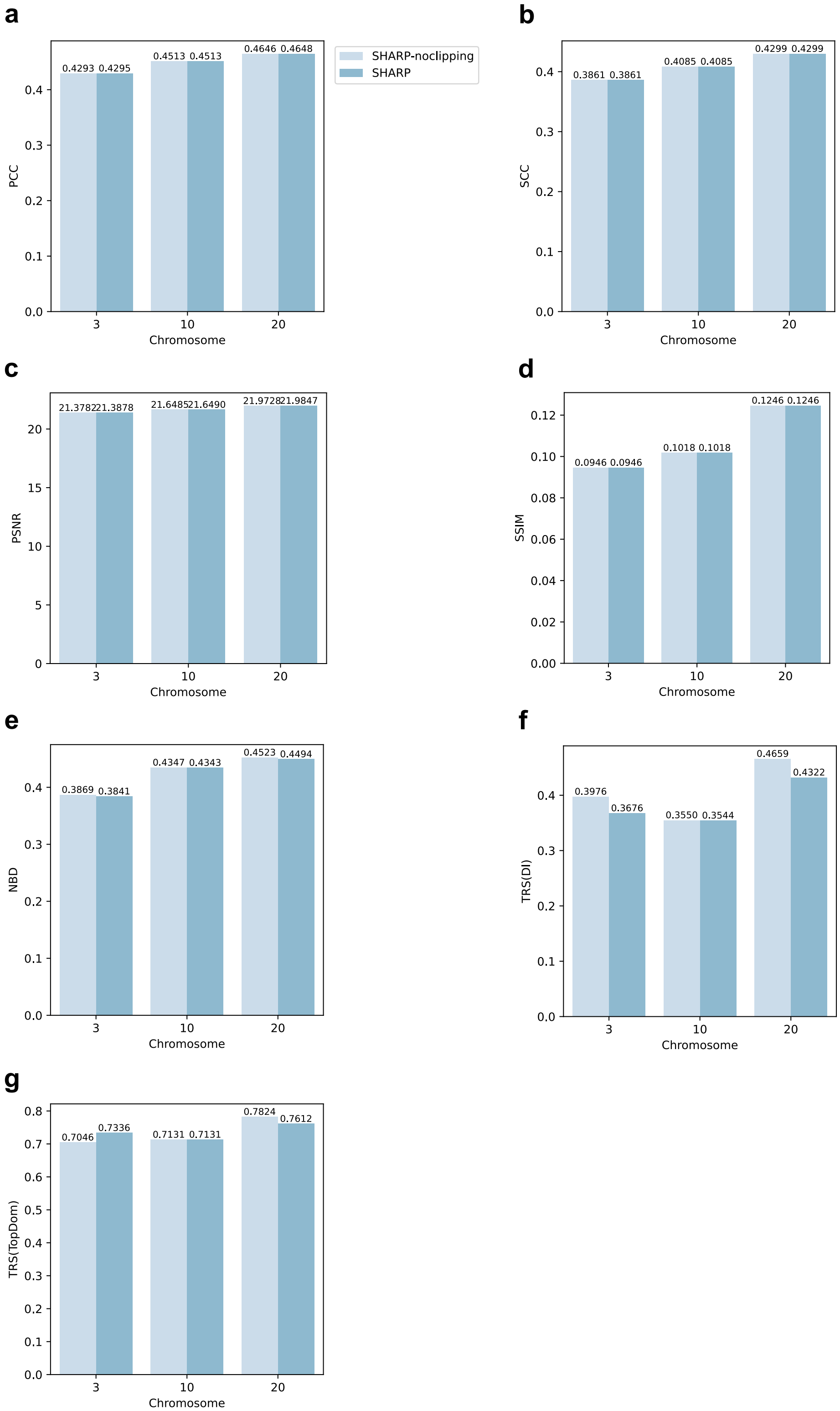

Supplementary Figure S40: Sensntivity test of comparing SHARP with SHARP-noclipping. a, PCC. **b,** SCC. **c,** PSNR. **d,** SSIM. **e,** NBD. **f,** TRS (DI). **g,** TRS (TopDom).

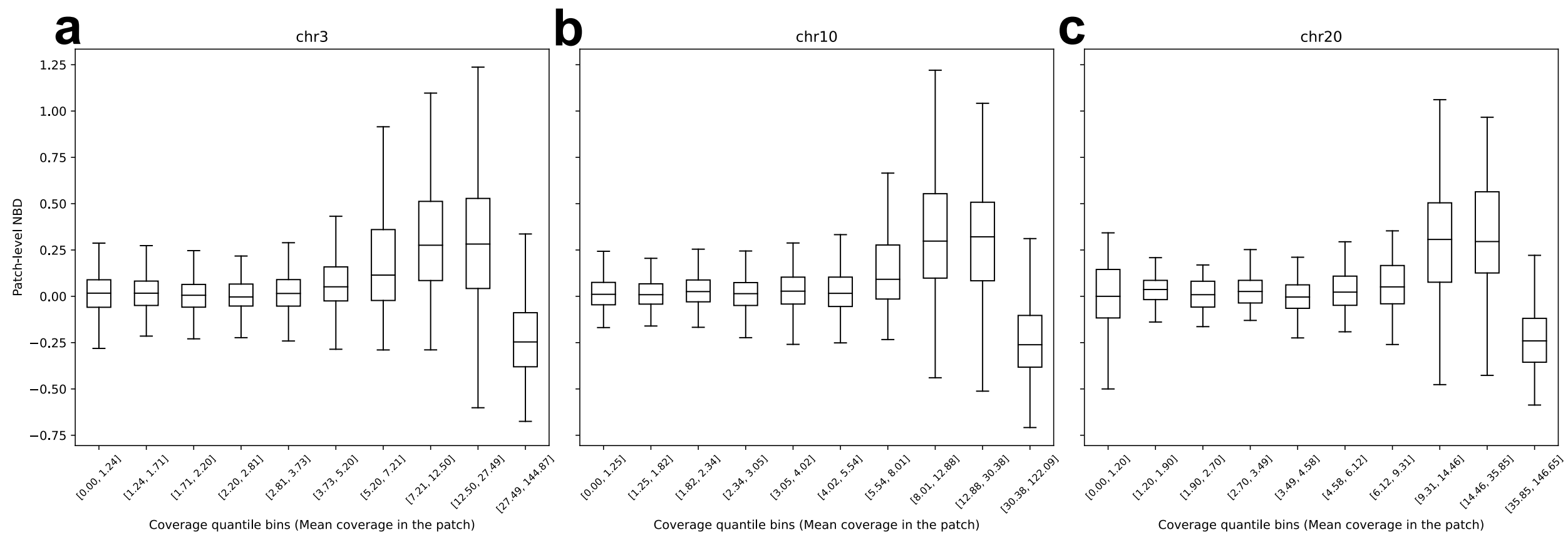

Supplementary Figure S41: Patch-level NBD distributions across different coverage groups measured in actual high-resolution maps. Patches were divided into quantile bins according to their mean contact intensity (coverage). **a,** Chr3. **b,** Chr10. **c,** Chr20.

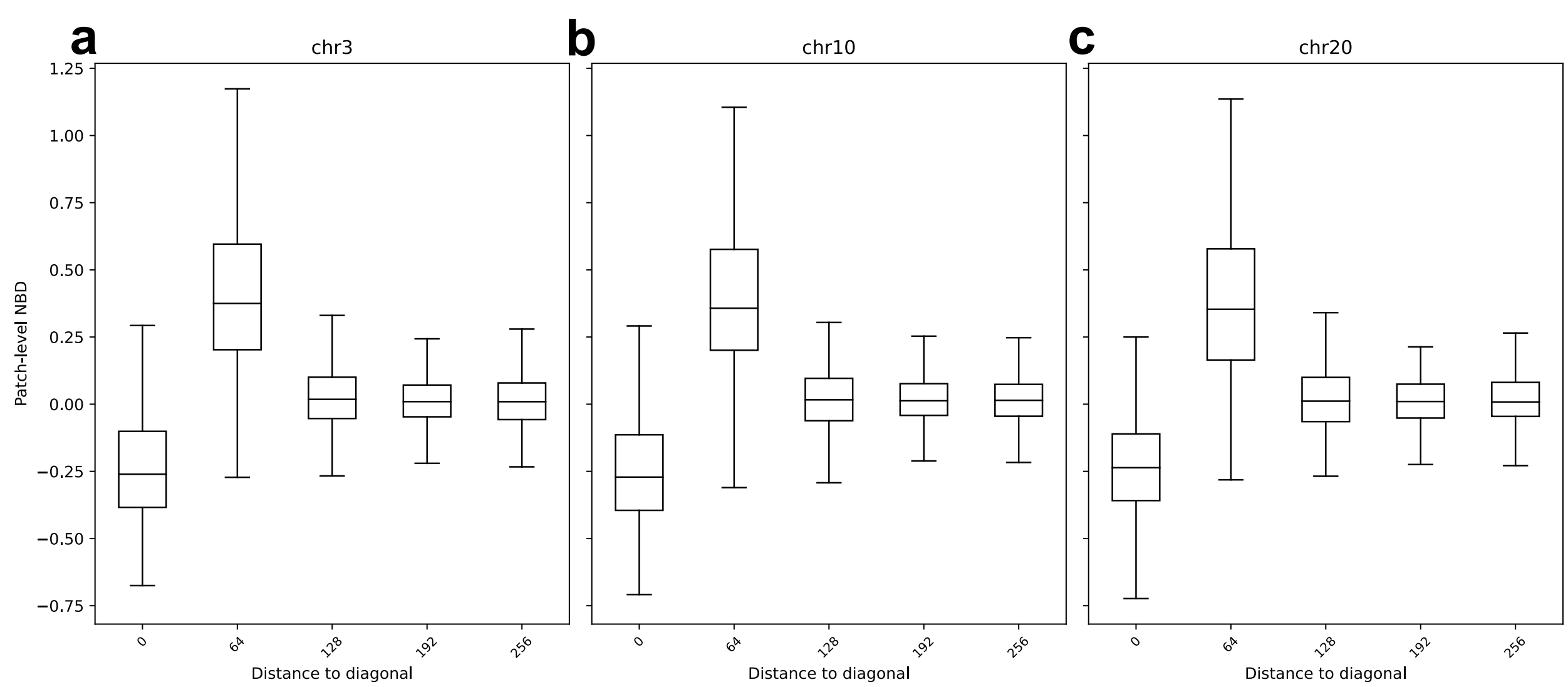

Supplementary Figure S42: Patch-level NBD distributions across different distances to the main diagonal measured in actual high-resolution maps. For each chromosome, the contact matrix was partitioned into 64×64 patches, and patch-level NBD values were computed for patches located at different distances from the diagonal. Distances were defined by the offset between the patch position and the main diagonal of the contact matrix. **a,** Chr3. **b,** Chr10. **c,** Chr20.

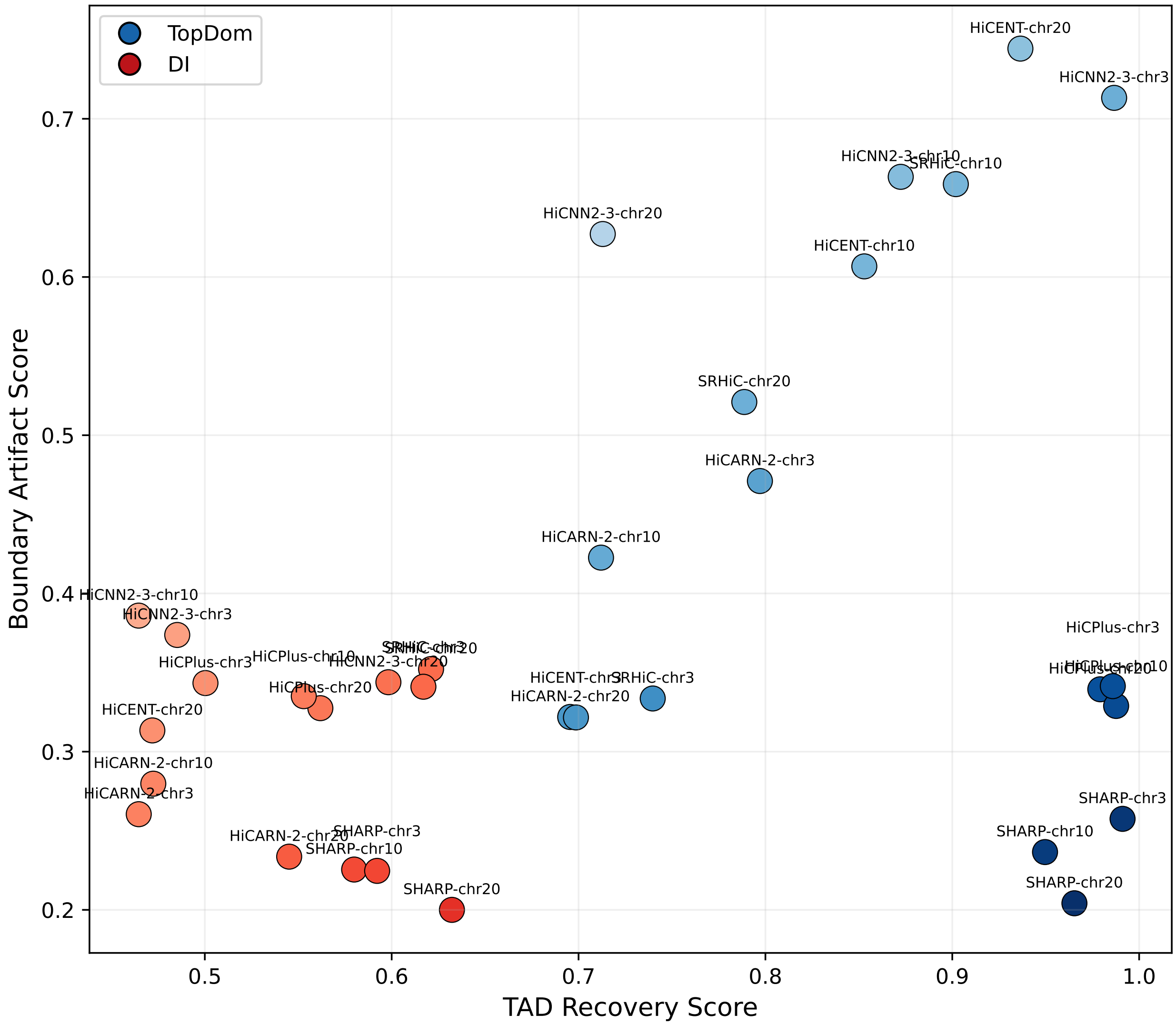

Supplementary Figure S43: Scatter plot of the TAD Recovery Score and Boundary Artifact Score for different reconstruction methods and chromosomes under the TopDom and DI methods. Each point represents one chromosome-specific reconstruction result, with color intensity indicating the overall TRS value (darker colors correspond to higher TRS). Blue points denote results evaluated using TopDom, while red points correspond to DI-based evaluation.
